# Aging alters the epigenetic asymmetry of HSC division

**DOI:** 10.1371/journal.pbio.2003389

**Published:** 2018-09-20

**Authors:** M. Carolina Florian, Markus Klose, Mehmet Sacma, Jelena Jablanovic, Luke Knudson, Kalpana J. Nattamai, Gina Marka, Angelika Vollmer, Karin Soller, Vadim Sakk, Nina Cabezas-Wallscheid, Yi Zheng, Medhanie A. Mulaw, Ingmar Glauche, Hartmut Geiger

**Affiliations:** 1 Institute of Molecular Medicine and Stem Cell Aging, University of Ulm, Ulm, Germany; 2 Institute for Medical Informatics and Biometry, Carl Gustav Carus Faculty of Medicine, Technische Universität Dresden, Dresden, Germany; 3 Max Planck Institute (MPI) of Immunobiology and Epigenetics, Freiburg, Germany; 4 Division of Experimental Hematology and Cancer Biology, Cincinnati Children's Hospital Medical Center, University of Cincinnati, Cincinnati, Ohio, United States of America; 5 Institute of Experimental Cancer Research, Medical Faculty, University of Ulm, Ulm, Germany; Cambridge Stem Cell Institute, United Kingdom of Great Britain and Northern Ireland

## Abstract

Hematopoietic stem cells (HSCs) balance self-renewal and differentiation to maintain homeostasis. With aging, the frequency of polar HSCs decreases. Cell polarity in HSCs is controlled by the activity of the small RhoGTPase cell division control protein 42 (Cdc42). Here we demonstrate—using a comprehensive set of paired daughter cell analyses that include single-cell 3D confocal imaging, single-cell transplants, single-cell RNA-seq, and single-cell transposase-accessible chromatin sequencing (ATAC-seq)—that the outcome of HSC divisions is strongly linked to the polarity status before mitosis, which is in turn determined by the level of the activity Cdc42 in stem cells. Aged apolar HSCs undergo preferentially self-renewing symmetric divisions, resulting in daughter stem cells with reduced regenerative capacity and lymphoid potential, while young polar HSCs undergo preferentially asymmetric divisions. Mathematical modeling in combination with experimental data implies a mechanistic role of the asymmetric sorting of Cdc42 in determining the potential of daughter cells via epigenetic mechanisms. Therefore, molecules that control HSC polarity might serve as modulators of the mode of stem cell division regulating the potential of daughter cells.

## Author summary

Stem cells are unique cells that can differentiate to produce more stem cells or other types of cells and can divide both symmetrically (to produce daughter cells with the same fate) and asymmetrically (to produce one daughter cell that retains stem cell potential and one that differentiates). The mechanisms that control the outcome of stem cell divisions have been the focus of many studies; however, they remain mainly unknown. Here, we have analyzed these mechanisms in murine hematopoietic stem cells (HSCs) by directly comparing the epigenetic signature, the transcriptome, and the function of the two daughter cells stemming from the first division of either a young or an aged HSC. We observe that, while young HSCs divide mainly asymmetrically, aged HSCs divide primarily symmetrically. We find that the mode of division is tightly linked to stem cell polarity and is regulated by the activity level of the small RhoGTPase cell division control protein 42 (Cdc42). In addition, we show that the potential of daughter cells is further linked to the amount of the epigenetic mark H4K16ac and also to the amount of open chromatin allocated to a daughter cell, but it is not linked to its transcriptome. In summary, our study suggests that HSC polarity linked to Cdc42 activity drives the mode of division, while epigenetic mechanisms determine the functional outcome of the stem cell division.

## Introduction

Hematopoietic homeostasis depends on the ability of hematopoietic stem cells (HSCs) to balance symmetric and asymmetric divisions over an organism’s lifespan. Asymmetric divisions allow one daughter cell to differentiate while the other retains its stem cell potential. In contrast, symmetric divisions result in daughter cells that adopt equivalent fates [1–8]. The asymmetric or symmetric distribution of cellular components to daughter cells during mitosis is thought to determine their fate. This concept has been proposed as a way of controlling the size of the HSC pool [9]. However, the mechanisms that control the mode and outcome of HSC divisions with respect to the potential and ultimately function of daughter cells remain incompletely understood.

While the number of phenotypic HSCs increases upon aging, their regenerative potential decreases, both in mice and in humans [10–13]. Aged HSCs differentiate preferentially into myeloid cells and less into cells of the lymphoid lineage. Aging of HSCs is caused by intrinsic changes in HSCs and also by extrinsic factors from the niche [14–20]. Epigenetic modifications and altered gene expression profiles (e.g., high expression of myeloid genes), together with loss of the polar distribution of tubulin and cell division control protein 42 (Cdc42) in the cytoplasm and loss of the polarity of the acetylated form of the epigenetic marker H4K16 (H4K16ac) in the nucleus, are hallmarks of intrinsic HSC aging [10, 21–23]. Cdc42 is a small RhoGTPase that cycles between an active guanosine triphosphate (GTP)-bound state and an inactive guanosine diphosphate (GDP)-bound state. A central, HSC-intrinsic molecular mechanism that causes aging of HSCs and that results in the above-mentioned phenotypic and functional impairments of aged HSCs is an increase in the activity of Cdc42 in aged stem cells [10, 15, 16, 24, 25]. Consequently, treatment of aged HSCs ex vivo with a Cdc42 activity specific inhibitor (CASIN), which reduces the age-associated increase in Cdc42 activity, increased polarity in aged HSCs and rejuvenated their function in vivo [16]. The increase in Cdc42 activity in aged HSCs is caused by an intrinsic increase in the level of Wnt5a in HSCs [15]. Treatment of young HSCs with Wnt5a induced elevated activity of Cdc42 and a loss of cell polarity and premature aging of HSCs [15].

We hypothesized here that HSC polarity determines the mode (symmetry/asymmetry) of HSC divisions and therefore that there is a difference in the symmetry/asymmetry of division upon changes in the activity level of Cdc42 in HSCs. We thus investigated whether the targeted alteration of Cdc42 activity by either CASIN (to decrease Cdc42-GTP levels in aged HSCs) or Wnt5a treatment (to increase Cdc42-GTP levels in young HSCs), which translates into polarity changes, influences the balance of asymmetric or symmetric HSC divisions (mode of division) and therefore drives functional differences among daughter cells (output of division) upon aging and rejuvenation (S1A Fig). By comparing the mode of HSC division in vitro and the output of division in vivo, we demonstrate that young and aged CASIN-treated HSCs divide asymmetrically while aged and young Wnt5a-treated HSCs undergo symmetric divisions. We conclude that, by modulating Cdc42 activity and consequently cell polarity, it is possible to adjust the mode and the epigenetic outcome of HSC divisions.

## Results

### Young HSCs divide asymmetrically, whereas aged HSCs divide symmetrically

**Fig 1:**
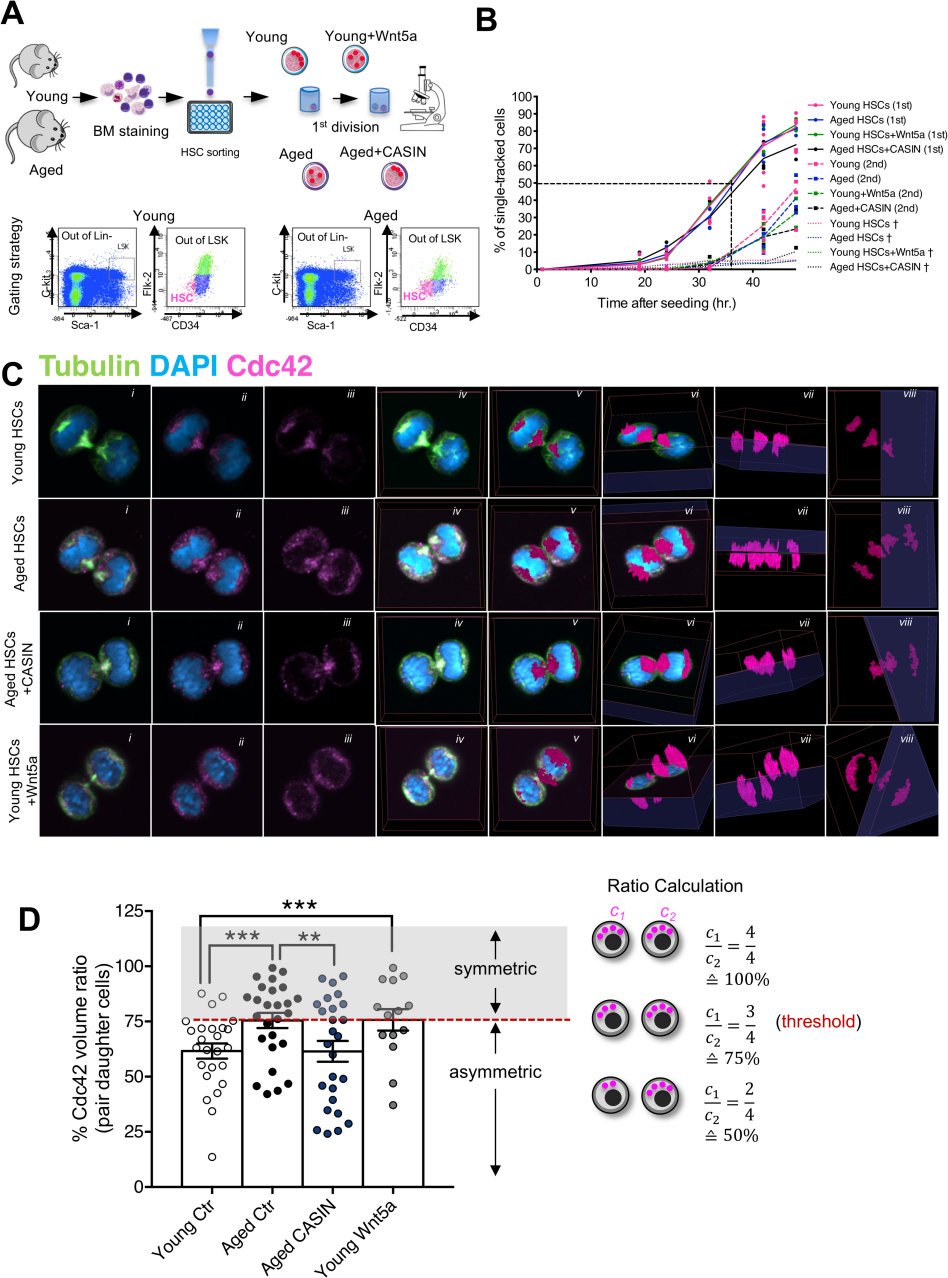
Young and aged CASIN-treated HSCs allocate Cdc42 asymmetrically to daughter cells, while aged and young Wnt5a-treated HSCs allocate it symmetrically. (A) Schematic representation of the gating strategy and of the experimental setup (graphical sources: https://www.servier.de/medical-art and https://openclipart.org/). (B) Percentage of HSCs that divided over time (1st: first divisions; 2nd: second divisions). Percentage of dead cells is also plotted (indicated with †). (C) Representative confocal single *z*-stack (central *xy* plane; i–iii), 2D *xy* plane projection of all *z*-stacks (iv), 3D reconstruction of Cdc42 signal overlapped on the 2D projection of all *z*-stacks, (v) and rotation of the 3D Cdc42 reconstructed signal (vi–viii) of dividing (telophase) young, aged, aged treated with CASIN 5 μM, and young treated with Wnt5a 100 ng/mL HSCs. Panels show DAPI (nucleus, blue), Cdc42 (magenta), and tubulin (green). (D) Percentage of the ratio of Cdc42 (quantified as volume of the fluorescence signal) amount in the daughter cells (higher amount over the lower amount). Each dot represents a pair. Only telophases and late anaphases were considered for the analysis. Cartoon depicting the scoring for symmetric and asymmetric divisions according to the amount of inherited protein as analyzed by 3D confocal microscopy. *p < 0.05, **p < 0.01, ***p < 0.001; *n* = 2–3 biological repeats; 25 pairs for young, 26 pairs for aged, 26 pairs for aged + CASIN, and 14 pairs for young + Wnt5a. The primary data the figure is based on are provided in S1 Data. CASIN, Cdc42 activity specific inhibitor; Cdc42, cell division control protein 42; Ctrl, control; HSC, hematopoietic stem cell.

We first microscopically tracked the kinetics of single HSC doubling in vitro (Fig 1A).

**Fig 2:**
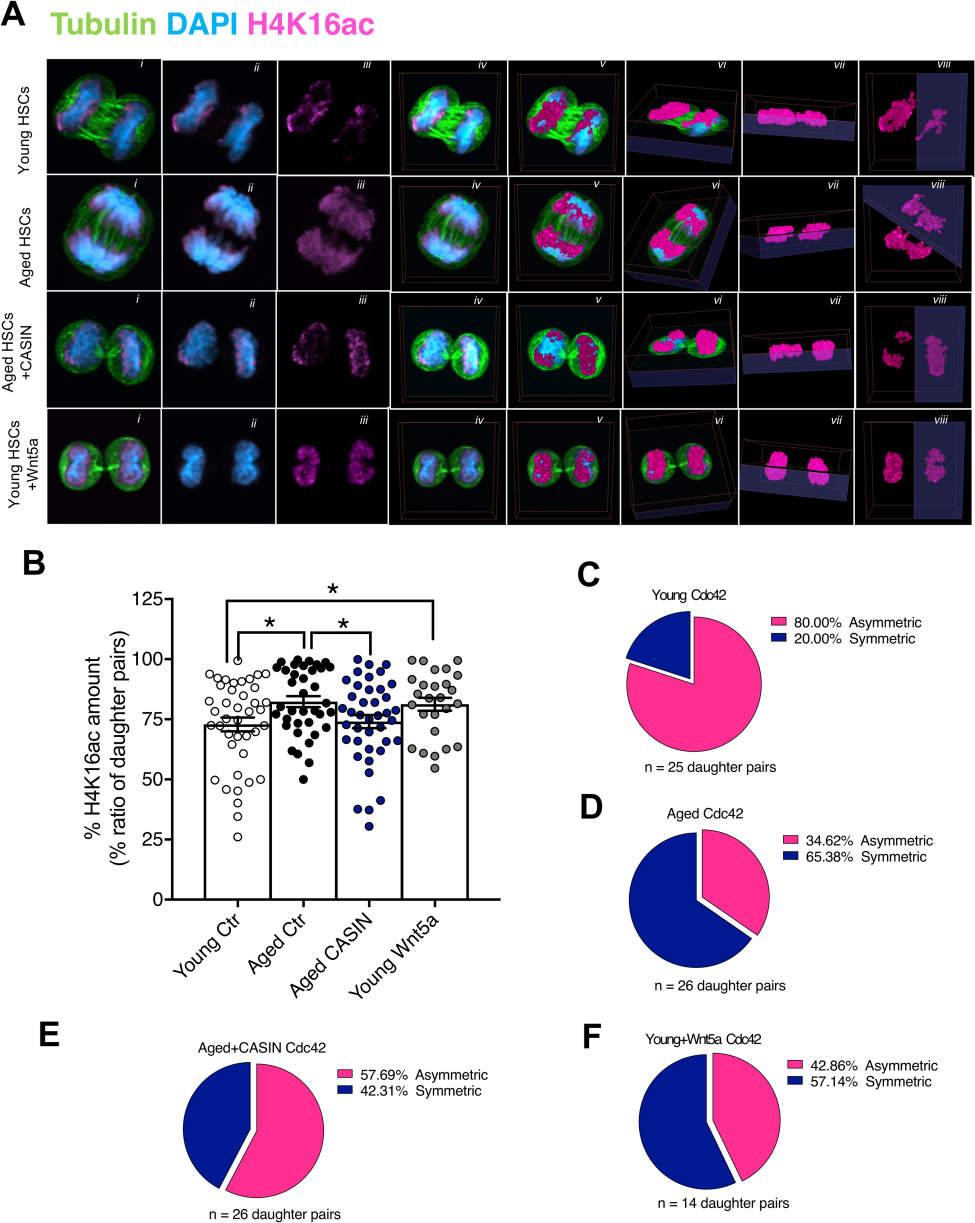
Young and aged CASIN-treated HSCs allocate H4K16ac asymmetrically to daughter cells, while aged and young Wnt5a-treated HSCs allocate it symmetrically. (A) Representative confocal single *z*-stack (central *xy* plane [i–iii]), 2D *xy* plane projection of all *z*-stacks (iv), 3D reconstruction of H4K16ac signal overlapped on the 2D projection of all *z*-stacks (v) and rotation of the 3D H4K16ac reconstructed signal (vi–viii) of dividing (telophase) young, aged, aged treated with CASIN 5 μM, and young treated with Wnt5a 100 ng/mL HSCs. Panels show DAPI (nucleus, blue), H4K16ac (magenta), and tubulin (green). (B), Percentage of the ratio of H4K16ac (quantified as volume of the fluorescence signal) in the daughter cells (higher amount over the lower). Each dot represents a pair. Only telophases and late anaphases were considered for the analysis. *p < 0.05, **p < 0.01, ***p < 0.001; *n* = 2–3 biological repeats; 41 pairs for young, 37 pairs for aged, 40 pairs for aged + CASIN, and 26 pairs for young + Wnt5a. (C–F) Pie charts depicting the frequency of asymmetric/symmetric division in each sample based on the amount of Cdc42 in daughter cells. Any division with a ratio (see cartoon scheme in Fig 1D) equal to or below 75% was considered asymmetric. The primary data the figure is based on are provided in S1 Data. CASIN, Cdc42 activity specific inhibitor; Cdc42, cell division control protein 42; Ctr, control; HSC, hematopoietic stem cell.

There was no difference in the kinetics of first and second divisions between young, aged, aged treated with CASIN, and young treated with Wnt5a HSCs (Fig 1B) nor in the relative distribution of mitotic cells to metaphase, anaphase, or telophase (S1B Fig). Next, 3D confocal immunofluorescence (IF) was used to quantify Cdc42 and H4K16ac allocation to daughter cells during mitosis. Young HSCs were found to segregate Cdc42 and H4K16ac asymmetrically into daughter cells. Aged HSCs displayed a symmetric distribution of both Cdc42 and H4K16ac in daughter cells, and inhibition of Cdc42 activity in aged HSCs increased the frequency of asymmetric divisions among aged HSCs. Consistently, in the presence of Wnt5a, young HSCs presented a shift towards a symmetric allocation (Fig 1C and 1D and Fig 2A and 2B; S1C–S1J Fig; S1–S8 Movies; S1 Table; S2 Fig).

To note, polarity was not affected by cell culture conditions (S1K Fig), and Cdc42 protein was observed asymmetrically or symmetrically already during metaphase (S1L Fig). H4K16ac levels were stably detected throughout mitosis (S1M Fig), and the difference in allocation was quantified at telophase. When setting the threshold to a 75% difference in Cdc42 amount between daughter cell pairs (which translates into at ratio of 1 over 4, see also cartoon scheme in Fig 1D), 80% of young HSCs divided asymmetrically (Fig 2C). The frequency of asymmetric division was significantly reduced upon aging (S1 Table and Fig 2D). Consistent with polarity/apolarity before division, aged CASIN-treated HSCs divided more frequently asymmetrically, while Wnt5a treatment of young HSCs increased the frequency of symmetric divisions (Fig 2E and 2F).

### HSC polarity is strongly associated with the mode of division and predicted to drive an asymmetric outcome in daughter cells

**Fig 3:**
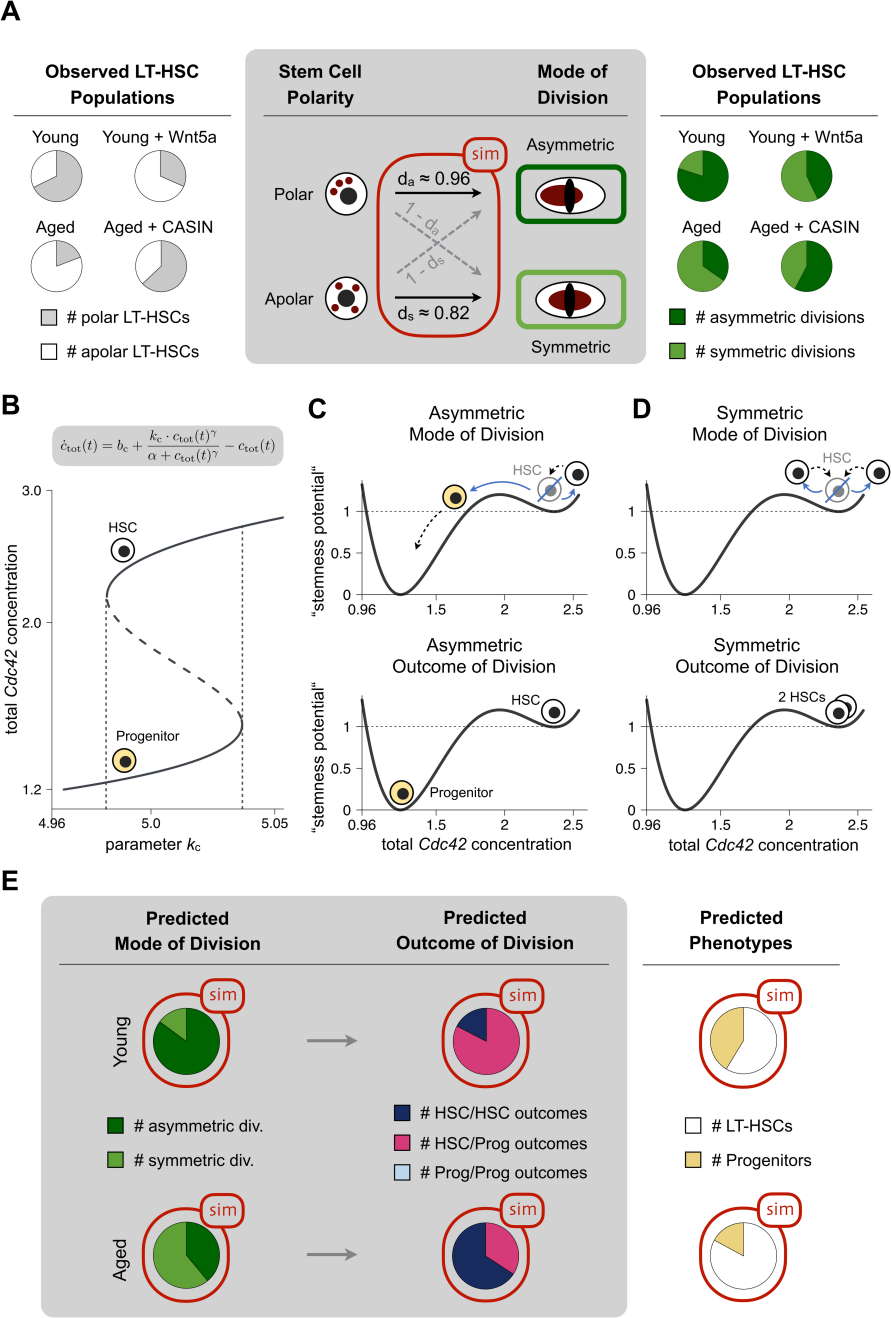
Polarity is a major driver of asymmetric cell division, and Cdc42 allocation predicts potential. (A) Pie charts in white/grey show the observed ratios of polar versus apolar HSCs in young, aged, Wnt5a-treated young, and CASIN-treated aged HSC samples, respectively [15, 16]. Pie charts in green show the observed modes of division for the respective cell populations (see Fig 2C–2E). The intermediate panel depicts the possible connections (arrows) between cell polarity status and mode of division. Application of a maximum likelihood approach on a simple transition model (with *d*_*a*_ being the probability for a polar HSC to undergo an asymmetric mode of division and *d*_*s*_ being the probability for an apolar HSC to undergo a symmetric mode of division) allows estimating the most probable configuration for *d*_*a*_ and *d*_*s*_, thereby supporting the notion that the solid arrows represent the most prominent choices. (B) The bifurcation diagram illustrates the differentiation switch. For certain parameter values of *k*_*c*_, the ODE for total Cdc42 concentration *c*_tot_(*t*) allows for 2 stable steady states: (I) total Cdc42 is highly expressed (associated with the HSC state) and (II) total Cdc42 is lowly expressed (associated with the progenitor state). (C, D) The pseudo-potential (“stemness potential”) landscape for total Cdc42 concentration with two stable steady states (two “valleys,” representing HSCs and progenitors). For HSCs undergoing asymmetric cell division (blue line in panel C), one daughter receives lower Cdc42 concentration (“passes the hill”) and progresses into the progenitor state (dashed arrow), while the other daughter retains the stem cell state. Daughters of HSCs undergoing symmetric division (blue line in panel D) retain their configuration. (E) The panels summarise the mechanistic model results (red boxes) for populations of young and aged HSCs, respectively. The pie diagrams on the right-hand side show the predicted ratios of resulting cell types (HSCs versus progenitors) derived from a population of dividing young and aged HSCs, respectively. The primary data the figure is based on are provided in S1 Data. CASIN, Cdc42 activity specific inhibitor; Cdc42, cell division control protein 42; HSC, hematopoietic stem cell; ODE, ordinary differential equation.

Because it is not yet possible to image in living cells Cdc42 and H4K16ac allocation to nascent daughter cells, we used mathematical association analyses to investigate whether there is a link between polarity in mother HSCs and the mode of HSC division. The probability that a polar stem cell will undergo an asymmetric division was termed *d*_*a*_ (with probability for symmetric division defined as 1 − *d*_*a*_). Similarly, *d*_*s*_ represented the probability that an apolar stem cell divides symmetrically (with 1 − *d*_*s*_ for asymmetric) (Fig 3A).

By applying a maximum likelihood estimation to the previously published observed frequencies of polar versus apolar HSCs in young, aged, young Wnt5a-treated, and aged CASIN-treated cell populations [15, 16], we found that the probabilities *d*_*a*_ ≈ 0.96 and *d*_*s*_ ≈ 0.82 best explained the corresponding modes of division (Fig 3A). Modelling thus indicates that there is a 96% chance that a polar cell will divide asymmetrically, and apolar cells will divide with an 82% chance symmetrically.

Encouraged by this strong association between HSC polarity/apolarity and asymmetric/symmetric mode of division, we further designed a mechanistic mathematical model to investigate the causative link to the fates of the daughter cells (see S1 Text and S3 Fig). To mimic the experimental observations, we assumed that Cdc42 is either distributed uniformly (apolar) or peaked (polar) along a circular, abstracted cell shape. We directly coupled the active proportion of intracellular Cdc42 (Cdc42-GTP) to the shape of this distribution, thereby functionally connecting the observed increase in Cdc42 activity with the loss of polarity during aging [15]. The mathematical approach suggested that a switch-like construct could determine daughter cell fate, in which different fates manifest based on the intracellular protein concentration immediately after cell division. We also implemented a transcriptional autoregulative feedback for total Cdc42 concentration, thereby establishing bistability (Fig 3B). For certain parameter choices, such a system yielded 2 characteristic states: either Cdc42 was highly expressed due to self-activation (e.g., indicative of HSCs) or Cdc42 was lowly expressed (e.g., indicative of the progenitor state) (Fig 3C and 3D). The choice of either the HSC or the progenitor state was determined by Cdc42 concentration right after cell division. Distinct concentrations between daughter cells due to an asymmetric mode of division resulted also in distinct states (Fig 3C and 3D). Because Cdc42 activity, and thereby HSC polarity, is altered during aging, the model predicted that asymmetric outcomes of division are more likely for young HSCs, while a higher incidence of symmetric divisions in aged HSCs will preferentially promote self-renewal, predicting a progressive expansion of the stem cell pool over time (Fig 3E). Indeed, an increase in the number of HSCs in aged mice and humans has been verified by several groups [8, 15, 16, 22, 26–28].

### Restoring asymmetry to divisions of aged HSCs results in rejuvenated daughter stem cells

**Fig 4:**
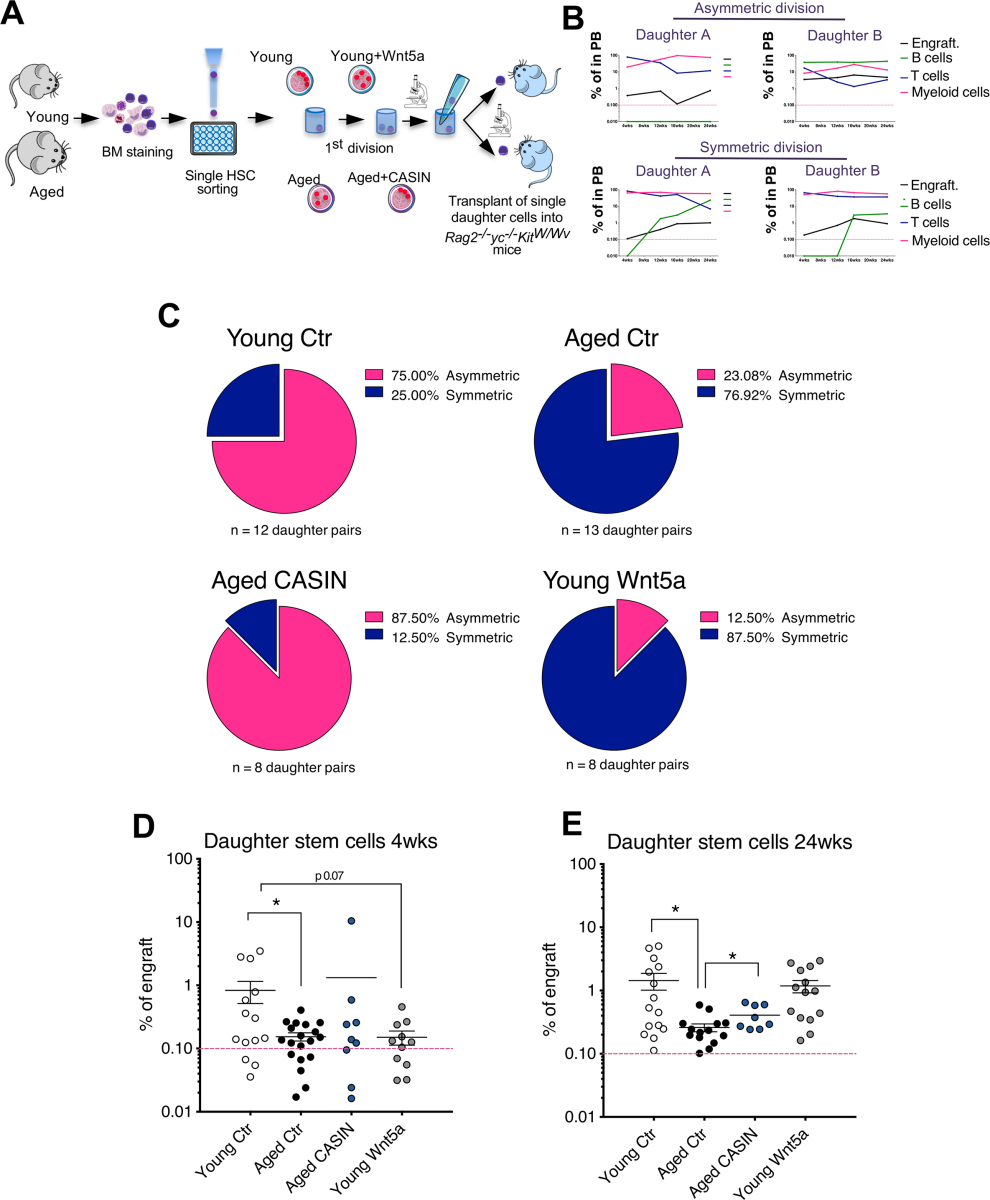
Restoring the asymmetry of aged HSC divisions rejuvenates the function of daughter stem cells. (A) Schematic representation of the experimental setup (graphical sources: https://www.servier.de/medical-art and https://openclipart.org/). (B) Representative asymmetric and symmetric division based on the contribution of the single daughter cell to PB. Each single daughter cell was injected into 1 recipient mouse and donor-derived Ly5.1^+^ cells (engraft) B220^+^, CD3^+^, and myeloid (Gr1^+^, Mac1^+^, and Gr1^+^Mac1^+^) cells among Ly5.1^+^ donor-derived cells were measured at 4, 12, 16, and 24 weeks after transplant. (C) Pie charts depicting the percentage of asymmetric and symmetric divisions of young, aged, aged treated with CASIN 5 μM and young treated with Wnt5a 100 ng/mL HSCs based on the profile of the reconstitution in PB of single daughter cell pairs. *n* = 12 pairs for young HSCs, *n* = 13 pairs for aged HSCs, *n* = 8 pairs for aged CASIN-treated HSCs, and *n* = 8 pairs for young Wnt5a-treated HSCs. (D–E) Percentage of donor-derived cells at 4 weeks and 24 weeks after transplant in PB of mice transplanted with single (retrospectively identified) daughter stem cells. *p < 0.05. The primary data the figure is based on are provided in S1 Data. CASIN, Cdc42 activity specific inhibitor; Cdc42, cell division control protein 42; Ctr, control; HSC, hematopoietic stem cell; PB, peripheral blood.

To experimentally verify the predictions of the mathematical models and to further determine the function of daughter cells, we performed single daughter cell transplants using *Rag2*−/−γ*c*^−/−^*Kit*^W/Wv^ recipient mice [29]. After the first division in vitro, we injected each single daughter cell into an individual recipient mouse (Fig 4A) and assessed its contribution to peripheral blood (PB) every 4 to 8 weeks up to 24 weeks after transplantation.

**Fig 5:**
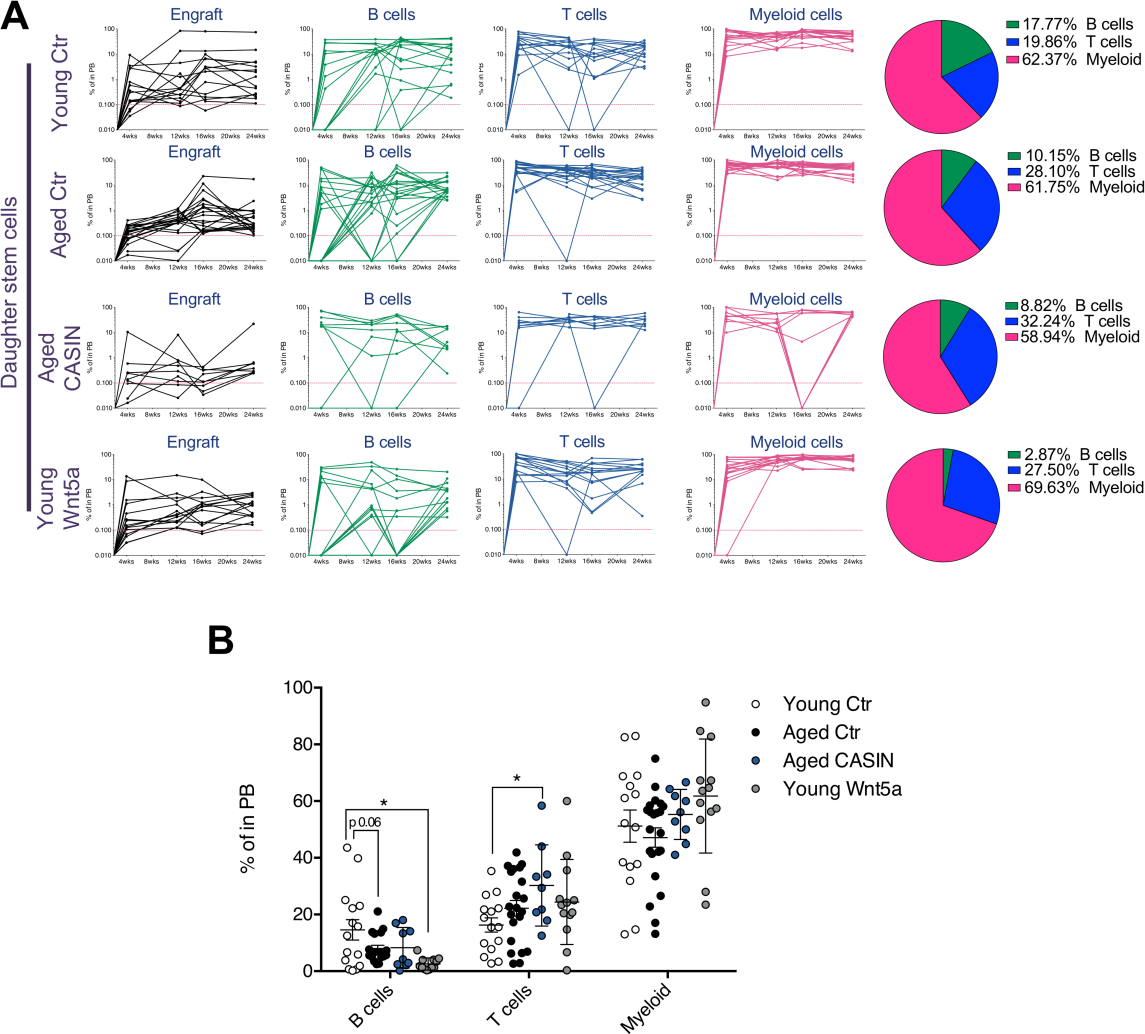
Kinetics of engraftment and lineage contribution of single daughter stem cells. (A) Chimerism kinetics of overall engrafted donor-derived cells and of each donor-derived lineage (B cells, T cells, and myeloid cells). Shown are young, aged, aged treated with CASIN, and young treated with Wnt5a daughter cells that were retrospectively identified as daughter stem cells. The pie charts represent the relative contribution to B cells, T cells, and myeloid cells in PB at 24 weeks after transplant. (B) Percentages of donor-derived B cells, T cells, and myeloid cells in PB of recipient mice 24 weeks after transplant. *n* = 9–22. Shown are young, aged, aged treated with CASIN, and young treated with Wnt5a daughter cells that were retrospectively identified as daughter stem cells. *p < 0.05. The primary data the figure is based on are provided in S1 Data. CASIN, Cdc42 activity specific inhibitor; Cdc42, cell division control protein 42; Ctr, control; PB, peripheral blood.

A daughter cell that was able to contribute to B cells, T cells, and myeloid cells 24 weeks after transplantation with an overall engraftment of more than 0.1% qualified as a stem cell [30, 31]. When a contribution at 24 weeks was not detected in all cell lineages tested or the overall contribution of all donor-derived cells was below 0.1%, the initially transplanted cell was scored as a progenitor cell. Based on the scores of the outcomes of the daughter cell transplants, the initial HSC division was then defined as symmetric (two daughter stem cells or two daughter progenitor cells) or asymmetric (one daughter stem cell and one daughter progenitor cell) (Fig 4B; S4A–S4D Fig; S5A Fig). Young HSCs divided primarily asymmetrically, while aged HSCs showed mainly symmetric division outcomes (Fig 4C; S2 and S3 Tables; S5A Fig). Treating aged HSCs with CASIN significantly increased the frequency of asymmetric divisions of aged HSCs; in contrast, treating young HSCs with Wnt5a (Cdc42 inducer) increased the frequency of symmetric divisions (Fig 4C; S2 and S3 Tables; S5A Fig). The highest overall contribution to PB was detected in mice transplanted with young daughter stem cells at both early and late time points after transplantation (Fig 4D and 4E; S5A Fig). Young Wnt5a-treated daughter stem cells presented with a decrease in engraftment only at early time points (4 weeks), while there was no difference in engraftment between young and young Wnt5a-treated daughter stem cells at the end point (24 weeks) (Fig 4D and 4E). Aged daughter stem cells from CASIN-treated mother HSCs showed an about 2-fold increase in overall engraftment compared to daughter stem cells from aged untreated HSCs (Fig 4E; S5A Fig). Young daughter stem cells presented with the highest level of contribution to B cells, while aged and young Wnt5a-treated cells displayed a significantly reduced B cell output (Fig 5A and 5B; S5A Fig).

Daughter stem cells from aged CASIN-treated HSCs differentiated significantly more than the other experimental samples into T cells (Fig 5A and 5B; S5A Fig). As for daughter progenitor cells, no difference was detected among samples, besides the observation that young and aged CASIN-treated sample sets presented with a trend in higher reconstitution at 24 weeks after transplant (S5B–S5D Fig). Our data further demonstrate that the initial population of mother HSCs the experiments were initiated with contained, on average, 77.5% of cells with stem cell function (based on their ability to give rise to at least one daughter stem cell), and there was no difference between the groups of young and aged HSCs and treated HSCs (S3 Table and S6 Fig).

### Aged HSCs form clusters within bone marrow

**Fig 6:**
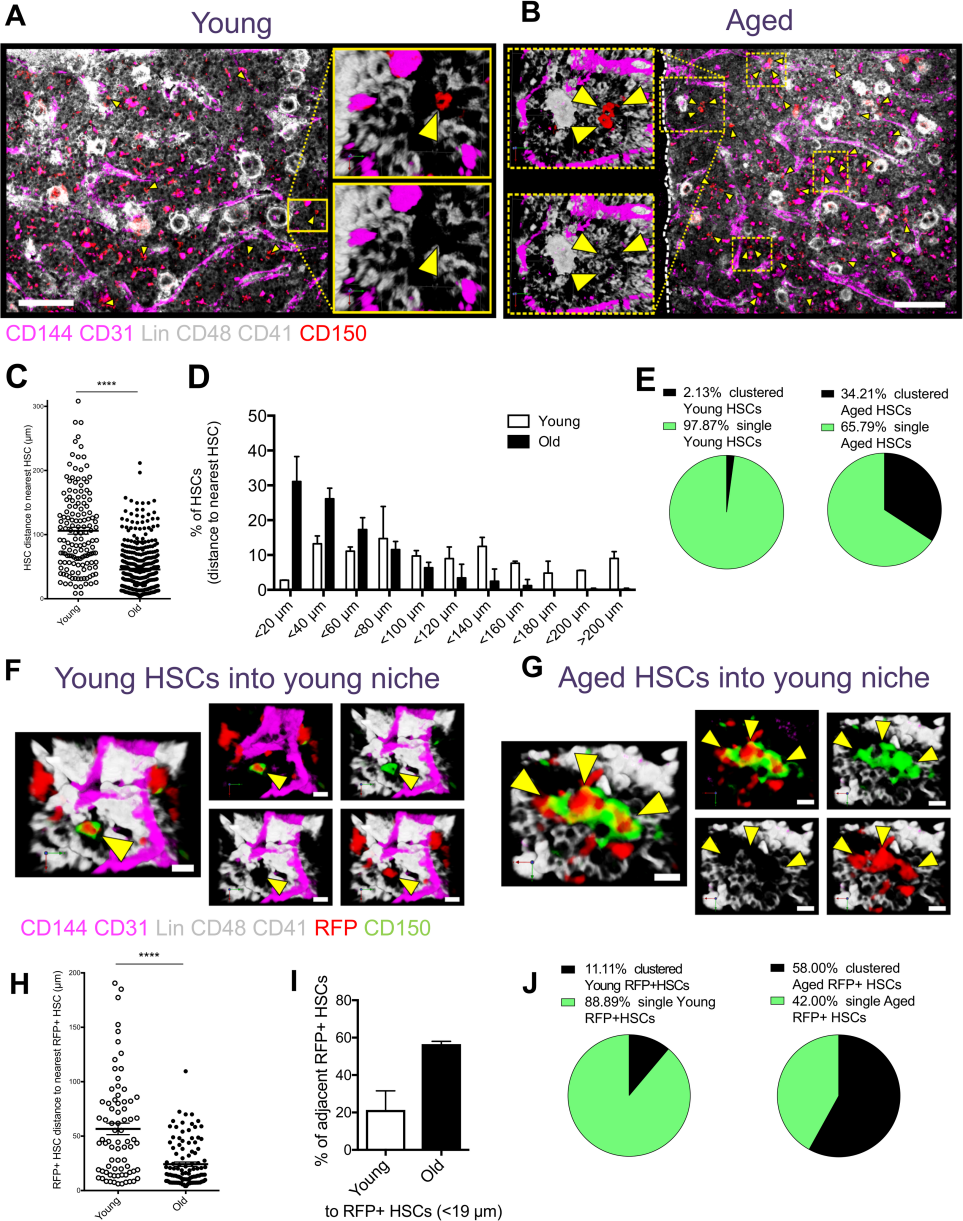
HSCs are found in clusters in bone marrow in aged animals. (A, B) Representative 3D confocal pictures of a whole-mount femur from a young (panel A) and an aged (panel B) mouse. HSCs are indicated by yellow arrowheads. Dashed lines in panel B highlight clustered HSCs. Lineage markers CD41 and CD48 are shown in white; CD150 in red and endothelial cells (IV injected VE-Cadherin CD144 and PECAM-1 CD31) are in magenta. (C) Distance between HSCs in young and aged femurs. (D) Percentage of HSCs located in 20 μm intervals from the closest HSC in young and aged femurs. (E) Pie charts depicting the percentage of HSCs found in clusters (2 cells or more adjacent to each other) or as single cell in young and aged mice. Imaging data refer to 27 young and 14 aged longitudinal shaved whole-mount cross-sections from 2 young and 2 aged mice; 144 young HSCs and 394 aged HSCs. (F, G) Representative 3D confocal pictures of whole-mount femur analyses from young recipient mice transplanted with young (panel A) and aged (panel B) RFP+ HSCs. Donor-derived RFP+ HSCs are marked by yellow arrowheads. Lineage markers CD41 and CD48 are shown in white, CD150 in green and endothelial cells (IV injected VE-Cadherin CD144 and PECAM-1 CD31) are in magenta. All donor-derived cells are RFP+ (red). (H) Distance between donor-derived RFP+ young and aged HSCs in the femurs of young recipient mice. (I) Percentage of donor-derived RFP+ young and aged HSCs located in 20 μm intervals from the closest donor-derived RFP+ HSC in the femurs of recipient mice. (J) Pie charts depicting the percentage of donor-derived RFP+ young and aged HSCs found in clusters (2 cells or more adjacent to each other) or as single cell in the femurs of young recipient mice. Imaging data refer to 8 young and 4 aged longitudinal shaved whole-mount cross-sections from 2 young and 2 aged mice; 75 young HSCs and 108 aged HSCs. The primary data the figure is based on are provided in S1 Data. HSC, hematopoietic stem cell; IV, intravenous; Lin, lineage; RFP, red fluorescent protein.

Frequent symmetric divisions of aged HSCs might result in a local accumulation of HSCs in bone marrow. We set up experiments to test this hypothesis and stained whole-mount preparations of the long bones of young and aged mice (S7A Fig). Indeed, in aged animals, HSCs often were found in clusters of 2 to 3 stem cells, while HSCs in young animals were almost always found as solitary cells (Fig 6A and 6B).

As a consequence, the distance of HSCs to their nearest neighbor HSC was significantly decreased in aged bones, and around 30% of all HSCs were found less than 20 μm (proximity) from another HSC (Fig 6C and 6D and S7B and S7C Fig). This translated into 34% of all aged HSCs being in clusters compared to less than 2% for young HSCs (Fig 6E and S7D Fig). We next investigated whether HSC clustering was intrinsic to aged HSCs. To this end, we transplanted 1,000 young and aged HSCs from AcRFP mice (that ubiquitously and constitutively express the red fluorescent protein [RFP]) into young *Rag2*−/−γ*c*^−/−^*Kit*^W/Wv^ recipient mice and imaged the bone marrow 6 weeks after transplant. We observed a higher frequency of HSC clusters (58% of all HSCs) in the bone marrow of recipients of aged HSCs compared to recipients of young HSCs (11% of all HSCs) (Fig 6F and 6G and 6J). Aged HSCs were also closer to each other and more frequently located in proximity of another HSC when compared to the distribution of young HSCs (Fig 6H and 6I). The frequency of clusters, compared to non-transplanted controls, increased for both aged and young donor HSCs (Fig 6J and S7E Fig), suggesting a contribution of the transplantation to aging of HSCs. In summary, clusters of HSCs in bone marrow are intrinsic to HSCs, are primarily formed by aged HSCs, and are consistent with an elevated frequency of symmetric divisions of aged HSCs.

### The transcriptome of paired daughter cells does not correlate with their potential

**Fig 7:**
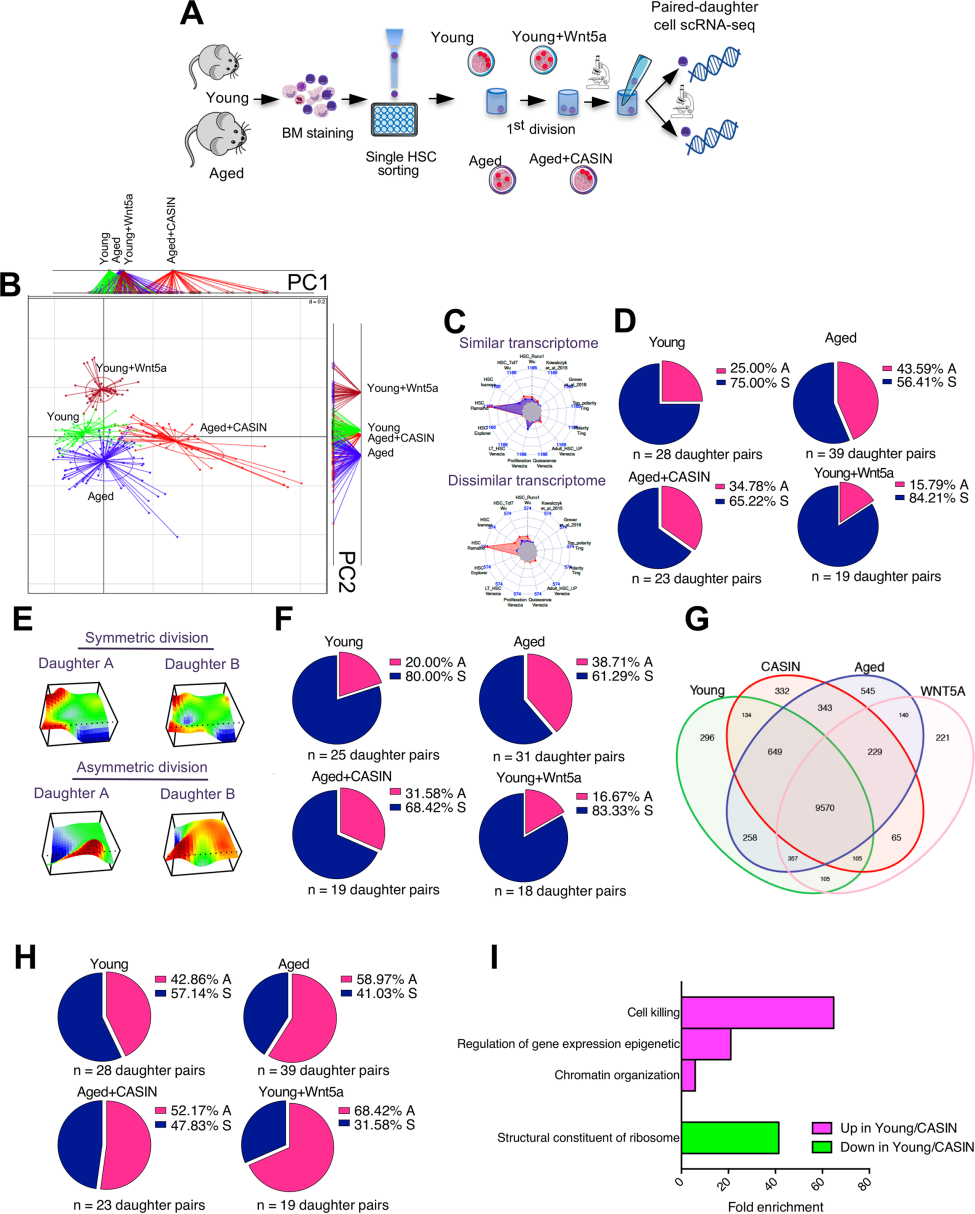
The transcriptome does not reflect the potential of daughter cells. (A) Schematic representation of the experimental setup for the preparation of scRNA-seq libraries of daughters pairs from young, aged, aged treated with CASIN, and young treated with Wnt5a HSCs (graphical sources: https://www.servier.de/medical-art and https://openclipart.org/) (B) Correspondence based on BGA of the global expression profile of single daughter cell pairs after division. In total, 25–28 pairs for young, 31–39 pairs for aged, 19–23 pairs for aged CASIN-treated, and 18–18 pairs for young Wnt5a treated HSCs; *n* = 3–5 biological repeats. (C) Representative radar plots of the GSEA showing 1 similar and 1 dissimilar daughter pair. The analysis approach simultaneously interrogated 13 previously published HSC and polarity signatures, and each daughter cell pair was depicted in a radar plot where vertices in the plot correspond to each of the considered gene set. Pairs were then compared for significance of similarity/dissimilarity by a goodness of fit test across all significant gene sets. (D) Pie charts depicting the percentage of asymmetric and symmetric divisions of young, aged, aged treated with CASIN, and young treated with Wnt5a daughter cell pairs based on the GSEA. (E) Representative 3D-SOM analysis of the whole scRNA-seq transcriptome of 1 concordant and 1 discordant daughter pair. (F) Pie charts depicting the percentage of asymmetric and symmetric divisions of young, aged, aged treated with CASIN, and young treated with Wnt5a daughter cell pairs based on the 3D-SOM metagene analysis. To quantify the degree of similarity between SOMs of daughter pairs, we identified genes that showed statistically significant log fold change compared to the mean global expression profile (namely metagenes, corresponding to the hills and valleys in the 3D plots shown in panel E). This information was then used to compare daughter pairs and identify concordant genes, i.e., genes that show same direction of change. Eventually, we compared the ratio of concordant genes to the discordant ones and calculated a QCR. Each pair was then identified as concordant or discordant based on the magnitude and direction of the QCR value (tested for significance using Monte-Carlo simulation). (G) Venn diagram showing differential gene expression analysis for up-regulated genes. (H) Pie charts depicting the percentage of asymmetric and symmetric divisions of young, aged, aged treated with CASIN, and young treated with Wnt5a daughter cell pairs based on the 134 genes up-regulated in the daughter cells from young and aged CASIN-treated arms but not in the aged HSCs and young Wnt5a-treated ones. (I) Bar graph showing fold enrichment for biological processes in the 134 up-regulated genes in young and aged CASIN-treated daughter pairs but not in the aged HSCs and young Wnt5a-treated ones, and in the 6 genes down-regulated in young and aged CASIN-treated daughter pairs but not in the aged HSCs and young Wnt5a-treated ones. GO analyses for biological processes were done with Panther version 11.0. The primary data the figure is based on are provided in GEO GSE116712. BGA, between-group analysis; CASIN, cdc42 activity specific inhibitor; cdc42, cell division control protein 42; GO, gene ontology; GSEA, gene set enrichment analysis; HSC, hematopoietic stem cell; QCR, Quadrant Count Ratio; scRNA-seq, single-cell RNA sequencing; SOM, self-organizing maps.

To investigate the extent of the correlation between the divisional asymmetry/symmetry of HSCs and the transcriptome of daughter cells, we performed single-cell RNA sequencing (scRNA-seq) on paired daughter cells (Fig 7A).

A global supervised “between-group analysis” (BGA) showed distinct clustering of daughter cells derived from the different experimental arms (Fig 7B; S4 Table; S8 Fig; S9A–S9C Fig). Daughter cells from aged and aged CASIN-treated HSCs showed a higher transcriptional heterogeneity compared to daughter cells from young and young treated-with-Wnt5a HSCs. When daughter pairs were separated based on chronological age, the aged CASIN-treated arm was closer to the young than to the aged control arm. However, the daughter cells from young Wnt5a-treated HSCs were also closer to the young control than to the older control arm (Fig 7B).

To evaluate asymmetry/symmetry based on the difference of the transcriptome between the daughter cells, we first performed Self-Organizing Maps (SOM) analyses followed by three different analytical approaches (S10 Fig). We selected statistically significant genes (*p*-value and q-value < 0.05) that fall into distinct clusters of highly correlated genes (metagenes) that, summarily, presented a portrait that captures the global transcriptional landscape of a given cell. Cells with similar portraits and significant sets of genes will implicitly have high levels of transcriptional similarity. In the first analysis, we performed a hypergeometric-model–based gene set enrichment analysis using 13 previously published HSC and polarity signatures [32–39] by simultaneously interrogating all the gene sets (Fig 7C; S11 Fig; S5 Table). When we then compared cells belonging to a pair, the majority of the pairs showed a high degree of similarity (concordance) across all arms (Fig 7D). In the second approach, we quantified the degree of similarity between SOMs of daughter pairs by comparing the genes that showed statistically significant log fold-change compared to the mean global expression profile (Fig 7E). Each pair was classified as concordant or discordant based on the magnitude of genes showing the same direction of change. Again, we observed a high level of concordance for all daughter pairs (Fig 7F).

Finally, taking into consideration the results of the transplants (Fig 4C) and the mathematical modeling (Fig 3E), we sought to confirm higher asymmetric outcomes (discordant transcriptome profiles) in young and aged CASIN-treated HSCs and increased symmetric division outcomes (concordant transcriptome profiles) in aged and young Wnt5a-treated HSCs. To this end, we analyzed differentially expressed genes among daughter cells from young and aged CASIN-treated HSCs to highlight discordant divisions among the different pairs (Fig 7G; S6 Table). Results showed a percentage of asymmetric divisions slightly higher for all arms compared to the previous gene set enrichment and fold change analysis approaches; however, again results were not in agreement with the transplants and the mathematical prediction, and no significant difference was observed among the four arms (Fig 7H).

In the end, we analysed enrichment for biological processes in the 134 up-regulated genes in young and aged CASIN-treated daughter pairs. We observed a significant enrichment in epigenetic regulation of gene expression and chromatin organization (Fig 7I). In the down-regulated gene sets, structural constituents of ribosomes were significantly enriched (Fig 7I; S9D Fig). No significant enrichment was found for the genes up-regulated in aged and young Wnt5a-treated cells (S7 Table). Therefore, the transcriptome in daughter cells to a large extent does not correlate with the function of a daughter cell as determined in the transplantation experiments.

### Open chromatin configuration in daughter cells correlates with daughter cell function

**Fig 8:**
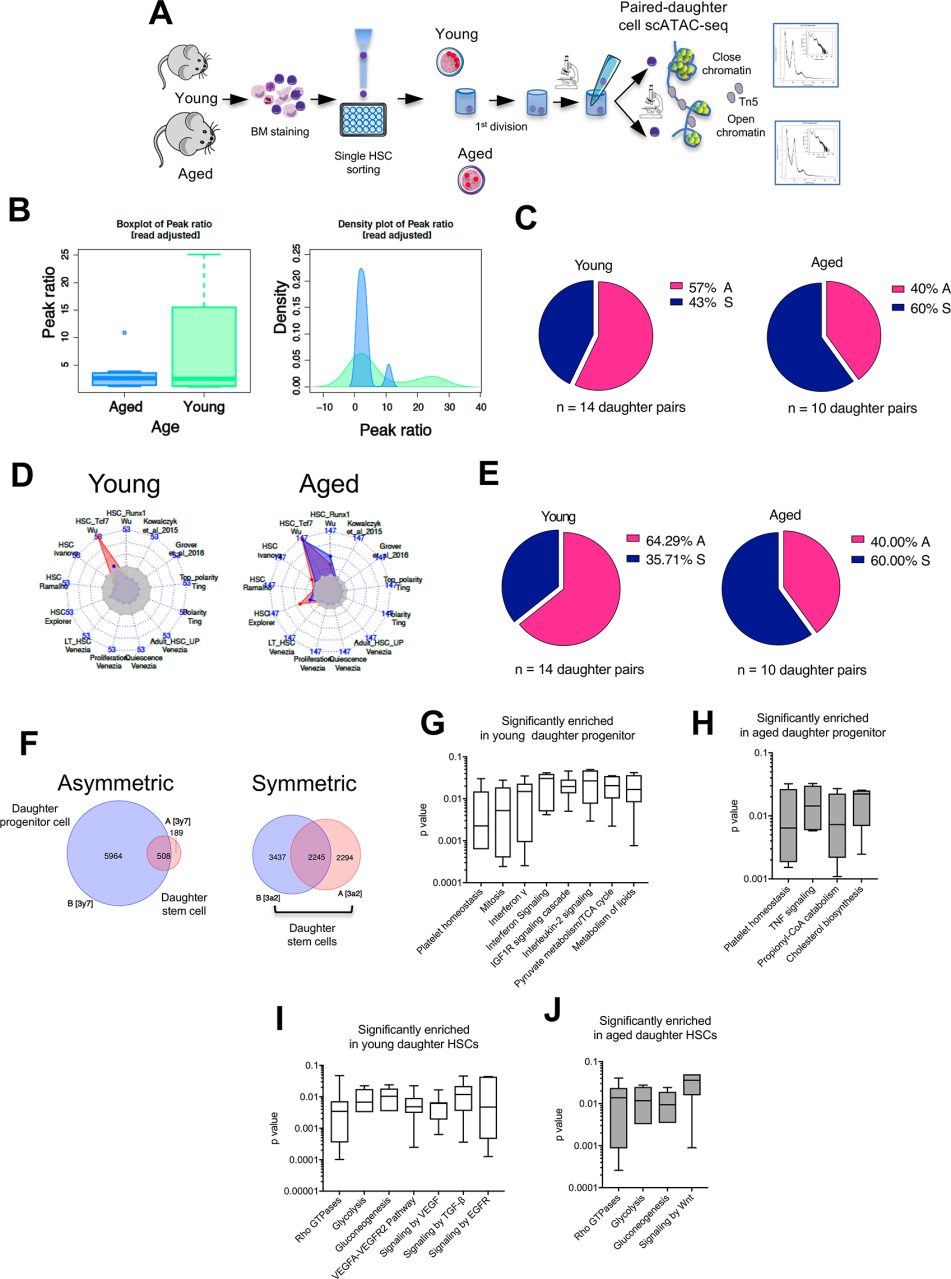
scATAC-seq on daughter cells shows asymmetric division for young HSCs, while aged HSCs undergo mainly symmetric divisions. (A) Schematic representation of the experimental setup for the preparation of scATAC-seq libraries from young and aged daughter cell pairs. Graphical sources: https://www.servier.de/medical-art and https://openclipart.org/. (B) Ratio of number of peaks of daughter pairs in young (green) and aged (blue) cells. For each pair, the ratios have been adjusted with the ratio of mapped reads in the pair (unadjusted ratios are shown in S11C Fig). A ratio of one represents an equal number of peaks in a given pair. The right panel represents the distribution of the ratio of peak counts. (C) Pie charts depicting the percentage of asymmetric and symmetric divisions of young and aged HSCs based on scATAC-seq analysis of daughter cells (see also S8 Table for statistics). For those pairs for which we have sufficient number of peaks for the reshuffling simulation test of the null hypothesis that cells in a pair show same peak counts in same genomic positions (column V in S8 Table), the asymmetry assignment is based on the significance of no overlap and on the ratio of peak counts (as a measure of global chromatin accessibility) greater than 2 (column P in S8 Table). (D) Radar plot based on 13 published “stemness” signatures. Signatures that show significant enrichment based on a hypergeometric test (adjusted *p* < 0.05) are shown by red and blue shades representing the 2 cells in a given pair. Signatures that do not extend beyond the shaded gray center are considered not significant (see S14 Fig. for the reference to each specific signature). (E) “Stemness” enrichment analysis-based pie charts showing the frequency of symmetric and asymmetric division of young and aged HSCs. A goodness-of-fit chi-squared test assessing the matches in significance between cells in a pair was used to assign cells either as symmetric or asymmetric. (F) Example of a Venn diagram depicting an asymmetric (left panel; significant difference in peak counts) and a symmetric (right panel; comparable peak counts) daughter cell pair from young HSCs based on the difference in peak counts (ratios) as a measure of the degree of chromatin accessibility. For symmetrically dividing HSCs, both daughter cells were considered daughter stem cells (supported by our sc-transplant data). For asymmetrically dividing HSCs, the daughter presenting with the lower amount of peak counts (lower degree of chromatin accessibility) was considered the daughter stem cell, while the other daughter cell was assigned to the daughter progenitor (i.e., not stem cell) group. (G, H) Daughter progenitor cells were subjected to Reactome GO analysis. The most frequently significantly enriched GOs among young (panel G) and aged (panel H) daughter progenitor cells are shown. (I, J) Daughter stem cells were subjected to Reactome GO analysis. The most frequently significantly enriched GOs among young (panel G) and aged (panel H) daughter stem cells are shown. The primary data the figure is based on are provided in GEO GSE116712. A, asymmetric; GO, gene ontology; HSC, hematopoietic stem cell; S, symmetric; scATAC-seq, single-cell transposase-accessible chromatin sequencing.

It is well established that genes specific for certain cell types or that encode crucial lineage determining regulators might be activated in a stepwise fashion at the levels of chromatin accessibility first, followed by transcriptional activation [40]. Therefore, to investigate whether chromatin accessibility might be distinct in daughter, cells we performed single-cell transposase-accessible chromatin sequencing (scATAC-seq) on daughter cells from young and aged HSCs (Fig 8A; S12 Fig; S13 Fig).

ATAC-seq was selected because it reflects alterations in chromatin accessibility that are possibly also linked to changes in the level of H4K16ac (see Fig 2A and [41–44]). The ATAC-seq profile of single daughter cells showed the expected distribution of insert sizes and an enrichment at transcription start sites (TSSs) of nucleosome-free fragments and the expected profile with respect to CCCTC-binding factor (CTCF) binding sites (S13 Fig). ATAC-seq peaks were annotated predominantly to TSSs, introns, and intergenic regions up to 1 kb apart from TSSs (S14A Fig). The overall scATAC-seq profile of daughter pairs of young HSCs showed a marked increase in the value of peak ratio compared to daughter pairs of aged HSCs. This resulted in a difference in ATAC-seq peak amounts between daughters from young HSCs, while daughter pairs of aged HSCs presented more often with having equal amounts of ATAC-seq peaks and a ratio quite uniform around 1 (Fig 8B). The difference in the ATAC-seq peak amount between paired daughters allowed for scoring of divisions as symmetric or asymmetric. Briefly, for those pairs for which we had a sufficient number of peaks for the reshuffling simulation test of the null hypothesis that cells in a pair show the same peak counts in the same genomic positions, asymmetry assignment was based on the significance of no overlap and the ratio of the peak counts (as a measure of global chromatin accessibility) greater than 2 (S8 Table and S15 Fig). Based on these statistical parameters, young HSCs presented with 57% of asymmetric divisions, while aged HSCs showed 40% of asymmetric divisions (Fig 8C; S8 Table; S15 Fig). The symmetry/asymmetry difference between young and aged cells determined by 3D-IF for Cdc42 and H4K16ac, in transplantation assays and as predicted by the mathematical model, is thus similar to the difference in the ATAC-seq peak profile of paired daughters of young and aged HSCs.

We next annotated scATAC-seq peaks to genes. Similar to the analyses performed for the scRNA-seq data, we used gene set enrichment analysis to align daughter pairs to known stem cell signatures (Fig 8D and S16 Fig) and presented them again in radar plots. The data revealed also for these analyses a higher frequency of asymmetric divisions for young HSCs (64%) compared to asymmetric division for aged HSCs (40%) (Fig 8E and S16 Fig). Individual daughter cells from young and aged pairs were subsequently grouped according to the mode of division as established based on the ATAC-seq peak ratio (S8 Table and Fig 8B and 8C): both daughter cells of all symmetric divisions were assorted as stem cells because the majority of daughter cells stemming from symmetric divisions were, according to the transplantation assay, stem cells. As for asymmetric divisions, the daughter cells with the highest ATAC-seq peak number were assigned to the daughter progenitor cells group because daughter cells of symmetric divisions, which result in daughter stem cells, presented with a quite uniform and low peak count (Fig 8F). Next, transcription factor (TF) footprint analyses on ATAC-seq peaks identified significantly enriched TF binding sites common to daughter stem cells of both young and aged HSCs (S14B and S14C Fig and S17 Fig and S9 Table). Among them were TFs like NFY, SpiB, Sp5, Sp1, Runx, ERG, PU.1, and Maz (S14B–S14D Fig). Of these, the frequencies of target sequences with a Sp5, Sp1, Maz, and ERG motif were even further increased in daughter stem cells of aged HSCs (S14D Fig). In daughter progenitor cells from both young and aged HSCs, the enriched TFs set was very distinct from the one enriched in stem cells (S14E and S14F Fig), and factors like c-Myc, Usf2, and Stat3 further showed a decline in enrichment in aged daughter progenitors, while PU.1:IRF8, CEBP, CEBP:AP1, and Nanog showed the opposite trend (S14E Fig). However, only the frequency of target sequences for the E2F6 motif was significantly more enriched in aged daughter progenitors (S14G Fig).

Finally, gene ontology (GO) analysis revealed 603 and 490 significantly enriched pathways in young and aged daughter stem cells and 474 and 360 pathways in young and aged daughter progenitor cells (S18 Fig and S10 Table). More than 75% of young daughter progenitor cells shared enrichment for only 1.47% of all significantly enriched GOs. These daughter-progenitor–specific enriched GOs for young HSCs were platelet homeostasis, mitosis, interferonγ and interferon signalling, IL-2 and IGF1 signalling, and pyruvate and lipid metabolism (Fig 8G) [45–49]. In daughter progenitors of aged HSCs, 100% of the cells were enriched for only 2.77% of the GOs, which included platelet homeostasis, TNF signalling, propionyl-CoA catabolism, and cholesterol biosynthesis (Fig 8H) [50–52]. To further dissect differences between young and aged daughter progenitor cells, we performed gene set enrichment analyses for a previously reported multipotent hematopoietic progenitor cell signature [53]. Despite only 2 aged and 3 young daughter progenitor cells showing significant enrichment, both young and aged daughter progenitor cells showed a similar set of genes overlapping across cells in a given age group but also across the two age groups, indicating a similar pattern of chromatin accessibility in and around these genes in both young and aged daughter progenitor cells (S14H–S14I Fig).

Most of the daughter stem cells from both young and aged HSCs were enriched for GO pathways linked to glycolysis and gluconeogenesis and also for signaling linked to RhoGTPases, implying an important role of regulation of the glycolytic metabolism and of small RhoGTPase signaling (like Cdc42) for the regulation of stem-ness of daughter stem cells (Fig 8I and 8J) [54–56], as also predicted by our mathematical model (Fig 3). Similarly informative might be the finding that major differences in GO enrichment among daughter stem cells from young and aged HSCs were linked to signalling pathways involved in the interaction of HSCs with the stem cell niche: while daughter stem cells from young HSCs are enriched for GOs associated with TGFβ, VEGFR2, and EGFR signalling, daughter stem cells from aged HSCs are enriched for Wnt signalling (Fig 8I–8J) [15, 56–58]. In summary, open chromatin configuration and thus the epigenetic make-up of a daughter cell strongly correlates with the potential of the daughter cell.

## Discussion

The asymmetric or symmetric sorting of epigenetic information upon HSC division has been proposed as a way of controlling the potential of nascent daughter cells [9]. Here, we demonstrate, using a comprehensive set of paired daughter analyses that include single-cell 3D confocal imaging, single-cell transplants, scRNA-seq, and scATAC-seq, that the mode of HSC division is strongly linked to the polarity status before mitosis, which is in turn determined by the level of the activity Cdc42 in stem cells. These results consequently imply that the level of activity of Cdc42 in mother HSCs [4, 15] regulates the mode (symmetric/asymmetric) of HSC divisions, which results in a distinct allocation of epigenetic regulators like H4K16ac as well as of Cdc42 itself to daughter cells. Our data, combined with our mathematical model on the mode of divisions and the role of Cdc42 in daughter cells, further support that polar HSCs (that are the majority in young animals) undergo asymmetric divisions while apolar HSCs (that are the majority in aged animals) undergo primarily symmetric divisions. Aging and rejuvenation of HSCs, as a consequence of changes in the activity of Cdc42 in HSCs [15, 16], also imply a change in the mode of HSC divisions and thus link the mode of division of HSCs to changes in the function of daughter stem cells. In addition, our data (both experimental and mathematical modeling) strongly support that symmetric divisions, which are the primary mode of division of aged HSCs, might be directly linked to the increase in the number of HSCs in bone marrow upon aging. A striking new feature, clustering of HSCs within the bone marrow of aged animals (Fig 6), is most likely a consequence of the elevated frequency of symmetric divisions of aged HSCs. An interesting novel concept derived from HSC clustering is that, in such clusters, HSCs might serve as niche cells for other HSCs.

Recently published data support a dominant role of hematopoietic progenitor cells but not HSCs in maintaining long-term hematopoiesis in an unperturbed setting in young animals [59, 60]. Our findings are consistent with this novel concept because young HSCs will divide primarily asymmetrically and provide the progenitor cells that support hematopoiesis. Our data might predict that, upon aging, this dominant role of progenitor cells in driving hematopoiesis will shift towards a more relevant contribution of stem cells to hematopoiesis due to the reduced number of progenitors generated from aged HSCs.

The final fate of daughter cells after HSC divisions might be defined by signals intrinsic to stem cells that are allocated during division to daughter cells but might also be driven by extrinsic signals from the environment acting on daughter cells after mitosis is completed. The stem cell intrinsic signals need to be, by definition, epigenetic in nature (not linked to the DNA sequence), as daughter cells present with a genome that is identical to their mother cell. Our data and our mathematical model support that the decision on the potential of daughter cells is driven, to a large extent, by the mode of the division of the mother HSC, which is decided upon in the mother HSCs. We show distinct allocation of a polarity protein (Cdc42) and an epigenetic mark (H4K16ac) to daughter cells, which correlates with the potential of the daughter cells themselves. Our data also demonstrate that the overall transcriptome in daughter cells does not correlate with their potential, while the amount and the location of open chromatin regions is tightly linked to it. Furthermore, mathematical modelling and our ATAC-seq-linked GO signatures in daughter stem cells predict an important role for RhoGTPase signaling as well as metabolic signaling for the maintenance of the stem cell potential in daughter stem cells upon stem cell divisions. While a role of glycolysis in stem cell maintenance has been already described by multiple laboratories [54, 55], an important role for RhoGTPase signaling for stem cell maintenance has only been recently suggested [61, 62]. The daughter progenitor cell signature is distinct from the stem cell signature, while sharing for both young and aged daughter-cell gene loci linked to platelet homeostasis. It has been described that HSCs can rapidly differentiate into platelets [63]. In addition, progenitor cells display enrichment for signatures linked to interferon signaling, which has been, in the past, primarily associated with stem cell quiescence/activation [45, 64, 65].

Recently, it has been reported that H4K16ac can directly control chromatin accessibility [44], so it is likely that a symmetric or asymmetric distribution of H4K16ac to daughter cells (due to polarity/apolarity of the mark in mother stem cells) might contribute to distinct levels of chromatin accessibility and thus affect the fate of daughter cells. These findings are consistent with recent data supporting that epigenetic memory in HSCs is persistent and guides cellular function [66]. The data presented here also imply that an open chromatin configuration precedes changes in gene expression in daughter cells. Our ATAC-seq analyses imply that young HSCs might also establish a specific chromatin configuration that allow stem-cell–extrinsic signalling (TGFβ, VEGF, IGF1, and EGF signalling) to act on nascent daughter stem cells, while aged daughter stem cells present with open chromatin regions linked to Wnt signalling. A role for all of these factors for the biology of young and aged HSCs has already been described, further supporting these conclusions [15, 56–58].

In summary, our data imply an important role for Cdc42 activity/polarity in HSCs for driving the symmetric/asymmetric mode of division as well as a role of epigenetic mechanisms for determining the potential of daughter cells. The frequency of polar HSCs decreases upon aging, which results in more symmetric divisions but daughter stem cells with impaired potential.

## Materials and methods

### Ethics statement

This study involves animal research (mice, C57BL/6 strain). All mice were housed in the animal barrier facility under pathogen-free conditions either at the University of Ulm or at CCHMC. All mouse experiments were performed in compliance with the German Law for Welfare of Laboratory Animals and were approved by the Institutional Review Board of the University of Ulm or by the IACUC of CCHMC (approval number: TVA 0.1172 and IACUC2013-0154). This study does not involve human participants and/or tissues, and it does not involve collection of plant, animal, or other materials from a natural setting.

### Mice

C57BL/6 mice (10- to 12-week-old) were obtained from Janvier. Aged C57BL/6 mice (20- to 26-month-old) were obtained from the internal divisional stock (derived from mice obtained from both The Jackson Laboratory and Janvier) as well as from NIA/Charles River. Congenic young and aged C57BL/6.SJL-*Ptprc*^*a*^/Boy (BoyJ) mice were obtained from Charles River Laboratories or from the internal divisional stock (derived from mice obtained from Charles River Laboratories). Pan-RFP mice carrying constitutively active ROSA26-tdRFP alleles (indicated in the manuscript as Ac-RFP mice) were obtained from Professor Hans Joerg Fehling (Institute of Immunology, Ulm University) and were previously generated by intercrossing C57BL/6-Gt(ROSA)26Sor^tm1Hjf^/Ieg mice [67] with animals from a germline Cre-deleter strain [68]. Offspring in which the ROSA26-driven fluorescent tdRFP reporter had been activated irreversibly as the result of loxP/Cre-mediated recombination in the germline were backcrossed for >10 generations onto C57BL/6, thereby eliminating the Cre recombinase transgene. Ac-RFP mice were used as homozygotes. Rag2−/−γc^−/−^Kit^W/Wv^ mice were obtained from the internal divisional stock (derived from mice obtained from Hans-Reimer Rodewald [29]). All mice were housed in the animal barrier facility under pathogen-free conditions either at the University of Ulm or at CCHMC. All mouse experiments were performed in compliance with the German Law for Welfare of Laboratory Animals and were approved by the Institutional Review Board of the University of Ulm or by the IACUC of CCHMC.

### Single-cell transcriptome profiling

SMART-Seq version 4 Ultra Low Input RNA kit from Clontech (catalog number 634892) was used. The kit generates Illumina compatible RNA-Seq libraries. Sorted single cells were cultured with and without treatment in the presence of cytokines until first cell division (40–44 hours). The daughter cells were manually separated, washed with phosphate-buffered saline (PBS), and collected for RNA sequencing. The cDNA synthesis and amplification was done as recommended by Clontech. The amplified cDNA generated from single cells were used to make libraries using Illumina’s Nextera XT DNA Library Preparation kit (catalog number FC-131-1096) as per Illumina’s instructions. The generated libraries were quantified using an agilent bio-analyzer, pooled, and subjected to next-generation sequencing in a Hi-Seq 2500 for pair-end 75 bp sequencing condition.

### Single HSC sorting and culturing

Bone marrow mononuclear cells were flushed out from long bones (tibiae and femurs) of young and aged mice and were isolated by low-density centrifugation (Histopaque 1083, Sigma). Low-density bone marrow cells were stained with a cocktail of biotinylated lineage antibodies. Biotinylated antibodies used for lineage staining were all rat anti-mouse antibodies: anti-CD11b (clone M1/70), anti-B220 (clone RA3-6B2), anti-CD5 (clone 53–7.3), anti-Gr-1 (clone RB6-8C5), anti-Ter119, and anti-CD8a (clone 53–6.7) (all from eBioscience). After lineage depletion by magnetic separation (Dynalbeads, Invitrogen), cells were stained with anti-Sca-1 (clone D7; eBioscience), anti-c-kit (clone 2B8; eBioscience), anti-CD34 (clone RAM34; eBioscience), anti-CD127 (clone A7R34; eBioscience), anti-Flk-2 (clone A2F10; eBioscience), and Streptavidin (eBioscience). Single long-term HSCs (gated as LSK CD34^−^Flk2^−^) [15] were sorted using a BD FACS Aria III (BD Bioscience) into Terasaki plates. HSCs were cultured in IMDM plus 10% FBS plus P/S plus 100 ng/mL TPO, G-CSF, and SCF (PeproTech) at 37°C, 5% CO_2_, 3% O_2_. CASIN was used at a dose of 5 μM. Wnt5a (R&D) was used at a dose of 100 ng/mL. Each well of the Terasaki plate was microscopically tracked, and within 1 hour from HSC division, daughter cells were separated by mechanical pipetting, deposited in distinct Terasaki wells, and controlled again by microscopy. Eventually, the daughter pair was injected into recipient mice (1 daughter in 1 mouse, the second daughter into a littermate).

### Single daughter cell transplant

For daughter cell pair transplantation, young (2- to 4-month-old) and aged (24-month-old) C57BL/6.SJL-*Ptprc*^*a*^/Boy (BoyJ) (Ly5.1^+^) mice were used as donors. Single daughter cell HSCs were separated by pipetting under microscopy control and were directly injected into the retro-orbital vein of recipient mice. PB chimerism was determined by FACS analysis at week 4, 12, 16, and 24 post transplant. The transplantation experiment was performed 8 times with a cohort of 10 to 16 recipient mice per group each transplant. In total, 110 recipients were injected, and 94 mice showed detectable chimerism (contribution to PB >0.1%) at a minimum of 1 time point of the analyses.

### Flow cytometry

PB immunostaining was performed according to standard procedures, and samples were analyzed on a LSRII flow cytometer (BD Biosciences). Monoclonal antibodies to Ly5.2 (clone 104, eBioscience) and Ly5.1 (clone A20, eBioscience) were used to distinguish recipient from donor cells. For PB lineage analysis, the antibodies used were all from eBioscience: anti-CD3ε (clone 145-2C11), anti-B220 (clone RA3-6B2), anti-Mac-1 (clone M1/70), and anti-Gr-1 (clone RC57BL/6-8C5). The engraftment is plotted as percentage of donor-derived cells among total white blood cells. B cells, T cells, and myeloid cell lineage data are plotted as the percentage of B220^+^, CD3^+^, and Myeloid (Gr-1^+^, Mac-1^+^, and Gr-1^+^Mac-1^+^) cells among donor-derived cells. Please note that data are plotted in a logarithmic scale to accommodate sample heterogeneity.

### IF staining

Freshly sorted HSCs were seeded on fibronectin-coated glass coverslips. At 32 to 34 hours after culturing in IMDM plus 10% FBS plus P/S plus 100 ng/mL TPO, G-CSF, and SCF (PeproTech) ± CASIN 5 μM ± Wnt5a (R&D) 100 ng/mL at 37°C, 5% CO_2_, 3% O_2_, cells were fixed with BD Cytofix Fixation Buffer (BD Biosciences) [15]. After fixation, cells were gently washed with PBS, permeabilized with 0.2% Triton X-100 (Sigma) in PBS for 20 minutes, and blocked with 10% Donkey Serum (Sigma) for 30 minutes [15]. Primary and secondary antibody incubations were performed for 1 hour at room temperature. The primary antibodies were anti-alpha tubulin antibody (Abcam, rat monoclonal ab6160), anti-Cdc42, and anti-H4K16ac obtained from Millipore and Abcam (we tested 2 different antibodies for each target, and results were consistent; all 4 antibodies were rabbit polyclonal; anti-Cdc42 from Millipore was previously validated [4]). The secondary antibodies for IF were anti-rat DyLight488-conjugated antibody, anti-rat DyLight647-conjugated antibody, and anti-rabbit DyLight549-conjugated antibody (all obtained from Jackson ImmunoResearch). Coverslips were mounted with ProLong Gold Antifade Reagent with or without DAPI (Invitrogen, Molecular Probes). Samples were imaged with an AxioObserver Z1 microscope (Zeiss) equipped with a 63× PH objective. Images were analyzed with Zen software. Alternatively, samples were analyzed with an LSM710 confocal microscope (Zeiss) equipped with a 63X objective. Primary raw data were imported into the Volocity Software package (version 6.2, Perkin Elmer) for further processing and conversion into 3D images.

### Whole-mount IF staining

After intravenous injection of APC-anti-CD31 (clone MEC13.3, BioLegend) and Alexa Fluor 647-anti-CD144 (clone BV13, BioLegend) antibodies, long bones were harvested after postmortem heart perfusion with 4% paraformaldehyde (PFA) in PBS and were post-fixed in 4% PFA/PBS solution for 24 hours at 4°C. Subsequently, bones were embedded without bisecting in optimum cutting temperature (OCT) compound (Tissue-Tek) and were snap frozen in liquid nitrogen and stored at −80°C. Bones were shaved along the longitudinal axis on a cryostat until the BM cavity was exposed (S5A Fig). The bones were purified from melting OCT. Specimens were fixed again in 4% PFA/PBS at RT for 30 minutes. Tissues were blocked and permeabilized with buffer containing 20% donkey serum and 0.5% Triton X-100, were incubated with a fluorescent-labeled antibody PE-anti-CD150 or AlexaFluor488-anti-CD150 (clone TC15-12F12.2, BioLegend) as well as Biotin-labeled primary antibodies anti-CD41 (clone MWReg30), anti-CD48 (clone HM48-1), anti-CD11b (clone M1/70), anti-B220 (clone RA3-6B2), anti-CD5 (clone 53–7.3), anti-Gr-1 (clone RB6-8C5), anti-Ter119, and anti-CD8a (Clone 53–6.7) (all from eBioscience) 1 to 3 days at 4°C and stained with Streptavidin-eFluor450 (eBioscience) or Streptavidin- FITC (eBioscience) for 2 hours at RT. The fluorescently labeled bone tissues were placed cut-face down onto a 4-well-μ-Slide and were covered in antifade to prevent tissue desiccation. The preparations were immediately examined under Zeiss LSM 710 or Leica TCS SP8 confocal microscopes and analyzed with the image analysis software Volocity (version 6.2; Perkin Elmer).

### scRNA-seq data analysis

Raw Fastq files were adapter trimmed using Trimm Galore (Babraham Institute), a wrapper tool around Cutadapt and FastQC. Reads with Phred score of 20 or higher were kept for further analysis. Reads were aligned to the mouse reference genome version 10 (GRCm38/mm10) using tophat [69]. Transcript abundance estimation, fragments per kilobase of exon per million fragments mapped (FPKM) calculations, and normalization was done using Cufflinks [70–73], a package of tools for transcriptome assembly and differential expression analysis of RNA-Seq data. Correspondence-based BGA was done using R and bioconductor package made4 [74, 75]. SOM and metagene analysis was performed using oposSOM [76]. Calculation of concordant and discordant daughter pairs was done using quadrant count ratio (QCR) by counting genes that fall in the 4 quadrants. A chi-squared test was used to test the significance of difference of concordant (quadrants I and III) and discordant genes (quadrants II and IV). Gene set enrichment analysis was carried out using stem-ness and cell-polarity–related gene sets. The analysis approach simultaneously interrogated 13 previously published HSC and polarity signatures [32–39], and each daughter cell pair was depicted in a radar plot such that vertices in the plot correspond to each of the considered gene sets: Mm_HSC_Runx1_Wu [32], Mm_HSC_Tcf7_Wu[32], Mm_LT_HSC_Venezia [36], Mm_Proliferation_Venezia [36], Mm_Quiescence_Venezia [36], Polarity_factors_Ting [37], Novel_HSC_regul_polar_Ting [37], Grover et al, 2016 [38] and Kowalczyk et al, 2015 [39] (S6 Table). Cells were first individually analyzed, and significance of gene set enrichment was tested using hypergeometric test. Concordance between daughter pair was then visualized using radar plots and tested using goodness-of-fit test. Knowledge-driven comparison between young (young LT-HSC/CASIN-treated aged LT-HSC cells) versus aged (aged LT-HSC/WNT5A-treated young LT-HSC) arms was made by populating genes that show same direction of significant change (*p*-value cutoff 0.05) in expression in a given arm but not in the other. Genes fulfilling this criterion were used to generate the Venn diagram shown in Fig 4G. Daughter pair concordance and discordance in each treatment group was determined based on the level of significance of correlation that each pair shows. GO analysis and analysis of enrichment for biological processes was done according to standard GO Consortium dataset by using PANTHER version 11 [77].

### Assay for ATAC-seq

The protocol was adjusted from previously published procedures [78]. Briefly, single HSCs from young and aged C57BL/6 mice were sorted into Terasaki plates with IMDM supplemented with 10% FBS, P/S, and 100 ng/mL of SCF, G-CSF, and TPO at 3% O_2_, 5% CO_2_. Cells were tracked microscopically, and after the first division, the daughter cells were separated by pipetting and singularly subjected to fragmentation of open chromatin regions using Tn5 transposase (Illumina), followed by a pre-amplification step, library preparation, and subsequent paired-end sequencing. For the pre-amplification, NEBNext Ultra II Q5 Master Mix was used with Primer 1: 5’GTCTCGTGGGCTCGGAGATGTGTATAAGAGACAG3’ and Primer 2: 5’TCGTCGGCAGCGTCAGATGTGTATAAGAGACAG3’. For dual-indexing, 10 μL of the pre-amplified ATAC reaction was used as input for Nextera index kit (Illumina). The generated libraries were quantified using an agilent bio-analyzer and a qPCR kit (New England Biolabs), pooled, and subjected to next-generation sequencing in a Hi-Seq 3000 for paired-end 75 bp sequencing condition.

### ATAC-seq data analyses

We used the ENCODE guidelines and current standards of ATAC-seq data analysis. This was achieved by using a combination of ENCODE prototype scripts and in-house algorithms. Briefly, sequences were aligned to the UCSC mouse reference genome mm10 using Bowtie 2 [79]. Peak calling was performed using MACS [80]. Additional analysis, visualization, and DNA footprint analyses were done using various in-house R scripts and published Bioconductor packages (ATACSeqQC) [81]. Overlap analysis between peak files was done using bedtools [82]. Random chromosome-controlled reshuffling of peaks was performed using bedtools followed by Monte Carlo Simulation based on in-house script. TF motif analysis and annotation were done using HOMER [83]. Gene set enrichment analysis was carried out using stemness and cell-polarity–related gene sets. The analysis approach simultaneously interrogated 13 previously published HSC and polarity signatures [32–39], and each daughter cell pair was depicted in a radar plot in which vertices in the plot correspond to each of the considered gene sets: Mm_HSC_Runx1_Wu [32], Mm_HSC_Tcf7_Wu [32], Mm_LT_HSC_Venezia[36], Mm_Proliferation_Venezia [36], Mm_Quiescence_Venezia [36], Polarity_factors_Ting [37], Novel_HSC_regul_polar_Ting [37], Grover et al, 2016 [38], and Kowalczyk et al, 2015 [39] (S6 Table). Cells were first individually analyzed, and significance of gene set enrichment was tested using hypergeometric test. Concordance between daughter pair was then visualized using radar plots and tested using goodness-of-fit test.

### Statistical analyses [15]

Data were tested to meet normal distribution. The variance was similar between groups that were statistically compared. All data are plotted as mean plus 1 SEM unless otherwise stated. The SEM is used to indicate the precision of an estimated mean. Such a data representation does not affect the statistical analyses as variance information is utilized in the test statistics. A paired Student *t* test was used to determine the significance of the difference between means of 2 groups. One-way ANOVA or two-way ANOVA were used to compare means among 3 or more independent groups. Bonferroni post-test to compare all pairs of dataset was determined when overall *p*-value was < 0.05. All statistical analyses were determined with Prism 4.0 c version. In order to choose sample size, we used GraphPad StatMate Software version 2.0b, estimating an SD between 2 and 8 (depending on the experiment and the possibility of increasing sample size). For transplantation experiments, mice showing signs of sickness and with clear alterations of blood parameter and/or showing signs of major disease involving also nonhematopoietic tissues were excluded from analysis. As for in vitro experiments, samples were excluded from analysis in the case of clear technical problems (error in immune-blotting or staining procedures or technical problems with reagents). All criteria for exclusions of samples from in vivo or in vitro experiments were preestablished. Each figure legend contains the number (*n*) of biological repeats (samples obtained from experiments repeated on different days and starting from different mice) of the statistical analysis. Mice for experiments were randomly chosen from our in-house colonies or from suppliers. All mice were C57BL/s6 females unless otherwise stated. The investigator was not blinded to the mouse group allocation nor when assessing the outcome (Rag2−/−γc^−/−^Kit^W/Wv^ mice, aged mice, or young mice transplanted with aged BM stem cells require particular care and follow-up).

## Supporting information

S1 Data Individual numerical values that underlie data displayed in Fig 1 to Fig 6 and in S1 Fig to S7 Fig. (XLSX)

S1 Text Mathematical modeling of aging-related changes in HSC polarity and the symmetry of divisions. (DOCX)

S1 Fig Mode of division: Young/polar HSCs divide asymmetrically, while aged/apolar HSCs undergo symmetric divisions. (A) Schematic representation of the possible mode and outcome of polar and apolar HSC divisions. The establishment of cell polarity within the mother HSC is supposed to be one fundamental mechanism that controls mode and outcome of asymmetric stem cell division. Such stem cell asymmetric division allows one daughter cell to become differentiated and the other to retain stem cell potential; in contrast, symmetric division allows both daughter cells to adopt equivalent fates. (B) Percentages of HSCs in metaphase, anaphase, or telophase among total mitotic HSCs. (C–J) Quantification of the amount of Cdc42 and H4K16ac in nascent daughter cells. Each pair is connected by a line. *p < 0.05, **p < 0.01, ***p < 0.001; *n* = 2–3 biological repeats; 25 pairs for young, 26 pairs for aged, 26 pairs for aged + CASIN and 14 pairs for young + Wnt5a for panel C–F; 41 pairs for young, 37 pairs for aged, 40 pairs for aged + CASIN, and 26 pairs for young + Wnt5a for panel G–J. (K) Representative epifluorescence pictures of young and aged HSCs cultured with and without growth factors (GF; SCF, G-CSF, and TPO all 100 ng/mL). Panels show DAPI (nucleus, blue), Cdc42 (red), and tubulin (green). The column graph depicts the percentage of polar cells retrieved in each sample. *n* = 3 biological repeats. Culture conditions did not affect the percentage of polar HSCs in both young and aged cell preparations. (L) Representative epifluorescence pictures of dividing (metaphase) young, aged, aged treated with CASIN 5 μM, and young treated with Wnt5a 100 ng/mL HSCs. Panels show DAPI (nucleus, blue), Cdc42 (red), and tubulin (green). The dashed lines cross transversally the dividing cells touching both opposite poles. The fluorescence intensity was measured along the dashed line (panel M); representative 3D confocal reconstruction of HSCs stained during division. The images show tubulin (green), H4K16ac (magenta), and the nucleus (DAPI, blue). The total level of H4K16ac during all phases of mitosis (metaphase, anaphase, and telophase) remained stable.
(TIF)

S2 Fig 3D-IF reconstruction of the distribution of Cdc42 and H4K16ac in all dividing cells detected and analyzed. (PDF)

S3 Fig Details of the mathematical modeling approach. (A) Sketch of the ODE model describing intracellular dynamics. Total Cdc42 is assumed to be autoregulative while an age-dependent proportion is activated. Active Cdc42 inhibits the cell’s acetylation level. (B) The variance of Cdc42 distribution (as a measure of apolarity) increases with increasing Cdc42 activity. (C) Representation of a polar and an apolar cell, respectively, in terms of a normal distribution *N*(*μ*,*σ*^2^) of Cdc42 on the cell’s circular outline (and AcH4K16 within the cell nucleus opposite Cdc42 localization). The x-axis of the histogram represents the circle line ranging periodically from –*π* to *π*. Polar cells concentrate Cdc42 at a pole around zero while AcH4K16 is distributed opposite. In contrast, apolar cells show a rather uniform protein distribution due to an elevated variance *σ*^2^. (D) Cell division for a homogeneous polar cell population. All cells of the population show a similar polarity pattern and are divided by an arbitrary plane through their center. This division event leads to asymmetric cell divisions almost exclusively (dark green in upper pie chart) and, after running the cell-intrinsic process (ODE model), ends up with a close to 1:1 ratio of LT-HSCs (white) and progenitors (yellow). (E) Cell division for a homogeneous apolar cell population ends up with symmetric divisions and LT-HSC daughters only. (F–G) Cell division simulation for a population of 1,000 young and aged LT-HSCs, respectively, being heterogeneous with respect to polarity. Pie charts (correspond to simulation pie charts in Fig 3E) depict the ratio of asymmetric and symmetric cell divisions and the ratio of LT-HSCs and progenitors, respectively. The histograms depict the frequency of daughter cells with a certain Cdc42 concentration after cell division. Upper histograms relate the symmetry of division with the specific Cdc42 concentration in the daughters. Lower histograms relate the results of the intracellular ODE model with total Cdc42 concentration after cell division. Cells with a Cdc42 concentration below 2 end up in a Cdc42-low state (= progenitors), whereas the other daughters end up in a Cdc42-high state (= LT-HSCs). (H) Distribution of *k*_+_ values (phosphorylation constant of Cdc42) for heterogeneous populations of young and aged LT-HSCs, respectively. (I) Both young and aged LT-HSCs present with a 2-fold increase of Cdc42 fluorescence intensity compared to progenitor cells as quantified by intracellular flow cytometry.
(TIF)

S4 Fig Symmetric and asymmetric HSC divisions: in vivo read-out in transplants. (A) Representative flow cytometry charts of PB from two mice 24 weeks post transplantion of individual but paired daughter cells (named arbitrarily A and B) that originated from a distinct young mother HSC. Donor-derived contribution to PB is higher than 0.1% of total WBC, and contribution is detected for both lymphoid (B cells, B220+ and T cells, Cd3+) and myeloid lineages (Gr1+, Mac1+, and Gr1+Mac1+cells). Consequently, the division of the mother HSC was scored as symmetric. (B) Representative flow cytometry charts of PB from two mice 24 weeks post transplantation of individual but paired daughter cells (named arbitrarily A and B) that originated from a distinct aged mother HSC. Donor-derived contribution to PB is higher than 0.1% of total WBC and the contribution is detected for both lymphoid (B cells, B220+ and T cells, Cd3+) and myeloid lineages (Gr1+, Mac1+, and Gr1+Mac1+cells). Consequently, the division of the mother HSCs was scored as symmetric. (C) Representative flow cytometry charts of PB from two mice 24 weeks post transplantation of individual but paired daughter cells (named arbitrarily A and B) that originated from a distinct young mother HSC. Donor-derived contribution to PB is higher than 0.1% of total WBC, but the contribution is detected for both lymphoid (B cells, B220+ and T cells, Cd3+) and myeloid lineages (Gr1+, Mac1+, and Gr1+Mac1+cells) only for daughter cell A. Daughter cell B shows no contribution to lymphoid cells (Cd3+ and B220+ cells). Consequently, the division of the mother HSCs was scored as asymmetric. (D) Representative flow cytometry charts of PB from two mice 24 weeks post transplantation of individual but paired daughter cells (named arbitrarily A and B) that originated from a distinct young aged HSC. Donor-derived contribution to PB is higher than 0.1% of total WBC only in daughter cell A. Daughter cell B shows a contribution to PB that is lower than 0.1% of total WBC. Consequently, the division of the mother HSCs was scored as asymmetric.
(TIF)

S5 Fig Daughter progenitor cells in the transplantation setting. (A) Kinetics of the chimerism of the overall level of engraftment of donor-derived cells and of each donor-derived lineage (B cells, T cells, and myeloid cells). Shown are young, aged, aged treated with CASIN, and young treated with Wnt5a daughter cells that were retrospectively identified as daughter progenitor cells. (B–C) Percentage of donor-derived cells at 4 weeks and 24 weeks after transplantations in PB of mice transplanted with single (retrospectively identified) daughter progenitor cells. *n* = 9 for young, *n* = 5 for aged, *n* = 7 for aged + CASIN, and *n* = 1 for young + Wnt5a. (D) Engraftment and lineage contribution for each single-cell transplant analysed. Shown is the final time point (24 weeks). Each daughter pair is identified by a number and A/B. All underlying data for this figure can be found in S1_Data panels 5A and S5B (including data for S5A, S5C and S5D Fig). A, aged; C, aged + CASIN; W, young + Wnt5a; Y, young.
(TIF)

S6 Fig Frequency of true HSCs among mother cells based on reconstitution. (A) Pie charts depicting the frequency of mother cells that generated at least one daughter stem cell. Since upon division they generated at least one daughter stem cell, the mother cells were scored as “true” HSCs. The frequency of “true” HSCs in the sorted populations of HSCs used for the experiments were not significantly different between distinct experimental groups (chi-squared test: *p* < 0.6264 for young versus aged; *p* < 0.9373 for young versus young + Wnt5a; *p* < 0.1042 for aged versus aged + CASIN; *p* < 0.2376 for young versus aged + CASIN; *p* < 0.6061 for aged versus young + Wnt5a).
(TIF)

S7 Fig Aged HSCs are found in clusters within the bone marrow. (A) Representative images of whole-mount preparations of long bones. This preparation allows to preserve structure and cell localization inside the bone. Please see Materials and methods for further details (graphical sources: https://www.servier.de/medical-art). (B) Cartoon scheme showing how distances between cells were measured based on the centroid of the cell in the 3D image. HSCs were considered adjacent (and thus no cell in between) when the distance “centroid to centroid” was less than 19 μm (largest HSC radius 7 μm, smallest BM cell radius 5 μm, 7 + 7 + 5). (C) Percentage of young and aged HSC found adjacent to each other in young and aged femurs. Data refer to 27 young and 14 aged longitudinal shaved whole-mount cross-sections from 2 young and 2 aged mice. 144 young HSCs and 394 aged HSCs. *p < 0.05. (D) Percentage of HSCs found in clusters (2 cells or more, each 3D image 500 μm × 500 μm for the *xy* plane and 50 μm along the *z* plane) in young and aged femora. Imaging data refer to 27 young and 14 aged longitudinal shaved whole-mount cross-sections from 2 young and 2 aged mice. 144 young HSCs and 394 aged HSCs were scored. ****p < 0.0001. (E) Percentage of donor-derived RFP+ young and aged HSCs found in clusters (2 cells or more, each 3D image 500 μm × 500 μm for the *xy* plane and 50 μm along the *z* plane) in femora of young recipient mice. Imaging data refer to 8 young and 4 aged longitudinal shaved whole-mount cross-sections from 2 young and 2 aged mice. 75 young HSCs and 108 aged HSCs were scored. ****p < 0.0001.
(TIF)

S8 Fig (A) A t-SNE **(**or TSNE) plot based on the global gene expression profile of all single cells analyzed. Three TSNE components are shown in the matrix plot, where points depict every single cell (O = aged [blue]; Y = young [orange]). Different shapes represent the 3 experimental arms (CASIN = circle; CONTROL = triangle; WNT5A = rectangle). Based on a combination of TSNE_3 with either TSNE_1 or TSNE_2, cells tend to segregate along the age axis. (B) Similarly, 3 PCs generated in a PCA of the global gene expression profile cumulatively explain 35% of the variation in the dataset. Colors represent aged groups (O = aged [blue]; Y = young [orange]), while shapes depict the 3 experimental arms (CASIN = circle; CONTROL = triangle; WNT5A = rectangle). PC, principal component; PCA, principal component analysis; t-SNE, t-Distributed Stochastic Neighbor Embedding.
(TIF)

S9 Fig (A) A QC scatter plot showing the relationship between expression (FPKM; in log 10 scale) and the number of cells, i.e., the number of cells expressing a given gene. The relationship basically shows that genes with relatively higher expression normally tend to be expressed by the majority of cells. (B) An overview of the cumulative proportion of each library (global or total expression for a given cell) as a function of the top-expressed features/genes. Plots are shown separately for each experimental arm and age group (aged and CASIN = blue; young and WNT5A = orange). The QC plot shows cell-to-cell difference/variation in distribution of expression. (C) A QC representative plot showing the level of gene expression of some randomly selected genes. The y-axis shows expression levels (log scale), while the x-axis shows the age group. Cells are represented by dots in the plot, and the colors reflect the 3 experimental arms (CASIN = blue; CONTROL = orange; WNT5A = green). (D) Venn diagram showing the overlap between the 4 experimental arms based on genes down-regulated in young and aged CASIN-treated HSCs. FPKM, Fragments Per Kilobase Million; QC, quality control.
(TIF)

S10 Fig 3D-SOMs generated for all daughter pairs analyzed. (PDF)

S11 Fig Radar plots of the GSEA showing all daughter pairs. We simultaneously interrogated 13 previously published hematopoietic stem cell and polarity signatures, and each daughter cell pair was depicted in a single radar plot where vertices in the plot correspond to each of the considered gene set. Pairs were then compared for significance of similarity/dissimilarity by a goodness of fit test across all significant gene sets.
(PDF)

S12 Fig (A) Representative plots presenting the relationship between number of mapped reads (x-axis) and number of peaks called (y-axis) across pairs A (left panel) and B (right panel). Blue points represent the observed peak counts (raw), while red points depict the number of peaks per million reads (adjusted). (B) Data identical to panel A are shown in a density plot where the x-axis represents peak counts and the y-axis shows density/frequency. Blue and red polygons represent the raw and adjusted peak counts, respectively. (C) The ratio of the peak number of paired daughters from young (green) and aged (blue) HSCs. A ratio of one represents an equal number of peaks in paired daughter cells. Daughter pairs of young HSCs present with higher ratios than daughter pairs from aged HSCs. The right panel shows the overall distribution of the ratio of the peaks for paired daughters of young and aged HSCs.
(TIF)

S13 Fig (A, B) Relationship between fragment length and normalized read density for 2 selected pairs (A = young pair 3y7A/3y7B; B = aged pair 3a2A/3a2B). The plot shows the characteristic distribution for ATAC-seq, mainly a large number of reads less than 100 bp and multiple periodicity. (C, D) Heatmaps showing an enrichment of the signal of the nucleosome-free fraction around active TSSs of selected cell pairs (C = young pair 3y7A/3y7B; D = aged pair 3a2A/3a2B). Each heatmap is accompanied by a bottom panel showing the mean distribution of the read coverage where the x-axis shows the distance from the TSS (in base pairs) and the y-axis shows the average read coverage per million mapped reads. (E, F) Comparative heatmap of the nucleosome-free and the mononucleosome signal across all active TSSs for representative pairs (E = young pair 3y7A/3y7B; F = aged pair 3a2A/3a2B). Nucleosome-free fragments, as presented also in panels C and D, show a unimodal enrichment around TSSs, while nucleosome bound fragments show phased enrichment both upstream and downstream of the active TSSs. (G, H) Line plots showing the same data as in panels E and F, where the x-axis shows the position around the TSSs and the y-axis represents the fraction of the signal. Full black lines depict nucleosome-free signals, while dashed red lines show nucleosome bound (mononucleosome) signals. (I, J) Genome-wide occupancy (ATAC-seq footprint) of a selected TF in representative daughter cell pairs (I = young pair 3y7A/3y7B; J = aged pair 3a2A/3a2B). The x-axis shows the distance to the motif of the TF (shown in the inset base logos), and the y-axis indicates the transposase cut-site probability. Forward and reverse strand signals are shown in blue and red lines, respectively.
(TIF)

S14 Fig (A) Percentage of the peaks assigned to distinct genomic regions. ATAC-seq peaks were primarily aligned to introns, intergenic regions 1 kb apart from the gene and at TSSs. There was no difference in the distribution of peaks between young and aged daughter cells. (B) Heatmap depicting the frequency of daughter stem cells showing a significant enrichment for specific TFs (the TFs most common to all pairs were selected). TFs were ranked (descending) based on the frequency of their peak count in daughter cells from young HSCs. (C, D) *p*-Value (panel C) and percentage of target sequences with a significant motif enrichment and of background sequences (panel D) for each of the TFs depicted in panel B. *Significantly (*p* < 0.05) different percentage of target sequences with a significant motif enrichment. (E) Heatmap depicting the frequency of daughter progenitor cells showing a significant enrichment for specific TFs (the TFs most common to all pairs were selected). TFs were ranked (descending) based on the frequency of their peak count in young daughter stem cells. (F, G) *p*-Value (panel F) and percentage of target sequences with a significant motif enrichment and of background sequences (panel G) for each of the TFs depicted in panel E. (H) Graph showing the logP values for the GSEA for genes included in a previously published differentiation/proliferation gene set (BRUNO_HEMATOPOIESIS). This gene set includes genes that changed expression when multipotent cells underwent differentiation and thus lost self-renewal and proliferation properties. Only 2 aged and 3 young daughter progenitor cells showed a significant enrichment. Daughter cells were grouped as progenitor cells according to Fig 8F. (I) Heatmap showing genes that are common between a published differentiation/proliferation gene set (GSEA for BRUNO_HEMATOPOIESIS) and the annotated peaks of the daughter cell with a higher peak count in a given pair (such daughter cells were scored as progenitor cells according to Fig 8F). Genes in the heatmap are ranked (in descending order) based on the frequency of commonality across daughter cells (young = left heatmap; aged = right heatmap). A distinct set of genes is common to cells in a given progenitor age group but also to both progenitor age groups, implying a central role of these genes for progenitor specification.
(TIF)

S15 Fig Venn diagrams identifying the peak count overlap for each daughter cell pair from young and aged HSCs. (PDF)

S16 Fig Radar plots of the GSEA for all daughter pairs. We simultaneously interrogated the correlation to 13 previously published hematopoietic stem cell and polarity signatures [32–39], and each daughter cell pair was depicted in a radar plot where vertices in the plot correspond to each of the considered gene sets: Mm_HSC_Runx1_Wu [32], Mm_HSC_Tcf7_Wu [32], Mm_LT_HSC_Venezia [36], Mm_Proliferation_Venezia [36], Mm_Quiescence_Venezia [36], Polarity_factors_Ting [37], Novel_HSC_regul_polar_Ting [37], Grover et al, 2016 [38] and Kowalczyk et al, 2015 [39]. Gene signatures that showed a significant enrichment based on a hypergeometric test (adjusted *p* < 0.05) are shown by red and blue shades representing the 2 cells in a given pair. Signatures not extending beyond the shaded gray center were considered not significant.
(PDF)

S17 Fig Venn diagrams identifying the overlap for significant TF motif enrichment for each daughter cell pair from young and aged HSCs. (PDF)

S18 Fig Venn diagrams identifying the overlap for significantly enriched Reactome GOs for each daughter cell pair from young and aged HSCs. (PDF)

S1 Table Analysis of the frequency of symmetric/asymmetric divisions based on Cdc42 allocated to daughter cells. For each single sample set, the number and frequency of symmetric and asymmetric pairs is listed. The *p*-value refers to the chi-squared test. The decision on whether a cell divided symmetrically or asymmetrically was based on the ration of the Cdc42 volume in paired daughter cells (see graph Fig 1D). The threshold was set very conservatively to at least a one-fourth volume reduction (75% reduction in graph of Fig 1D): above the threshold, the division was scored as symmetric; below the threshold, it scored as asymmetric.
(PDF)

S2 Table Analysis of the divisional outcome. The outcome of each paired single daughter transplant is shown. Asym, asymmetric; DA, daughter A; DB, daughter B; n.e., not engrafted; P, cell retrospectively defined as progenitor; S, cell retrospectively defined as stem cell; SymS, symmetric self-renewal; SymD, symmetric differentiation.
(PDF)

S3 Table Analysis of the frequency of reconstitution. For each single sample set, the number and frequency of single and pair-wise mice are listed. The *p*-value refers to the chi-squared test based on the symmetric and asymmetric divisional outcomes.
(PDF)

S4 Table Table showing the number of cells sequenced, number of cells used for downstream analysis, and number of genes filtered. The table also shows how many cells per arm/condition were finally used.
(XLSX)

S5 Table List of the gene sets used in the hypergeometric analysis (stem-ness and polarity signature set). (XLS)

S6 Table List of the 134 genes up-regulated in the daughter cells from the young and aged CASIN-treated arms but not in the aged HSCs and young Wnt5a-treated ones. (XLS)

S7 Table List of the 140 genes up-regulated in the daughter cells from the aged and young Wnt5a-treated HSCs but not in young and aged CASIN-treated arms. (XLS)

S8 Table The number of peaks in each single daughter cell, the number of peaks common to pairs, the mean predicted overlap according to the simulation test of the null hypothesis that cells in a pair show the same peak counts in the same genomic position, the raw and adjusted peak ratio, the number of mapped reads, their significance, and the final score for the mode of the division (asymmetric or symmetric). (XLSX)

S9 Table Significant TF motif output common to individual pairs of daughter cells. (XLSX)

S10 Table Significantly enriched Reactome GOs common to individual pairs of daughter cells. (XLSX)

S1 Movie 3D-IF reconstruction of Cdc42 in a dividing (telophase) young HSC. DAPI (nucleus, blue), Cdc42 (magenta), and tubulin (green).
(AVI)

S2 Movie 3D-IF reconstruction of Cdc42 in a dividing (telophase) aged HSC. DAPI (nucleus, blue), Cdc42 (magenta), and tubulin (green).
(AVI)

S3 Movie 3D-IF reconstruction of Cdc42 in a dividing (telophase) aged + CASIN HSC. DAPI (nucleus, blue), Cdc42 (magenta), and tubulin (green).
(AVI)

S4 Movie 3D-IF reconstruction of Cdc42 in a dividing (telophase) young + Wnt5a HSC. DAPI (nucleus, blue), Cdc42 (magenta), and tubulin (green).
(AVI)

S5 Movie 3D-IF reconstruction of H4K16ac in a dividing (telophase) young HSC. DAPI (nucleus, blue), H4K16ac (magenta), and tubulin (green).
(AVI)

S6 Movie 3D-IF reconstruction of H4K16ac in a dividing (telophase) aged HSC. DAPI (nucleus, blue), H4K16ac (magenta), and tubulin (green).
(AVI)

S7 Movie 3D-IF reconstruction of H4K16ac in a dividing (telophase) aged + CASIN HSC. DAPI (nucleus, blue), H4K16ac (magenta), and tubulin (green).
(AVI)

S8 Movie 3D-IF reconstruction of H4K16ac in a dividing (telophase) young + Wnt5a HSC. DAPI (nucleus, blue), H4K16ac (magenta), and tubulin (green).
(AVI)

## Abbreviations

3D-IF: 3D confocal immunofluorescence
BGA: between-group analysis
CASIN: cdc42 activity specific inhibitor
Cdc42: cell division control protein 42
FPKM: fragments per kilobase of exon per million fragments mapped
GDP: guanosine diphosphate
GO: gene ontology
GTP: guanosine triphosphate
HSC: hematopoietic stem cell
OCT: optimum cutting temperature
PB: peripheral blood
PBS: phosphate-buffered saline
PFA: paraformaldehyde
QCR: quadrant count ratio
RFP: red fluorescent protein
scATAC-seq: single-cell transposase-accessible chromatin sequencing
scRNA-seq: single-cell RNA sequencing
SOM: self-organizing map
TF: transcription factor
TSS: transcription start site
