# Supplementary material for: Aging alters the epigenetic asymmetry of HSC division
Source: PLoS Biol. 2018 Sep 20;16(9):e2003389. doi: 10.1371/journal.pbio.2003389 (PMC6168157; doi:10.1371/journal.pbio.2003389)
Supplement: S1 Text — (DOCX) [file pbio.2003389.s002.docx]

***Supplement Material***

**Mathematical Modeling of Aging-Related Changes in Polarity and Symmetry of Division in HSCs**

We first investigated which mode of division among all possibilities was the most likely one (**Fig 3A**, grey arrows). We applied a Maximum-Likelihood (ML) approach by simulating the transition probabilities of LT-HSC division and divisional outcome according to the transition rates given by our experimental data (**Fig 2C-F**). The optimization procedure yielded the following probabilities (based on $n={5\cdot10}^{4}$ simulated cell divisions):

- $d_{a}\approx0.96$ for a polar LT-HSC to divide asymmetrically
- $d_{s}\approx0.82$ for an apolar LT-HSC to divide symmetrically

As a control, we re-ran a different simulation experiment, in which we chose $d_{a}=d_{s}=0.5$, i.e. the probabilities of LT-HSCs to undergo either asymmetric or symmetric cell divisions were the same for both polar and apolar LT-HSCs. As expected, we found equal frequencies for asymmetric and symmetric division among young and aged LT-HSC populations, which does not reflect the observed data.

In conclusion, there is strong evidence for a link between LT-HSC polarity/apolarity and asymmetric/symmetric mode of division.

We proceeded by developing a multi-layer mathematical model in order to describe aging-related changes in long-term hematopoietic stem cells (LT-HSCs). We have designed the model based on a set of findings that link *Cdc42* activity, cell polarity, histone acetylation and symmetry of cell division[1, 2]. We use an ordinary differential equation (ODE) based model to describe the cell-individual concentration and activity of *Cdc42* as well as the level of *H4K16* acetylation. On a higher level, a cell division process is incorporated as a simple spatial model, in which the level of intracellular *Cdc42* activity determines the local arrangement of *Cdc42* and *AcH4K16* and thereby regulates the differential inheritance of cellular components to the offspring cells (polar vs. apolar division). In particular, we make the following assumptions (see the sketch in **Fig S3A**):

1. *Cdc42 transcription*. LT-HSCs and progenitors differ in their *Cdc42* concentration. In particular, LT-HSCs on average show a 2-fold increase of *Cdc42* concentration compared to differentiated (progenitor) cells (**Fig S3I**). We therefore assume that *Cdc42* is auto-regulative such that a sufficiently high concentration promotes its own production. In terms of an ODE this reads:

$\dot{c}_{\text{tot}}\left( t \right)=b_{c}+\frac{k_{c}\cdot c_{\text{tot}}\left( t \right)^{\gamma}}{\alpha+c_{\text{tot}}\left( t \right)^{\gamma}}-c_{\text{tot}}\left( t \right)$( 1 )

In a transcriptional interpretation, the first term refers to a low background transcription ($b_{\text{c}}$) while the last term indicates a linear decay process (${-c}_{\text{tot}}(t)$) of *Cdc42*. The intermediate term represents the (transcriptional) auto-regulation. For $\gamma>1$such a system allows for bistability, i.e. *Cdc42* can be stably present at either a low or a high level. In the low state, *Cdc42* concentration is not sufficient to stimulate its own production, while in the high state the auto-regulation is the major contributor. In mathematical terms the two states represent two stable fixed points existing simultaneously for a certain range of parameters. The acquired fixed point for a given model realization depends on the initial *Cdc42* concentration, e.g. after cell division (**Fig 3C-D**). We associate the *Cdc42*-high state with a LT-HSC and the *Cdc42*-low state with a differentiated (progenitor) cell. Thus, *Cdc42* acts as a switch determining the cell’s identity after cell division.

1. *Cdc42 activity*. Being a small RhoGTPase, *Cdc42* cycles between an active (*GTP*-bound) and inactive (*GDP*-bound) state[1]. Therefore, we explicitly distinguish between active and inactive *Cdc42* (*Cdc42-GTP* vs. *Cdc42-GDP*). By assuming a fast phosphorylation reaction, the equilibrium concentrations can be described by the law of mass action as follows:

$c_{\text{GTP}}\left( c_{\text{tot}} \right)=\frac{k_{+}}{k_{+}+k_{-}}c_{\text{tot}}$ ( 2 )

Herein, *Cdc42-GTP* ($c_{\text{GTP}}$) is produced with constant rate $k_{+}$and decays with constant rate $k_{-}$; $c_{\text{tot}}$is given as the sum of $c_{\text{GTP}}$ and $c_{\text{GDP}}$. The amount of *Cdc42* activity $c_{\text{GTP}}$ solely depends on the total concentration of *Cdc42* $c_{\text{tot}}$.

1. *Acetylation of histone H4 lysine 16 (AcH4K16)*. It was reported that a decrease in *H4K16* acetylation is associated with increasing *Cdc42* activity (*Cdc42-GTP*)[1]. In order to incorporate this anti-correlation, we assume that *AcH4K16* levels are negatively regulated by *Cdc42-GTP*. Although there is no evidence for a direct interaction, for reasons of simplicity we design the model as such. The corresponding ODE reads as follows:

$\dot{n}_{\text{ac}}\left( t \right)=b_{\text{ac}}+\frac{k_{\text{ac}}(N_{\text{ac}}-n_{\text{ac}}(t))}{1+exp(\vartheta\cdot c_{\text{GTP}}^{*})}-n_{\text{ac}}\left( t \right)$( 3 )

Herein, $n_{\text{ac}}(t)$ describes the number of acetylated histones at time $t$ and $N_{\text{ac}}$ refers to the maximum number of histones to be potentially acetylated. The number of acetylated histones at each time step is constantly inhibited by *Cdc42* activity $c_{\text{GTP}}^{*}$ until a fixed point for the fraction of acetylated histones is reached. Thus, for each fixed point $c_{\text{GTP}}^{*}$ of *Cdc42* activity there exists a corresponding acetylation level of *H4K16*.

1. *Aging LT-HSCs*. It was reported that an increase of *Cdc42* activity upon aging may result from a shift from canonical to non-canonical *Wnt* signaling (namely elevated *Wnt5a* expression), whereas total *Cdc42* concentration remains constant[1, 2]. We incorporate this *aging effect* as an increase of the phosphorylation constant *k*_+_ (equation 2) with age. This leads to a higher fraction of activated *Cdc42* while the level of total *Cdc42* remains unaltered.
2. *Cell Polarity*. It has been demonstrated that a (e.g. aging-related) increase of *Cdc42* activity correlates with a loss of cell polarity with respect to *Cdc42* localization within the cell and *AcH4K16* within the cell nucleus[1]. Within the model we assume that the level of *Cdc42* activity itself regulates the spatial distribution. We make the simplest assumption that cells are described as circles. The spatial distributions of *Cdc42* and *AcH4K16* can be mapped onto the outline and are described as angular densities. In order to parameterize the local arrangements, we describe the angular densities of *Cdc42* in the cell (and of *AcH4K16* within the cell nucleus) by a normal distribution $N(\mu,\sigma^{2})$ on the cell’s circular outline. Here, $\mu$indicates the position of the highest concentration, whereas the variance $\sigma^{2}$ describes the width of the distribution.

In this picture, the variance $\sigma^{2}$ is the crucial parameter to determine whether the density is rather concentrated at one pole or whether there is a broad distribution (**Fig S3B**). Whereas a small variance corresponds to a polar pattern, a large $\sigma^{2}$ refers to an apolar pattern (see **Fig S3C**). As we assume that the level of *Cdc42* activity (*Cdc42-GTP*) itself regulates its spatial distribution, we directly couple the variance $\sigma^{2}$ of the angular *Cdc42* distribution to the level of activated *Cdc42* (**Fig S3B**). As a consequence, low *Cdc42* activity corresponds to a low $\sigma^{2}$ resulting in a polar distribution of *Cdc42* protein, while increased *Cdc42* activity, e.g. during aging, increases the variance and destabilizes the polar pattern. We furthermore make the assumption that both active and inactive *Cdc42* are distributed in the same location to ensure a stable balance between inactivation and activation facilitated by a constant (de-)phosphorylation.

For the acetylation level of *H4K16* we assume a localization opposite to the distribution of active *Cdc42* according to our findings[1]. This is modeled as a point reflection of the $\sigma^{2}$-dependent *Cdc42* activity distribution in the circle center. As a result, in the case of low *Cdc42* activity, *AcH4K16* is distributed in a polar way as a consequence of a small variance $\sigma^{2}$ and locates opposite to the (polar) *Cdc42* distribution, whereas in the case of elevated *Cdc42* activity *AcH4K16* is distributed in an apolar manner (**Fig S3C**).

Although the precise mechanisms linking *Cdc42* activity and spatial patterning are not fully understood yet, we explicitly incorporate this direct relation in our models. The consistency between the resulting model and the available data is interpreted as a further hint to study this interaction more closely.

1. *Cell Division.* Cell divisions segregate the cellular content of a mother cell towards two offspring daughter cells. Within the model a division is formally defined as separation of the *circular cell* along a randomly chosen, perpendicular plane through its center. This leads to a separation of the levels of *Cdc42* and *AcH4K16* according to the density within each of the “halves”. These levels serve as initial values for the intracellular dynamics described by the ODE model (steps 1 – 3).

In the case of low *Cdc42* activity, both total *Cdc42* and *AcH4K16* have a polar distribution within the circular cell object. Consequently, cell division along a randomly chosen plane predominantly results in division events, in which a high fraction of total *Cdc42* is inherited to one daughter cell while the other one receives a smaller fraction (asymmetric division, see **Fig 3C** and **Fig S3D**). In contrast, for high *Cdc42* activity and thus rather evenly distributed *Cdc42* and *AcH4K16* in the cell, the division events result in daughter cells with similar ratios of the two components (symmetric division, see **Fig 3D** and **Fig S3E**). Cellular identity is now re-established by reaching either of the two stable fixed points emerging from the auto-regulation of *Cdc42* transcription (see step 1). Thus, *Cdc42* acts as a molecular switch determining the cell’s identity.

Model Behavior

*Homogeneous cell populations*. In order to study the dynamic features of the suggested model, we first consider two exemplary settings, which are homogeneous populations of only young and only aged LT-HSCs. In our interpretation, these cells only differ by their specific values for the *Cdc42* activation constant $k_{+}$. Thus, the cells are different in their ability to convert *Cdc42-GDP* into *Cdc42-GTP*. As a result, the populations are also homogeneous with respect to their polarity patterns – young LT-HSCs have low *Cdc42* activity levels leading to polar distributions of *Cdc42* and *AcH4K16* whereas aged LT-HSCs show a higher *Cdc42* activity thereby contributing to apolar distributions. We initialize the respective cell populations and let them arbitrarily divide according to the cell division mechanism introduced above. As a result, in young LT-HSC divisions we almost exclusively find asymmetric cell divisions with respect to *Cdc42* distribution in daughter cells after cell division (**Fig S3D**, green pie chart). In contrast, aged LT-HSC divisions are mostly symmetric leading to two similarly equipped daughter cells (**Fig S3E**, green pie chart). While in the case of young LT-HSCs the dynamic re-establishment of *Cdc42* concentrations yields different outputs for the two daughter cells (namely one *Cdc42*-high cell = LT-HSC; and one *Cdc42*-low cell = progenitor), in the case of aged LT-HSCs the daughters share the same fate like their mothers (**Fig S3D-E**, white/yellow pie charts).

*Heterogeneous cell populations*. In order to mimic the observed polar to apolar cell ratios within experimentally accessible populations of LT-HSCs from young and aged donors, we assume that these populations are intrinsically heterogeneous. To reflect this within the model we choose values for the activation rate $k_{+}$ from a normal distribution. The mean of the distributions, from which the random variable is chosen, corresponds to the $k_{+}$ values used for the homogeneous populations studied above (**Fig S3H**), while the standard deviation is fixed such that young LT-HSC populations present with a 2:1 ratio of polar to apolar cells and aged LT-HSC populations with a 1:5 ratio. Consequently, for the young LT-HSC population we observe a ratio of asymmetric vs. symmetric cell divisions similar to the experimental observation (**Fig 4C**, **Fig 3E** and **Fig S3F**). For the aged LT-HSC population the fraction of polar cells predominantly contributes to asymmetric division events, while the majority of cells preserve the maternal LT-HSC phenotype of their mother’s due to a symmetric replication. Both asymmetric cell division and final cell number proportions closely resemble the proportions observed in our experiments (**Fig 4C**, **Fig 3E** and **Fig S3G**).

In addition to the findings above, the model also describes another observation previously reported [1, 3-5], namely an increase in LT-HSC numbers with age. The simulation results of our model suggest that due to the loss of cell polarity the number of asymmetric cell divisions decreases. Consequently, in aged LT-HSCs, the proportion of LT-HSCs after cell division increases in comparison to young LT-HSCs since the cells are no longer able to pass the transcriptional switch provided by *Cdc42*.

In conclusion, the heterogeneous population model is able to describe the experimental cell division behavior for populations of both young and aged LT-HSCs. Although our model supports the notion that *Cdc42* itself acts as an age-dependent molecular switch at the onset of differentiation, alternative explanations cannot be ruled out. In fact, any co-regulator of *Cdc42* with a similar expression profile can be of equal importance to facilitate the switch.

**References**

1. Florian MC, Dorr K, Niebel A, Daria D, Schrezenmeier H, Rojewski M, et al. Cdc42 activity regulates hematopoietic stem cell aging and rejuvenation. Cell Stem Cell. 2012;10(5):520-30. Epub 2012/05/09. doi: S1934-5909(12)00172-5 [pii]

10.1016/j.stem.2012.04.007. PubMed PMID: 22560076; PubMed Central PMCID: PMC3348626.

2. Florian MC, Nattamai KJ, Dorr K, Marka G, Uberle B, Vas V, et al. A canonical to non-canonical Wnt signalling switch in haematopoietic stem-cell ageing. Nature. 2013;503(7476):392-6. Epub 2013/10/22. doi: nature12631 [pii]

10.1038/nature12631. PubMed PMID: 24141946.

3. Dykstra B, Olthof S, Schreuder J, Ritsema M, de Haan G. Clonal analysis reveals multiple functional defects of aged murine hematopoietic stem cells. J Exp Med. 2011. Epub 2011/11/24. doi: jem.20111490 [pii]

10.1084/jem.20111490. PubMed PMID: 22110168.

4. Ergen AV, Goodell MA. Mechanisms of hematopoietic stem cell aging. Exp Gerontol. 2009. Epub 2009/12/26. doi: S0531-5565(09)00313-1 [pii]

10.1016/j.exger.2009.12.010. PubMed PMID: 20034552.

5. Rossi DJ, Bryder D, Zahn JM, Ahlenius H, Sonu R, Wagers AJ, et al. Cell intrinsic alterations underlie hematopoietic stem cell aging. Proc Natl Acad Sci U S A. 2005;102(26):9194-9. PubMed PMID: 15967997.
