# Supplementary figures and images for: Aging alters the epigenetic asymmetry of HSC division
Source: PLoS Biol. 2018 Sep 20;16(9):e2003389. doi: 10.1371/journal.pbio.2003389 (PMC6168157; doi:10.1371/journal.pbio.2003389)

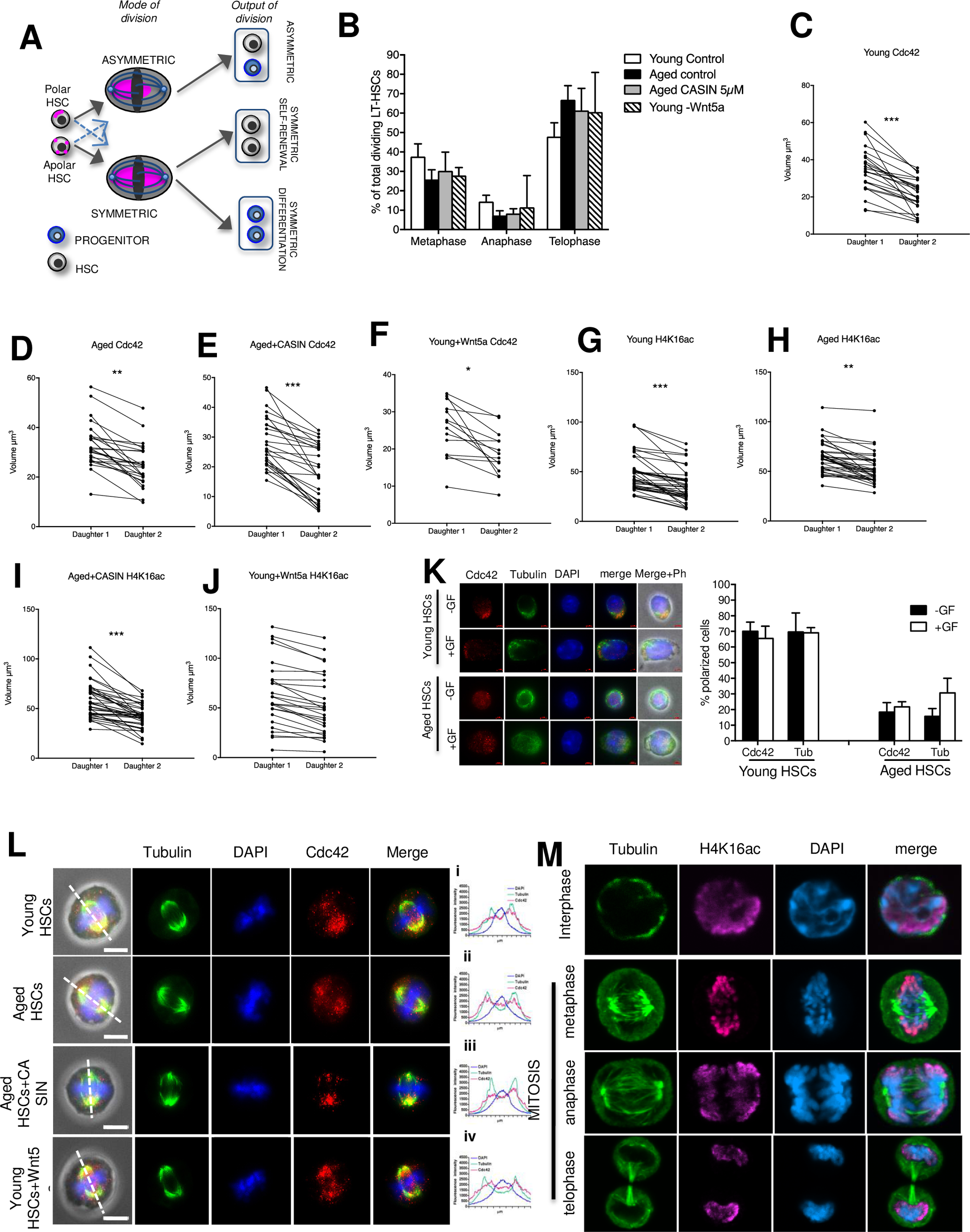

Supplement: S1 Fig — (A) Schematic representation of the possible mode and outcome of polar and apolar HSC divisions. The establishment of cell polarity within the mother HSC is supposed to be one fundamental mechanism that controls mode and outcome of asymmetric stem cell division. Such stem cell asymmetric division allows one daughter cell to become differentiated and the other to retain stem cell potential; in contrast, symmetric division allows both daughter cells to adopt equivalent fates. (B) Percentages of HSCs in metaphase, anaphase, or telophase among total mitotic HSCs. (C–J) Quantification of the amount of Cdc42 and H4K16ac in nascent daughter cells. Each pair is connected by a line. *p < 0.05, **p < 0.01, ***p < 0.001; n = 2–3 biological repeats; 25 pairs for young, 26 pairs for aged, 26 pairs for aged + CASIN and 14 pairs for young + Wnt5a for panel C–F; 41 pairs for young, 37 pairs for aged, 40 pairs for aged + CASIN, and 26 pairs for young + Wnt5a for panel G–J. (K) Representative epifluorescence pictures of young and aged HSCs cultured with and without growth factors (GF; SCF, G-CSF, and TPO all 100 ng/mL). Panels show DAPI (nucleus, blue), Cdc42 (red), and tubulin (green). The column graph depicts the percentage of polar cells retrieved in each sample. n = 3 biological repeats. Culture conditions did not affect the percentage of polar HSCs in both young and aged cell preparations. (L) Representative epifluorescence pictures of dividing (metaphase) young, aged, aged treated with CASIN 5 μM, and young treated with Wnt5a 100 ng/mL HSCs. Panels show DAPI (nucleus, blue), Cdc42 (red), and tubulin (green). The dashed lines cross transversally the dividing cells touching both opposite poles. The fluorescence intensity was measured along the dashed line (panel M); representative 3D confocal reconstruction of HSCs stained during division. The images show tubulin (green), H4K16ac (magenta), and the nucleus (DAPI, blue). The total level of H4K16ac during all phases of mitosis (met [file pbio.2003389.s003.tif]

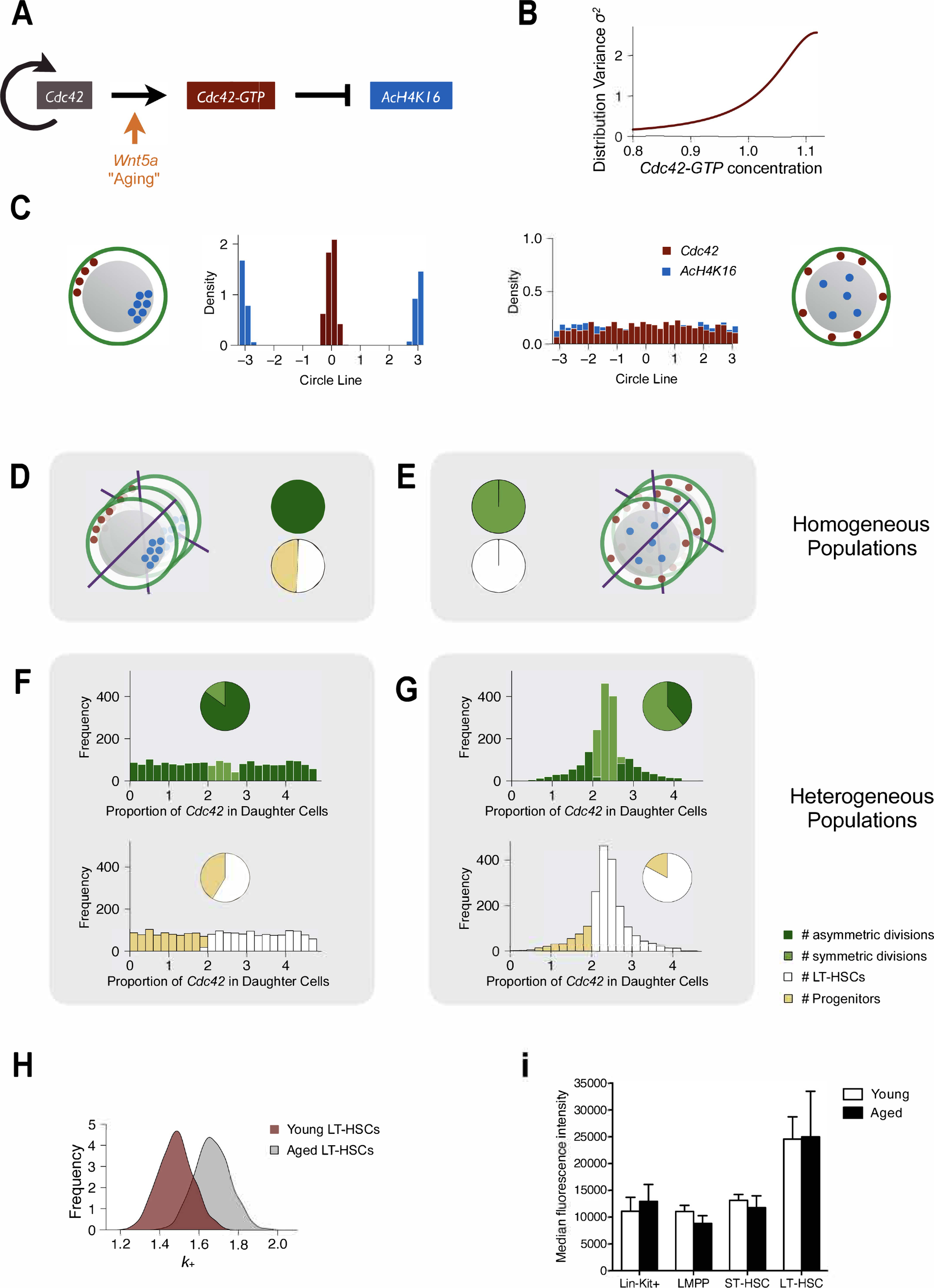

Supplement: S3 Fig — (A) Sketch of the ODE model describing intracellular dynamics. Total Cdc42 is assumed to be autoregulative while an age-dependent proportion is activated. Active Cdc42 inhibits the cell’s acetylation level. (B) The variance of Cdc42 distribution (as a measure of apolarity) increases with increasing Cdc42 activity. (C) Representation of a polar and an apolar cell, respectively, in terms of a normal distribution N(μ,σ2) of Cdc42 on the cell’s circular outline (and AcH4K16 within the cell nucleus opposite Cdc42 localization). The x-axis of the histogram represents the circle line ranging periodically from –π to π. Polar cells concentrate Cdc42 at a pole around zero while AcH4K16 is distributed opposite. In contrast, apolar cells show a rather uniform protein distribution due to an elevated variance σ2. (D) Cell division for a homogeneous polar cell population. All cells of the population show a similar polarity pattern and are divided by an arbitrary plane through their center. This division event leads to asymmetric cell divisions almost exclusively (dark green in upper pie chart) and, after running the cell-intrinsic process (ODE model), ends up with a close to 1:1 ratio of LT-HSCs (white) and progenitors (yellow). (E) Cell division for a homogeneous apolar cell population ends up with symmetric divisions and LT-HSC daughters only. (F–G) Cell division simulation for a population of 1,000 young and aged LT-HSCs, respectively, being heterogeneous with respect to polarity. Pie charts (correspond to simulation pie charts in Fig 3E) depict the ratio of asymmetric and symmetric cell divisions and the ratio of LT-HSCs and progenitors, respectively. The histograms depict the frequency of daughter cells with a certain Cdc42 concentration after cell division. Upper histograms relate the symmetry of division with the specific Cdc42 concentration in the daughters. Lower histograms relate the results of the intracellular ODE model with total Cdc42 concentration after cell divisio [file pbio.2003389.s005.tif]

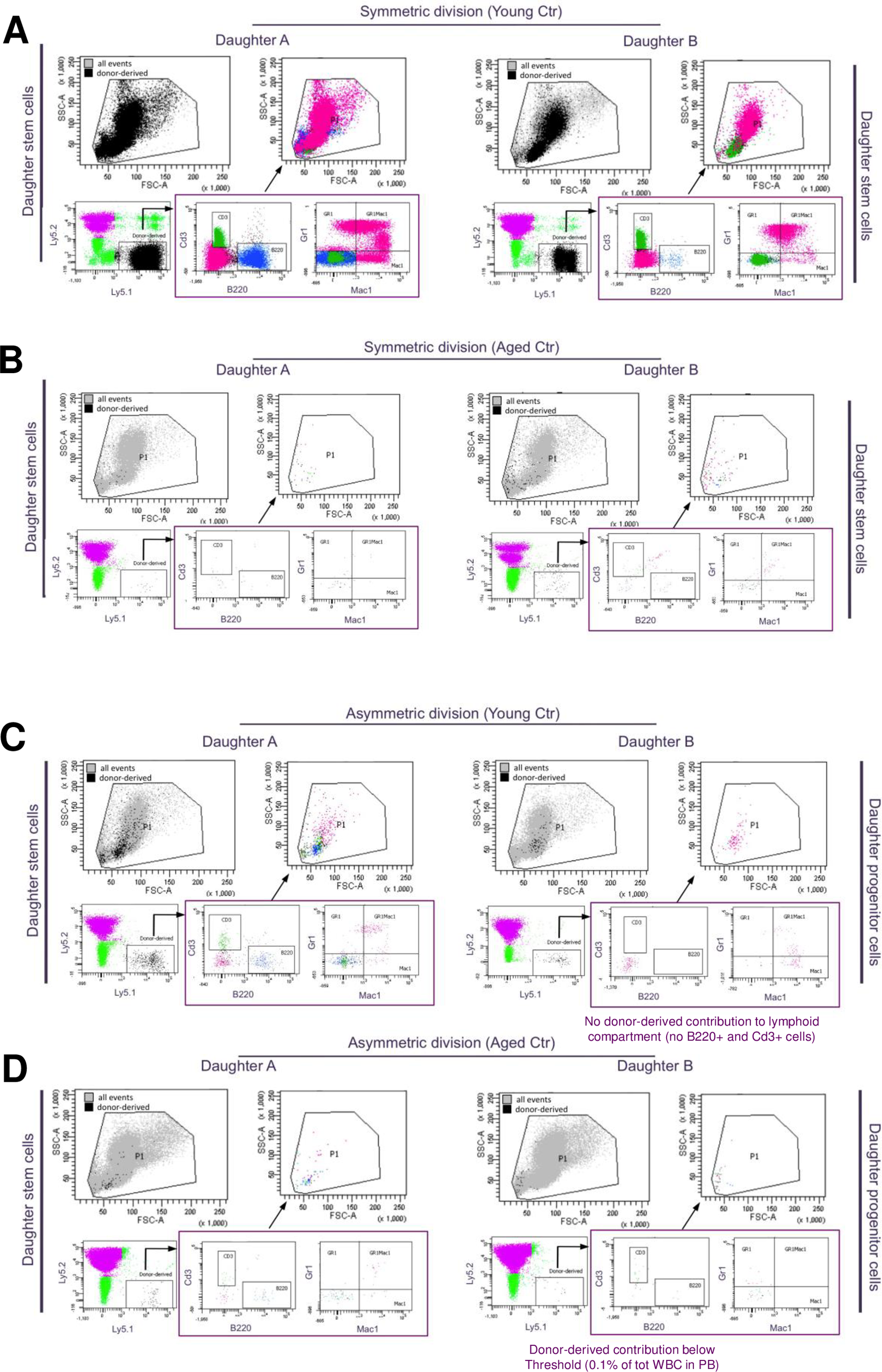

Supplement: S4 Fig — (A) Representative flow cytometry charts of PB from two mice 24 weeks post transplantion of individual but paired daughter cells (named arbitrarily A and B) that originated from a distinct young mother HSC. Donor-derived contribution to PB is higher than 0.1% of total WBC, and contribution is detected for both lymphoid (B cells, B220+ and T cells, Cd3+) and myeloid lineages (Gr1+, Mac1+, and Gr1+Mac1+cells). Consequently, the division of the mother HSC was scored as symmetric. (B) Representative flow cytometry charts of PB from two mice 24 weeks post transplantation of individual but paired daughter cells (named arbitrarily A and B) that originated from a distinct aged mother HSC. Donor-derived contribution to PB is higher than 0.1% of total WBC and the contribution is detected for both lymphoid (B cells, B220+ and T cells, Cd3+) and myeloid lineages (Gr1+, Mac1+, and Gr1+Mac1+cells). Consequently, the division of the mother HSCs was scored as symmetric. (C) Representative flow cytometry charts of PB from two mice 24 weeks post transplantation of individual but paired daughter cells (named arbitrarily A and B) that originated from a distinct young mother HSC. Donor-derived contribution to PB is higher than 0.1% of total WBC, but the contribution is detected for both lymphoid (B cells, B220+ and T cells, Cd3+) and myeloid lineages (Gr1+, Mac1+, and Gr1+Mac1+cells) only for daughter cell A. Daughter cell B shows no contribution to lymphoid cells (Cd3+ and B220+ cells). Consequently, the division of the mother HSCs was scored as asymmetric. (D) Representative flow cytometry charts of PB from two mice 24 weeks post transplantation of individual but paired daughter cells (named arbitrarily A and B) that originated from a distinct young aged HSC. Donor-derived contribution to PB is higher than 0.1% of total WBC only in daughter cell A. Daughter cell B shows a contribution to PB that is lower than 0.1% of total WBC. Consequently, the division of the mother HSCs was scored [file pbio.2003389.s006.tif]

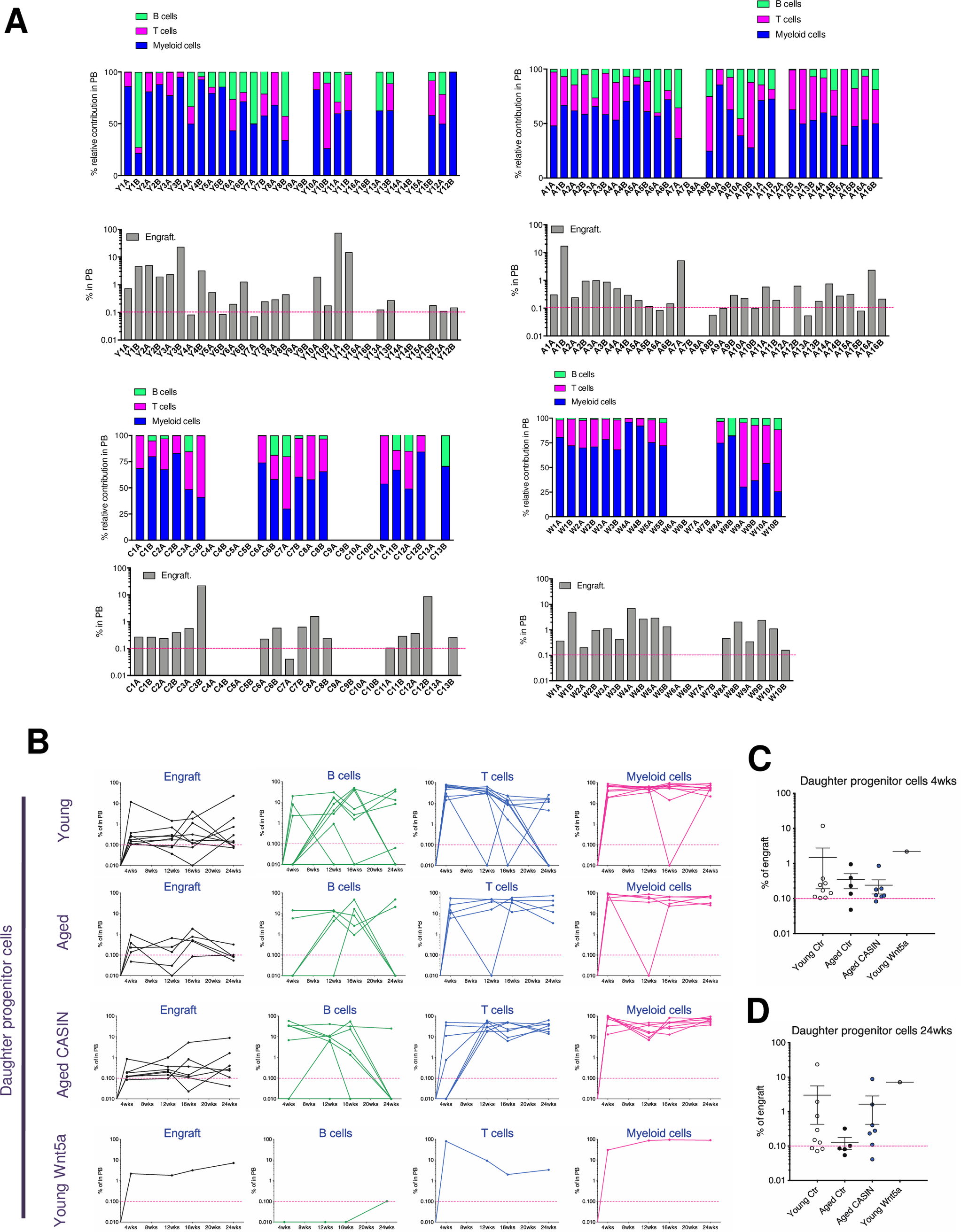

Supplement: S5 Fig — (A) Kinetics of the chimerism of the overall level of engraftment of donor-derived cells and of each donor-derived lineage (B cells, T cells, and myeloid cells). Shown are young, aged, aged treated with CASIN, and young treated with Wnt5a daughter cells that were retrospectively identified as daughter progenitor cells. (B–C) Percentage of donor-derived cells at 4 weeks and 24 weeks after transplantations in PB of mice transplanted with single (retrospectively identified) daughter progenitor cells. n = 9 for young, n = 5 for aged, n = 7 for aged + CASIN, and n = 1 for young + Wnt5a. (D) Engraftment and lineage contribution for each single-cell transplant analysed. Shown is the final time point (24 weeks). Each daughter pair is identified by a number and A/B. All underlying data for this figure can be found in S1_Data panels 5A and S5B (including data for S5A, S5C and S5D Fig). A, aged; C, aged + CASIN; W, young + Wnt5a; Y, young. (TIF) [file pbio.2003389.s007.tif]

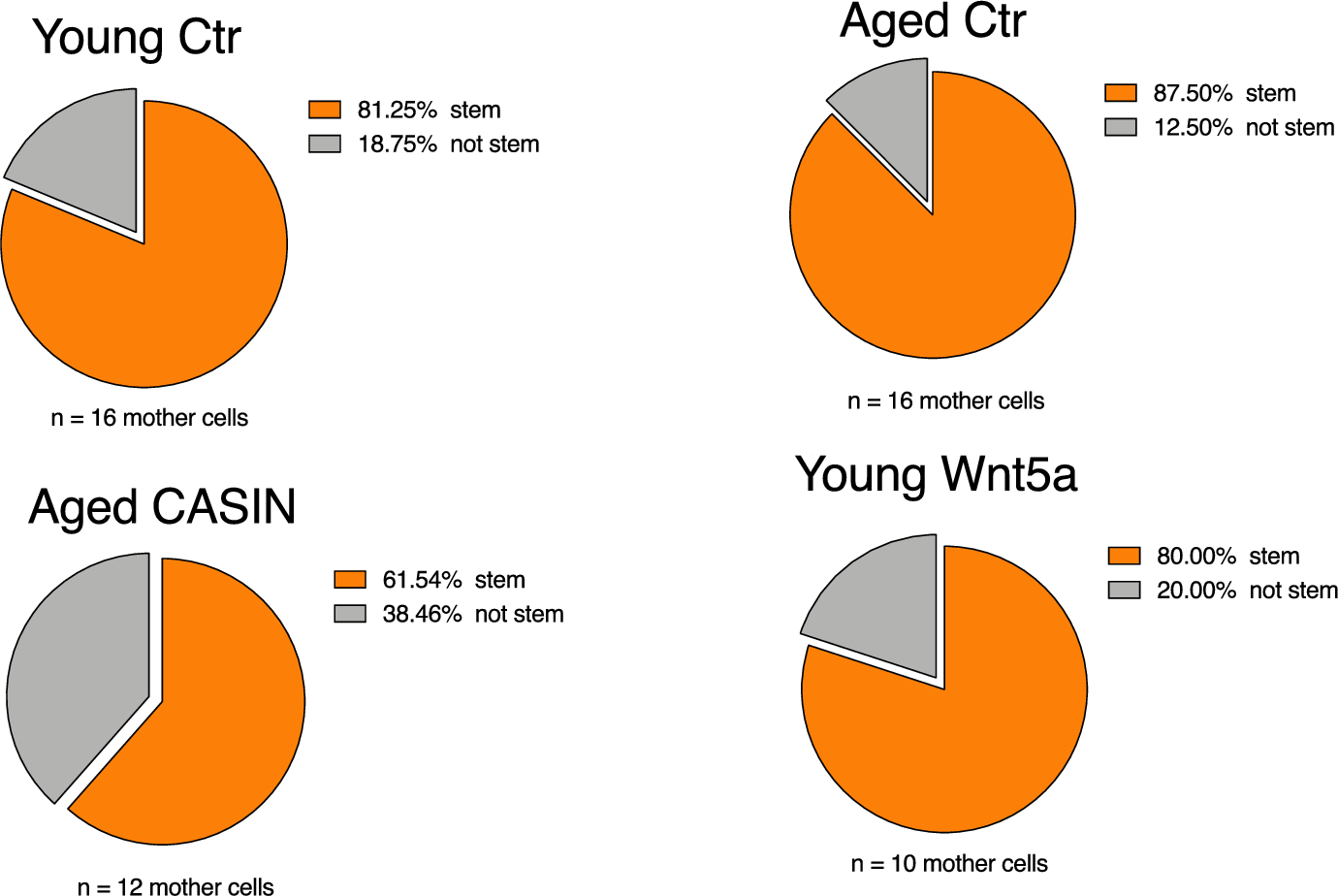

Supplement: S6 Fig — (A) Pie charts depicting the frequency of mother cells that generated at least one daughter stem cell. Since upon division they generated at least one daughter stem cell, the mother cells were scored as “true” HSCs. The frequency of “true” HSCs in the sorted populations of HSCs used for the experiments were not significantly different between distinct experimental groups (chi-squared test: p < 0.6264 for young versus aged; p < 0.9373 for young versus young + Wnt5a; p < 0.1042 for aged versus aged + CASIN; p < 0.2376 for young versus aged + CASIN; p < 0.6061 for aged versus young + Wnt5a). (TIF) [file pbio.2003389.s008.tif]

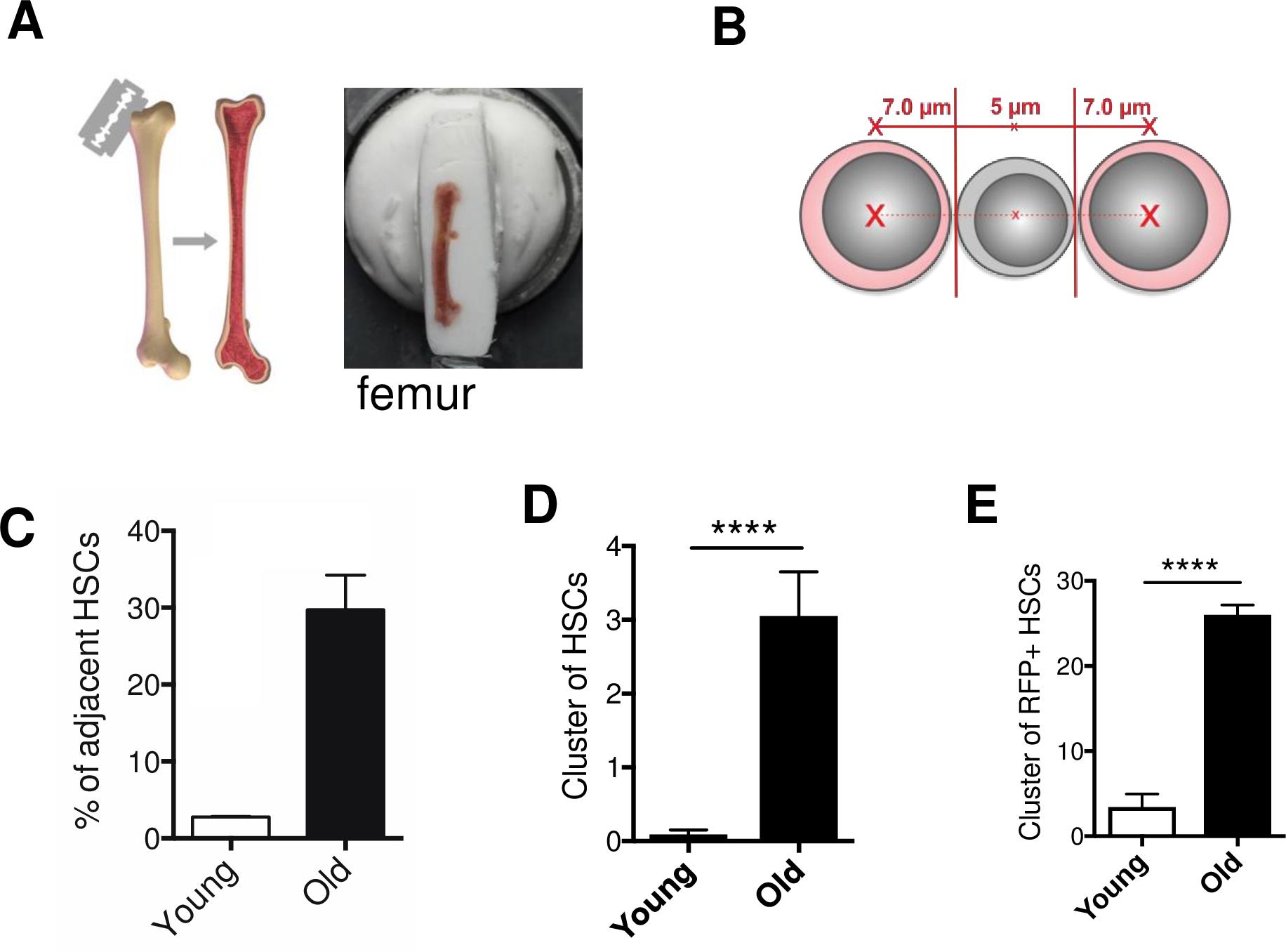

Supplement: S7 Fig — (A) Representative images of whole-mount preparations of long bones. This preparation allows to preserve structure and cell localization inside the bone. Please see Materials and methods for further details (graphical sources: https://www.servier.de/medical-art). (B) Cartoon scheme showing how distances between cells were measured based on the centroid of the cell in the 3D image. HSCs were considered adjacent (and thus no cell in between) when the distance “centroid to centroid” was less than 19 μm (largest HSC radius 7 μm, smallest BM cell radius 5 μm, 7 + 7 + 5). (C) Percentage of young and aged HSC found adjacent to each other in young and aged femurs. Data refer to 27 young and 14 aged longitudinal shaved whole-mount cross-sections from 2 young and 2 aged mice. 144 young HSCs and 394 aged HSCs. *p < 0.05. (D) Percentage of HSCs found in clusters (2 cells or more, each 3D image 500 μm × 500 μm for the xy plane and 50 μm along the z plane) in young and aged femora. Imaging data refer to 27 young and 14 aged longitudinal shaved whole-mount cross-sections from 2 young and 2 aged mice. 144 young HSCs and 394 aged HSCs were scored. ****p < 0.0001. (E) Percentage of donor-derived RFP+ young and aged HSCs found in clusters (2 cells or more, each 3D image 500 μm × 500 μm for the xy plane and 50 μm along the z plane) in femora of young recipient mice. Imaging data refer to 8 young and 4 aged longitudinal shaved whole-mount cross-sections from 2 young and 2 aged mice. 75 young HSCs and 108 aged HSCs were scored. ****p < 0.0001. (TIF) [file pbio.2003389.s009.tif]

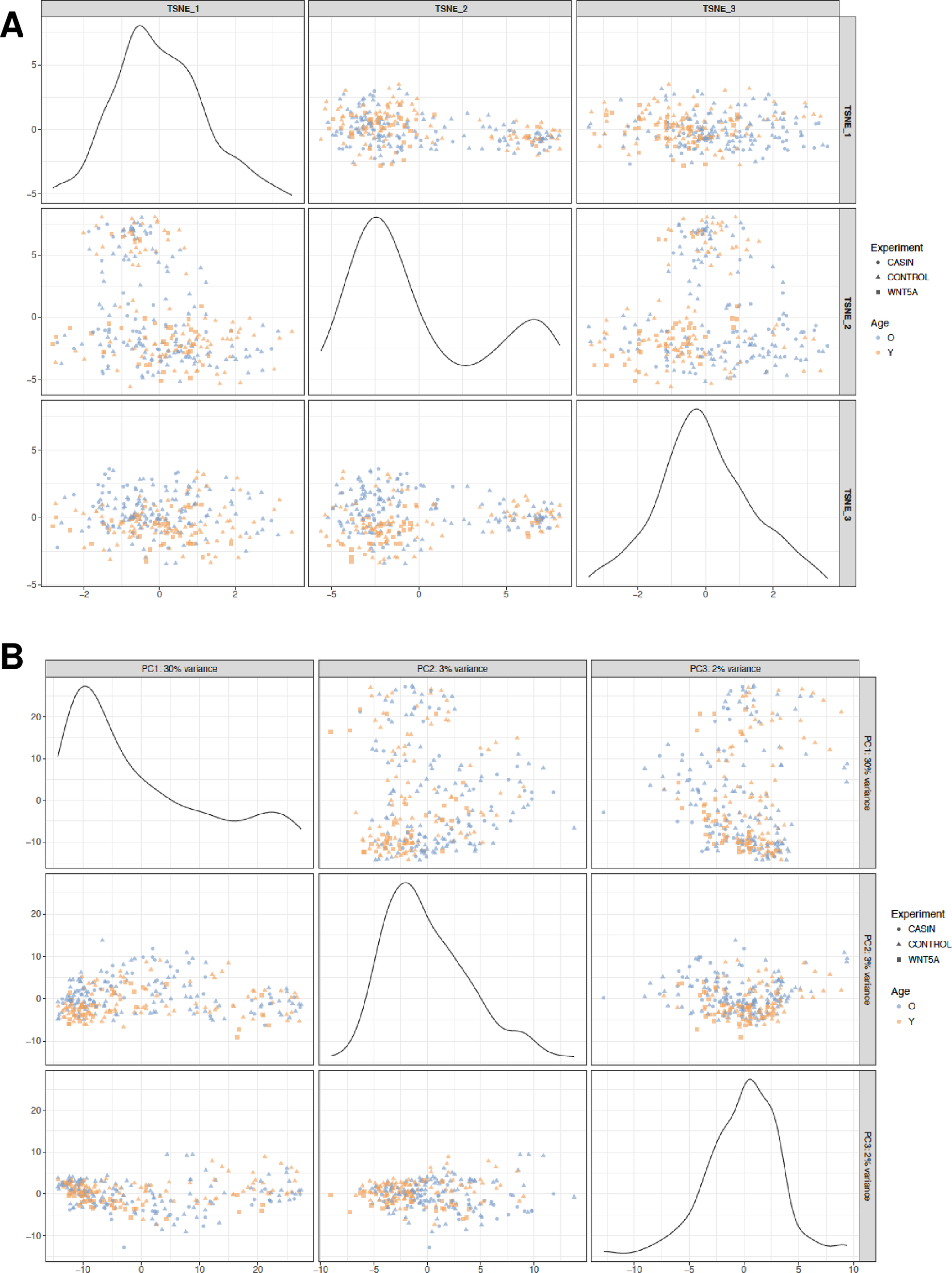

Supplement: S8 Fig — (A) A t-SNE (or TSNE) plot based on the global gene expression profile of all single cells analyzed. Three TSNE components are shown in the matrix plot, where points depict every single cell (O = aged [blue]; Y = young [orange]). Different shapes represent the 3 experimental arms (CASIN = circle; CONTROL = triangle; WNT5A = rectangle). Based on a combination of TSNE_3 with either TSNE_1 or TSNE_2, cells tend to segregate along the age axis. (B) Similarly, 3 PCs generated in a PCA of the global gene expression profile cumulatively explain 35% of the variation in the dataset. Colors represent aged groups (O = aged [blue]; Y = young [orange]), while shapes depict the 3 experimental arms (CASIN = circle; CONTROL = triangle; WNT5A = rectangle). PC, principal component; PCA, principal component analysis; t-SNE, t-Distributed Stochastic Neighbor Embedding. (TIF) [file pbio.2003389.s010.tif]

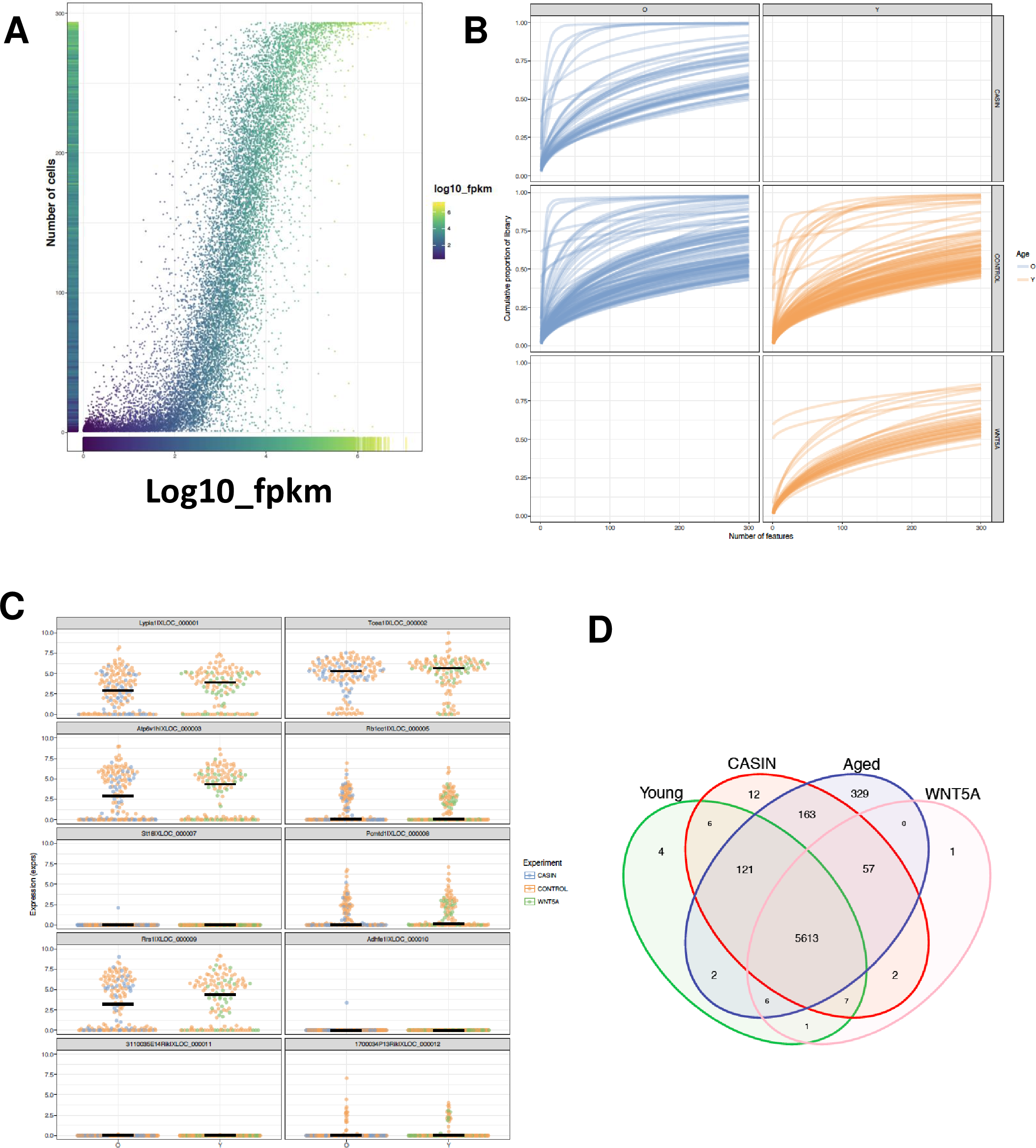

Supplement: S9 Fig — (A) A QC scatter plot showing the relationship between expression (FPKM; in log 10 scale) and the number of cells, i.e., the number of cells expressing a given gene. The relationship basically shows that genes with relatively higher expression normally tend to be expressed by the majority of cells. (B) An overview of the cumulative proportion of each library (global or total expression for a given cell) as a function of the top-expressed features/genes. Plots are shown separately for each experimental arm and age group (aged and CASIN = blue; young and WNT5A = orange). The QC plot shows cell-to-cell difference/variation in distribution of expression. (C) A QC representative plot showing the level of gene expression of some randomly selected genes. The y-axis shows expression levels (log scale), while the x-axis shows the age group. Cells are represented by dots in the plot, and the colors reflect the 3 experimental arms (CASIN = blue; CONTROL = orange; WNT5A = green). (D) Venn diagram showing the overlap between the 4 experimental arms based on genes down-regulated in young and aged CASIN-treated HSCs. FPKM, Fragments Per Kilobase Million; QC, quality control. (TIF) [file pbio.2003389.s011.tif]

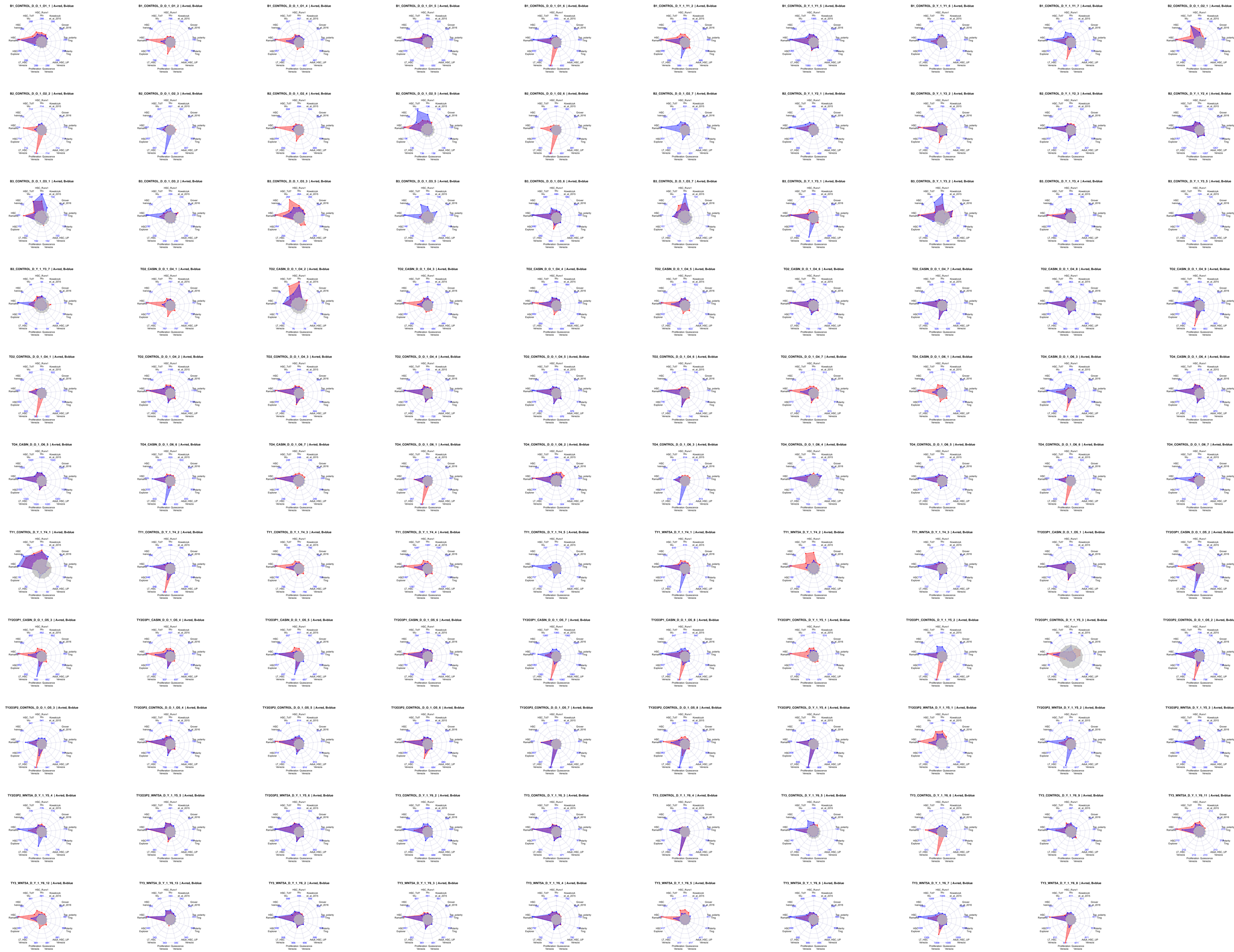

Supplement: S11 Fig — We simultaneously interrogated 13 previously published hematopoietic stem cell and polarity signatures, and each daughter cell pair was depicted in a single radar plot where vertices in the plot correspond to each of the considered gene set. Pairs were then compared for significance of similarity/dissimilarity by a goodness of fit test across all significant gene sets. (PDF) [file pbio.2003389.s013.pdf]

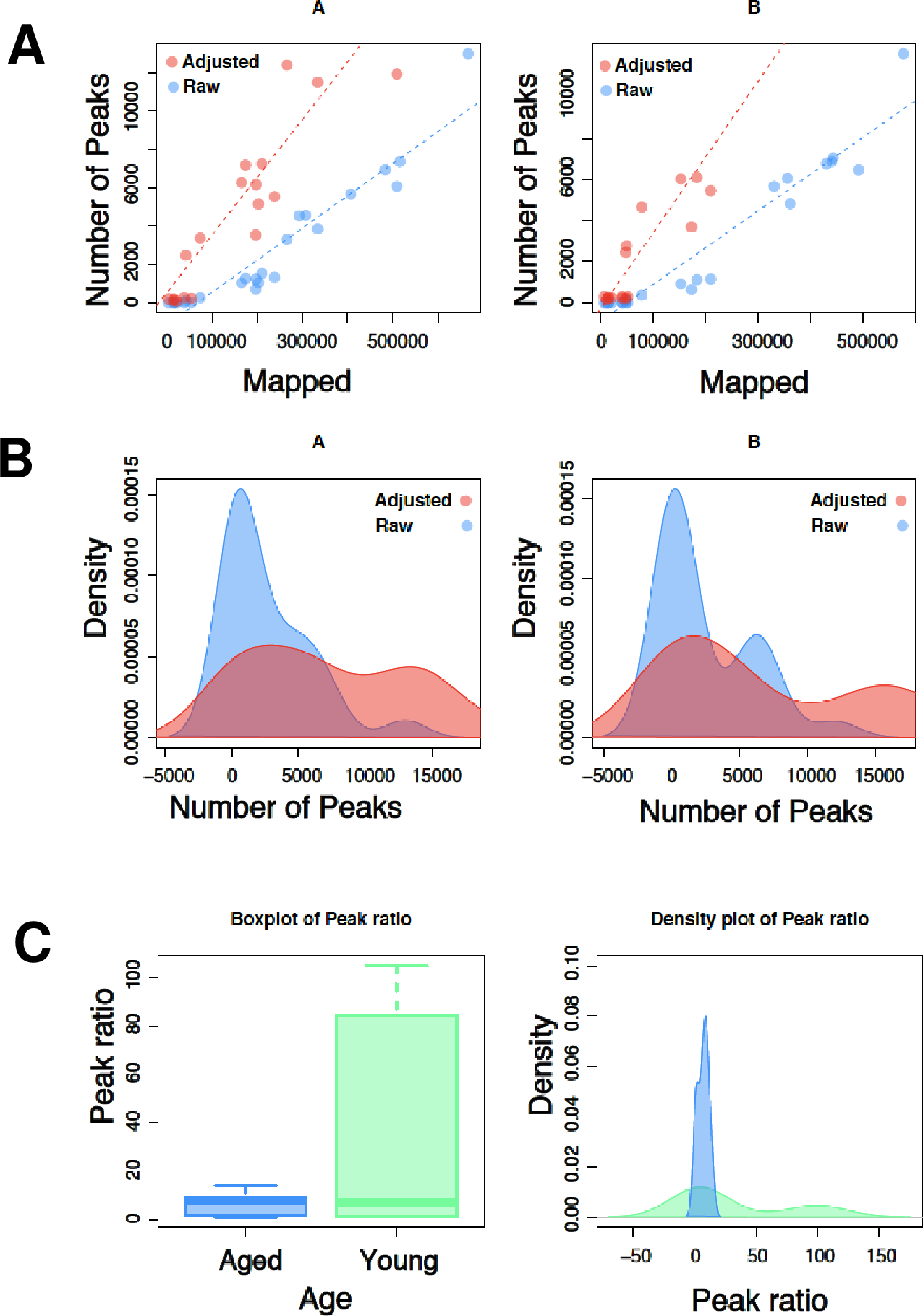

Supplement: S12 Fig — (A) Representative plots presenting the relationship between number of mapped reads (x-axis) and number of peaks called (y-axis) across pairs A (left panel) and B (right panel). Blue points represent the observed peak counts (raw), while red points depict the number of peaks per million reads (adjusted). (B) Data identical to panel A are shown in a density plot where the x-axis represents peak counts and the y-axis shows density/frequency. Blue and red polygons represent the raw and adjusted peak counts, respectively. (C) The ratio of the peak number of paired daughters from young (green) and aged (blue) HSCs. A ratio of one represents an equal number of peaks in paired daughter cells. Daughter pairs of young HSCs present with higher ratios than daughter pairs from aged HSCs. The right panel shows the overall distribution of the ratio of the peaks for paired daughters of young and aged HSCs. (TIF) [file pbio.2003389.s014.tif]

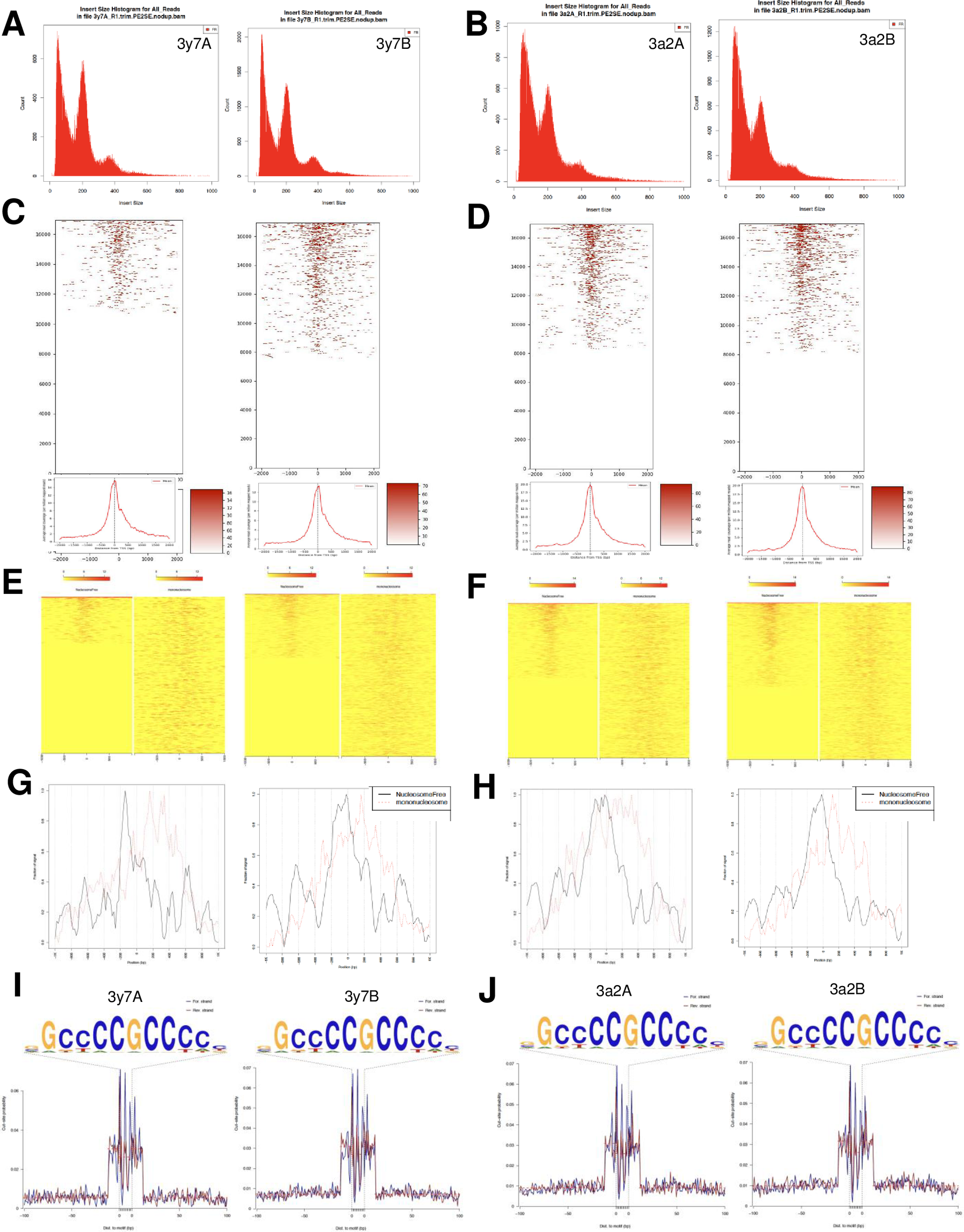

Supplement: S13 Fig — (A, B) Relationship between fragment length and normalized read density for 2 selected pairs (A = young pair 3y7A/3y7B; B = aged pair 3a2A/3a2B). The plot shows the characteristic distribution for ATAC-seq, mainly a large number of reads less than 100 bp and multiple periodicity. (C, D) Heatmaps showing an enrichment of the signal of the nucleosome-free fraction around active TSSs of selected cell pairs (C = young pair 3y7A/3y7B; D = aged pair 3a2A/3a2B). Each heatmap is accompanied by a bottom panel showing the mean distribution of the read coverage where the x-axis shows the distance from the TSS (in base pairs) and the y-axis shows the average read coverage per million mapped reads. (E, F) Comparative heatmap of the nucleosome-free and the mononucleosome signal across all active TSSs for representative pairs (E = young pair 3y7A/3y7B; F = aged pair 3a2A/3a2B). Nucleosome-free fragments, as presented also in panels C and D, show a unimodal enrichment around TSSs, while nucleosome bound fragments show phased enrichment both upstream and downstream of the active TSSs. (G, H) Line plots showing the same data as in panels E and F, where the x-axis shows the position around the TSSs and the y-axis represents the fraction of the signal. Full black lines depict nucleosome-free signals, while dashed red lines show nucleosome bound (mononucleosome) signals. (I, J) Genome-wide occupancy (ATAC-seq footprint) of a selected TF in representative daughter cell pairs (I = young pair 3y7A/3y7B; J = aged pair 3a2A/3a2B). The x-axis shows the distance to the motif of the TF (shown in the inset base logos), and the y-axis indicates the transposase cut-site probability. Forward and reverse strand signals are shown in blue and red lines, respectively. (TIF) [file pbio.2003389.s015.tif]

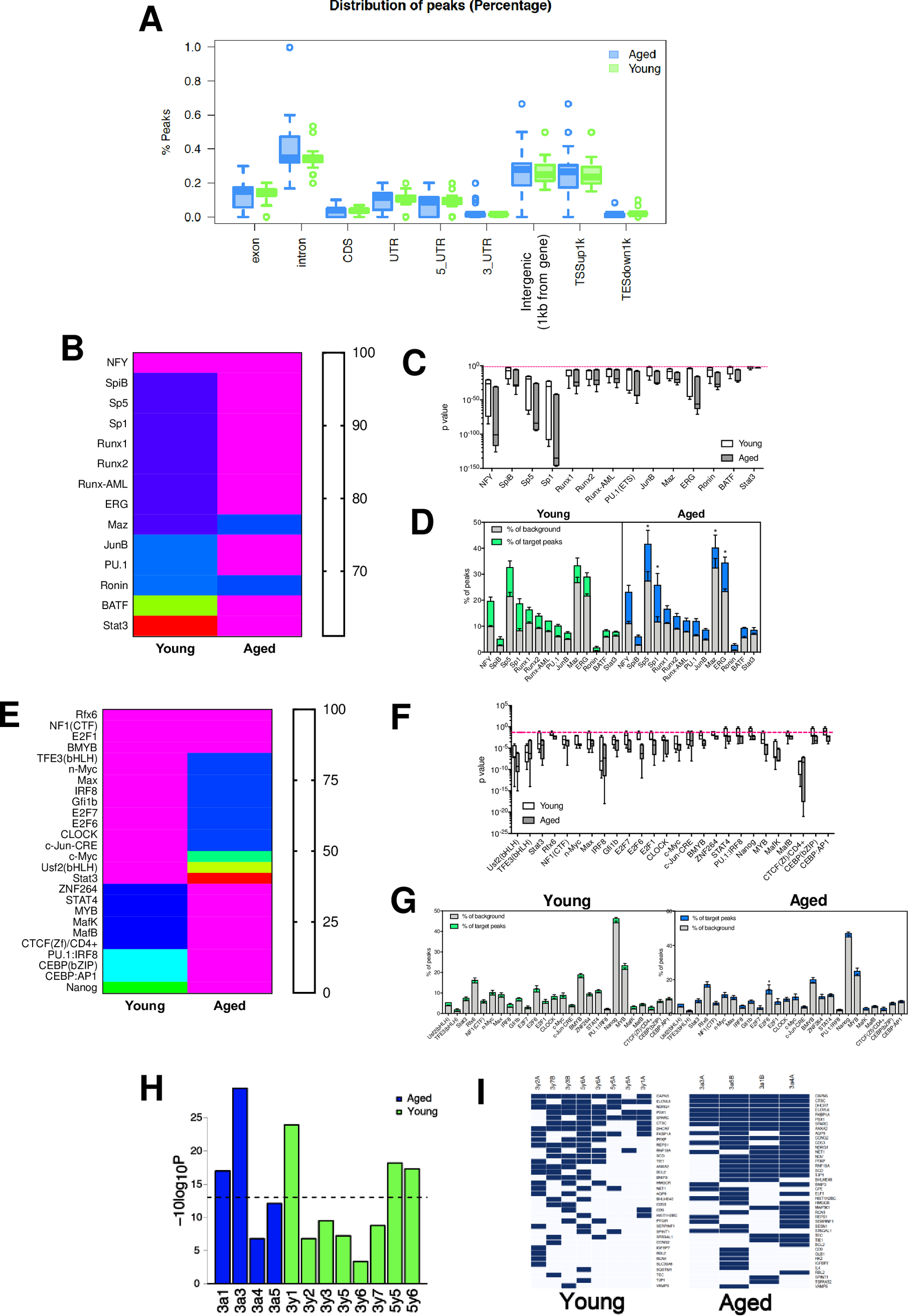

Supplement: S14 Fig — (A) Percentage of the peaks assigned to distinct genomic regions. ATAC-seq peaks were primarily aligned to introns, intergenic regions 1 kb apart from the gene and at TSSs. There was no difference in the distribution of peaks between young and aged daughter cells. (B) Heatmap depicting the frequency of daughter stem cells showing a significant enrichment for specific TFs (the TFs most common to all pairs were selected). TFs were ranked (descending) based on the frequency of their peak count in daughter cells from young HSCs. (C, D) p-Value (panel C) and percentage of target sequences with a significant motif enrichment and of background sequences (panel D) for each of the TFs depicted in panel B. *Significantly (p < 0.05) different percentage of target sequences with a significant motif enrichment. (E) Heatmap depicting the frequency of daughter progenitor cells showing a significant enrichment for specific TFs (the TFs most common to all pairs were selected). TFs were ranked (descending) based on the frequency of their peak count in young daughter stem cells. (F, G) p-Value (panel F) and percentage of target sequences with a significant motif enrichment and of background sequences (panel G) for each of the TFs depicted in panel E. (H) Graph showing the logP values for the GSEA for genes included in a previously published differentiation/proliferation gene set (BRUNO_HEMATOPOIESIS). This gene set includes genes that changed expression when multipotent cells underwent differentiation and thus lost self-renewal and proliferation properties. Only 2 aged and 3 young daughter progenitor cells showed a significant enrichment. Daughter cells were grouped as progenitor cells according to Fig 8F. (I) Heatmap showing genes that are common between a published differentiation/proliferation gene set (GSEA for BRUNO_HEMATOPOIESIS) and the annotated peaks of the daughter cell with a higher peak count in a given pair (such daughter cells were scored as progenitor cells according to [file pbio.2003389.s016.tif]

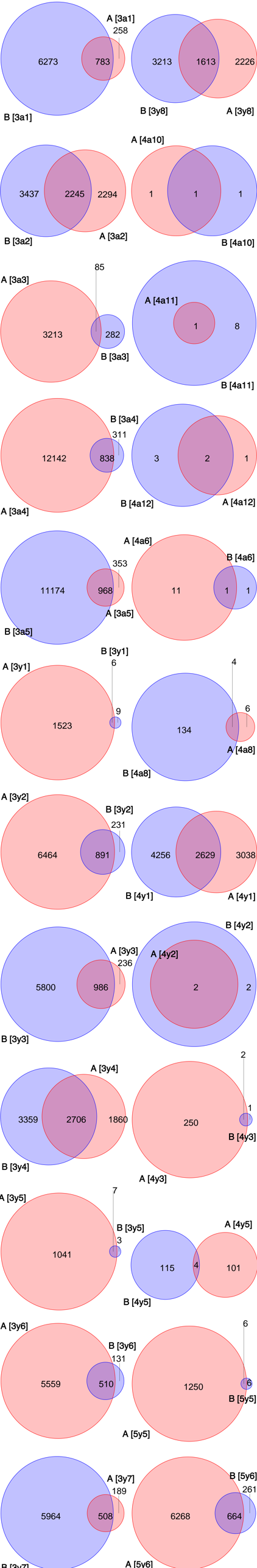

Supplement: S15 Fig — (PDF) [file pbio.2003389.s017.pdf]

A [3a1]

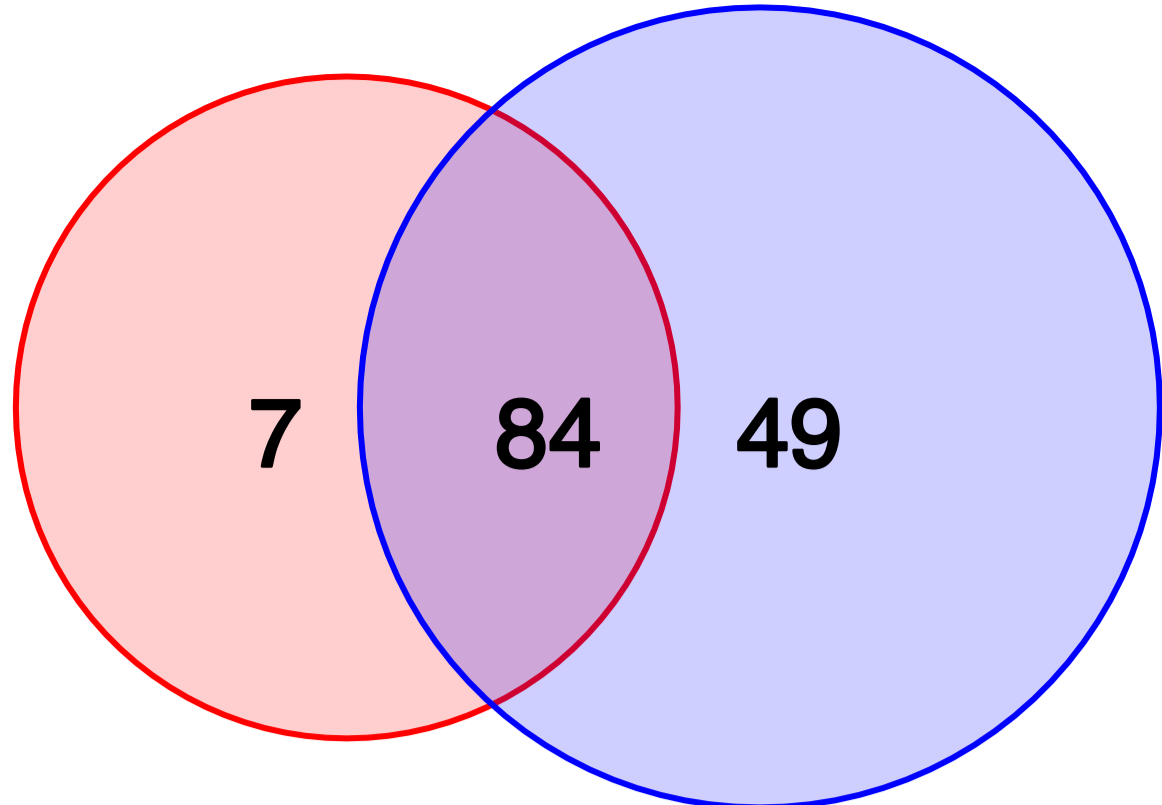

B [3a1]

A [3a2]

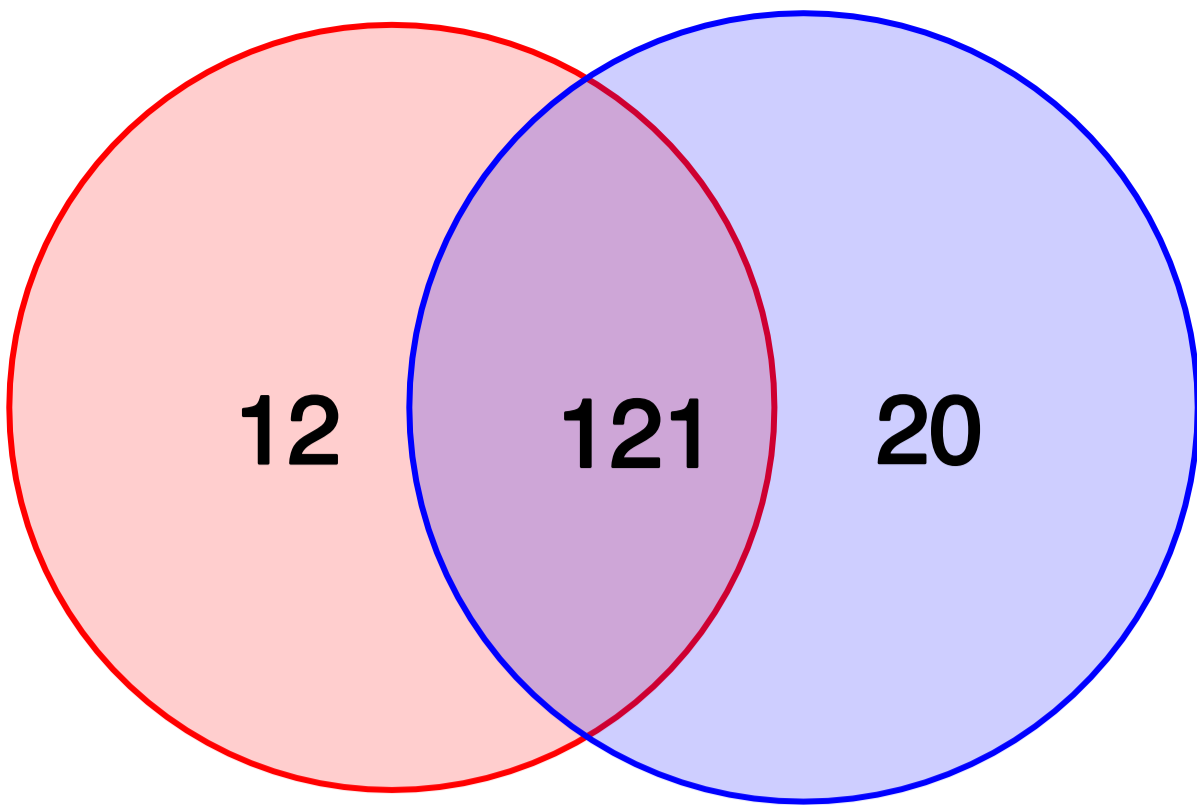

B [3a2]

A [3a3]

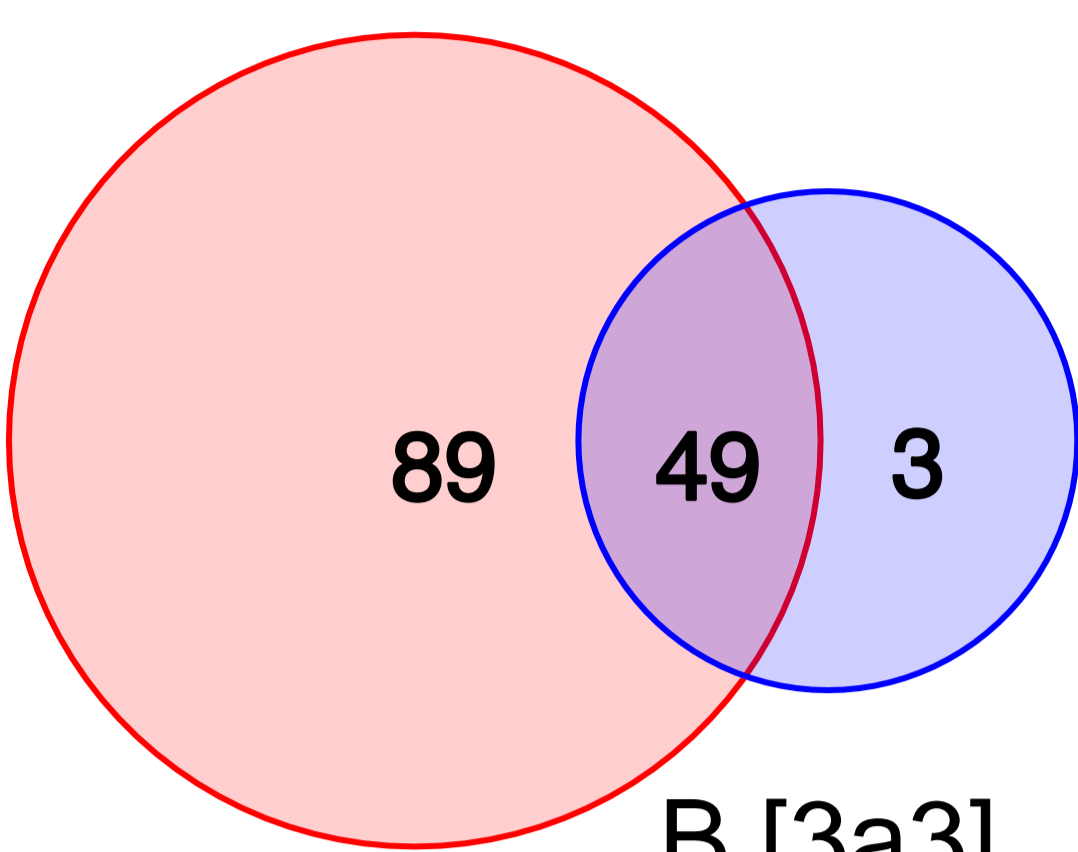

B [3a3]

A [3a4]

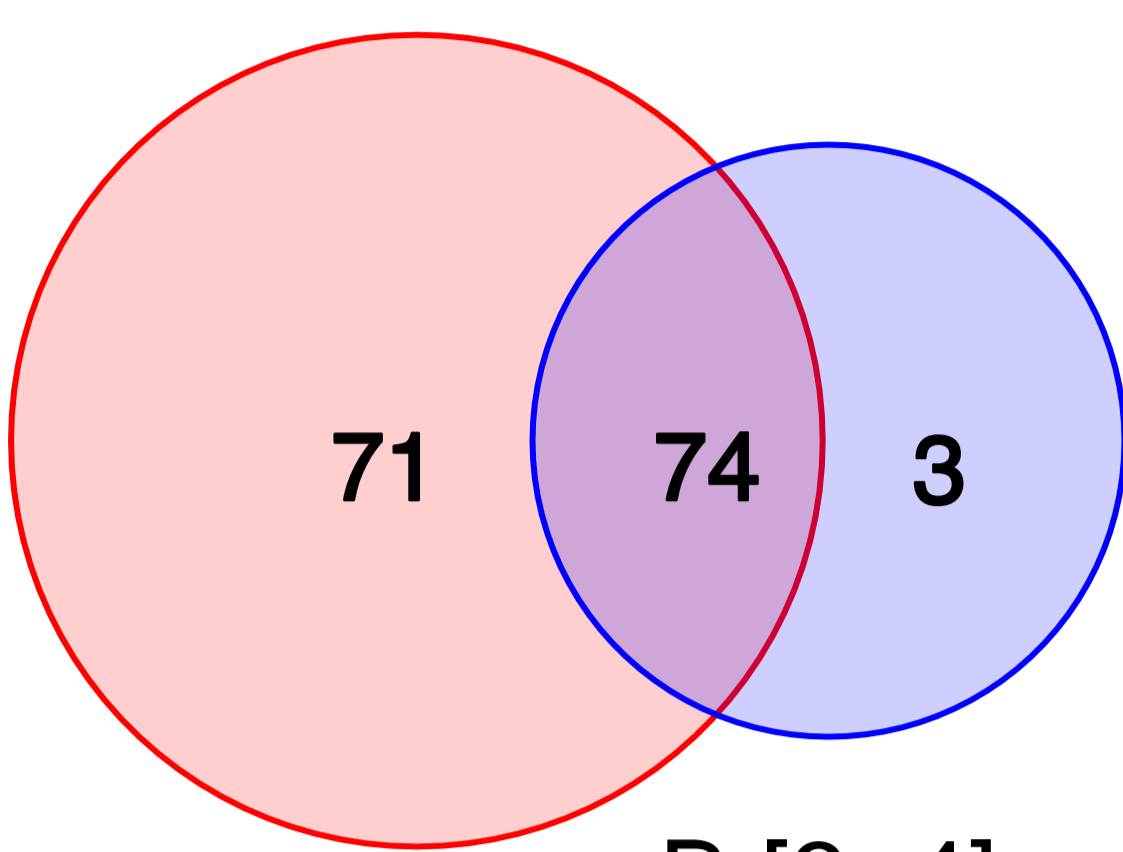

B [3a4]

A [3a5]

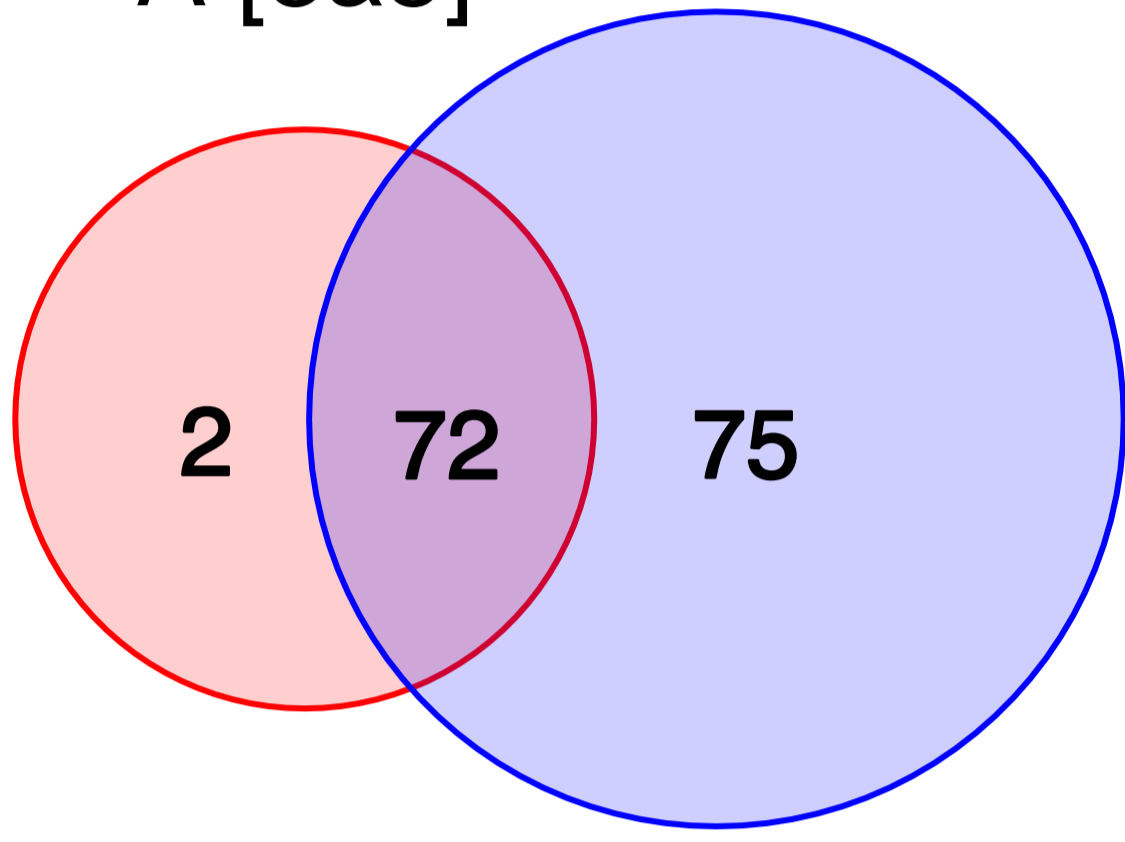

B [3a5]

A [3y2]

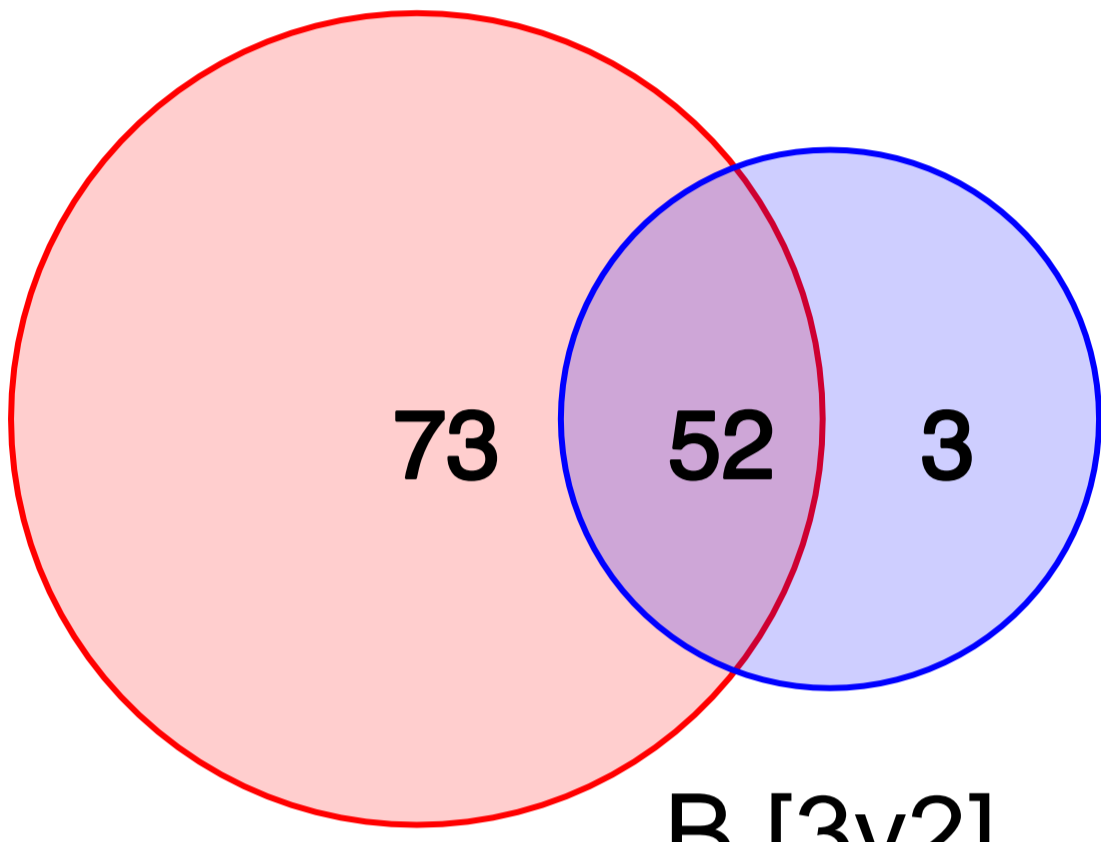

B [3y2]

A [3y3]

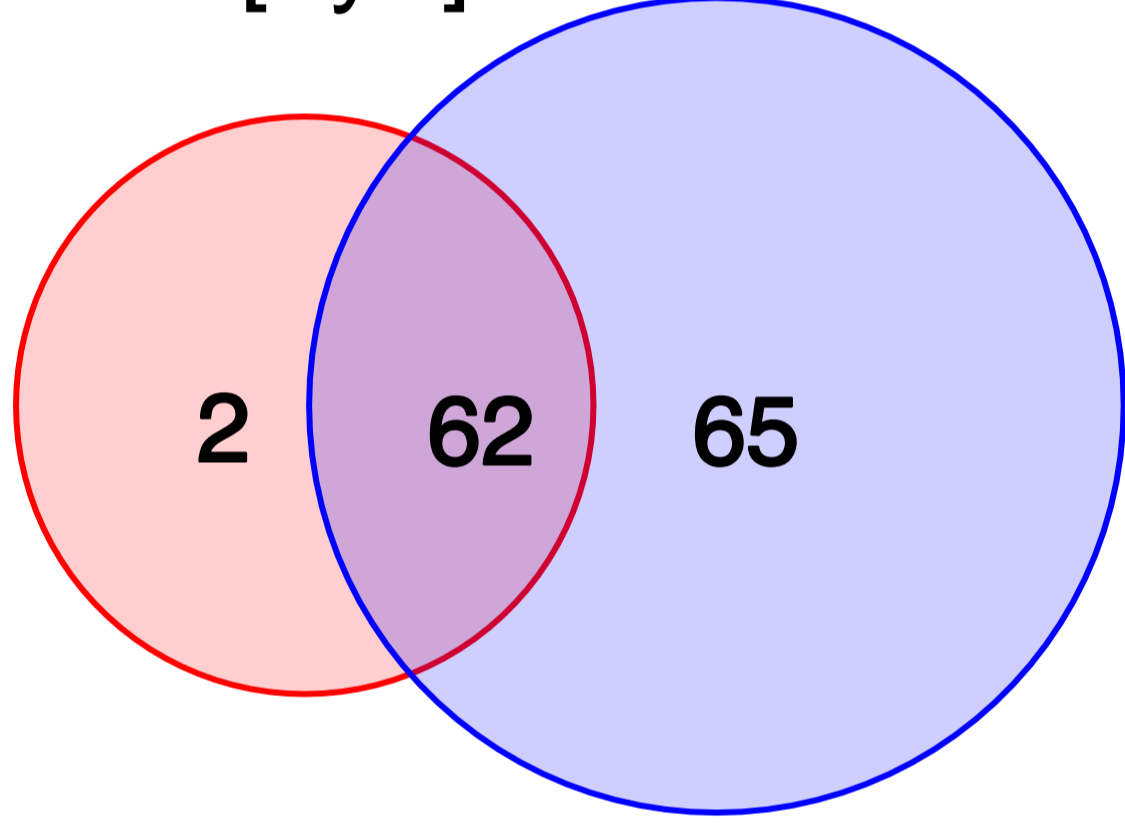

B [3y3]

A [3y4]

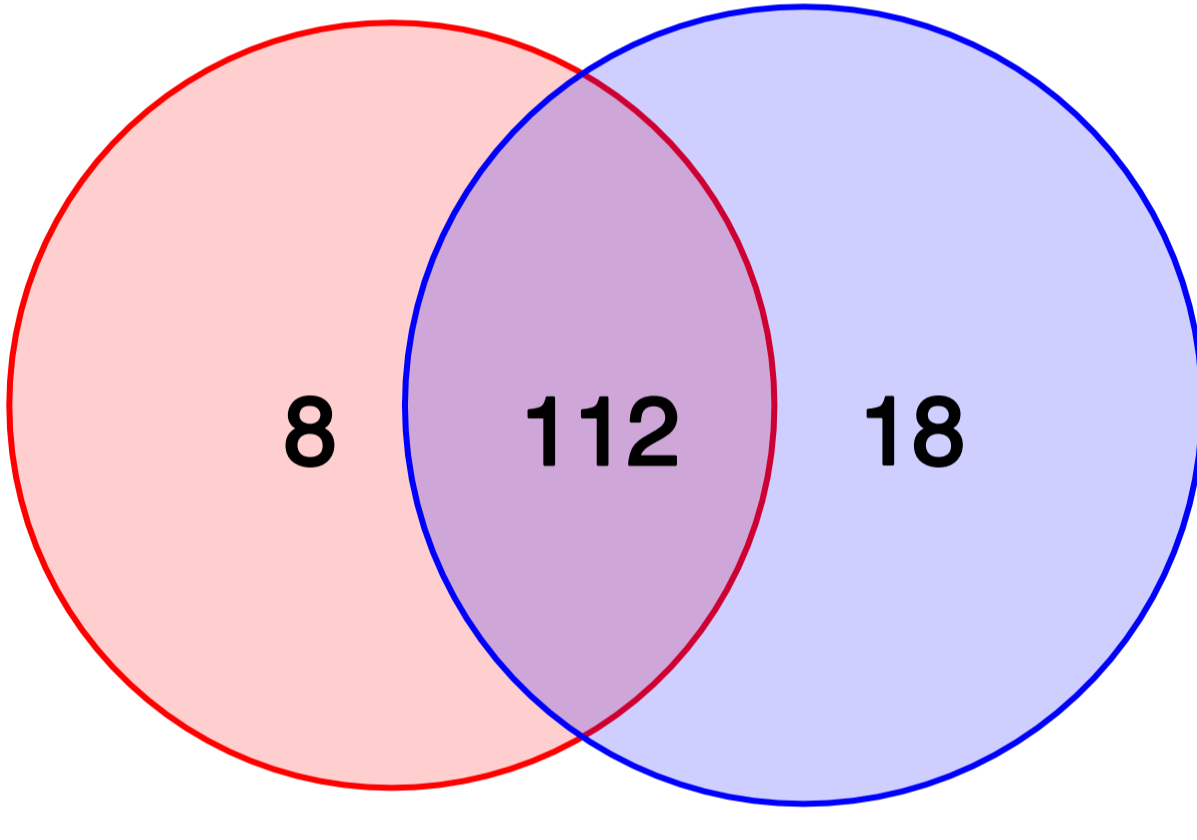

B [3y4]

A [3y6]

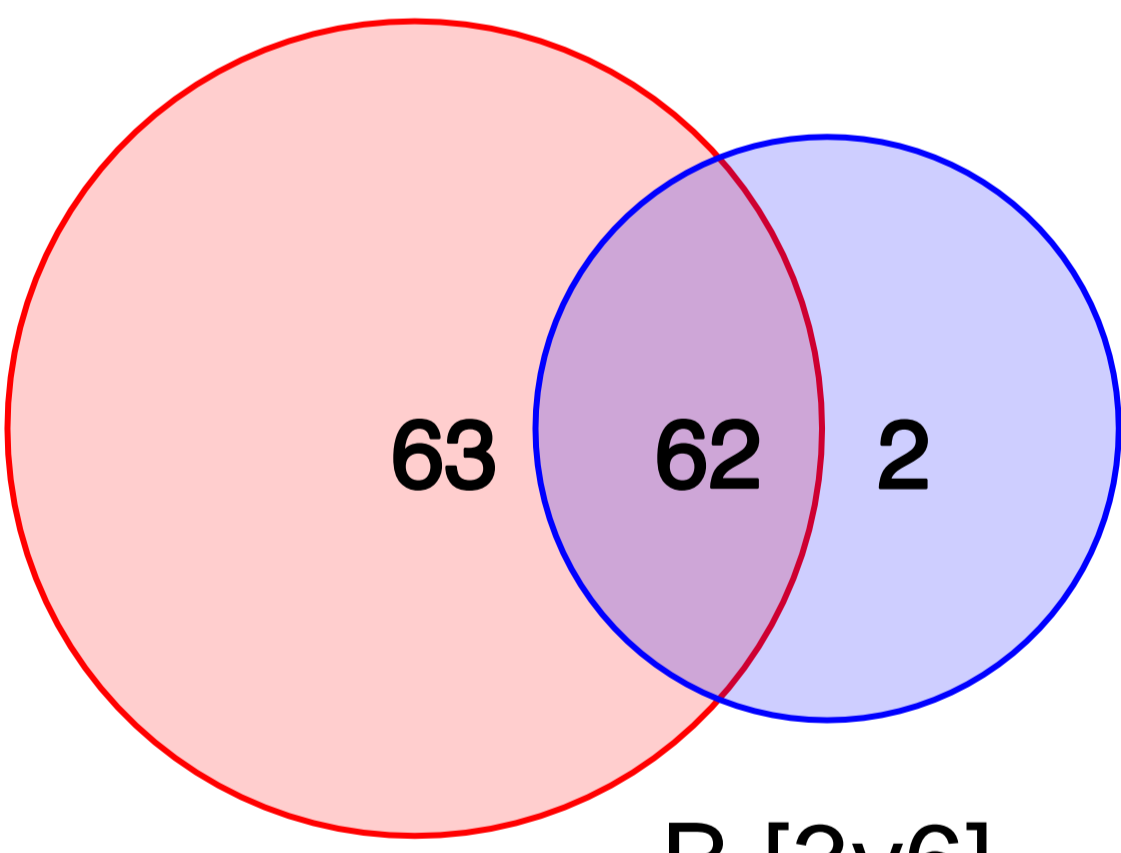

B [3y6]

A [3y7]

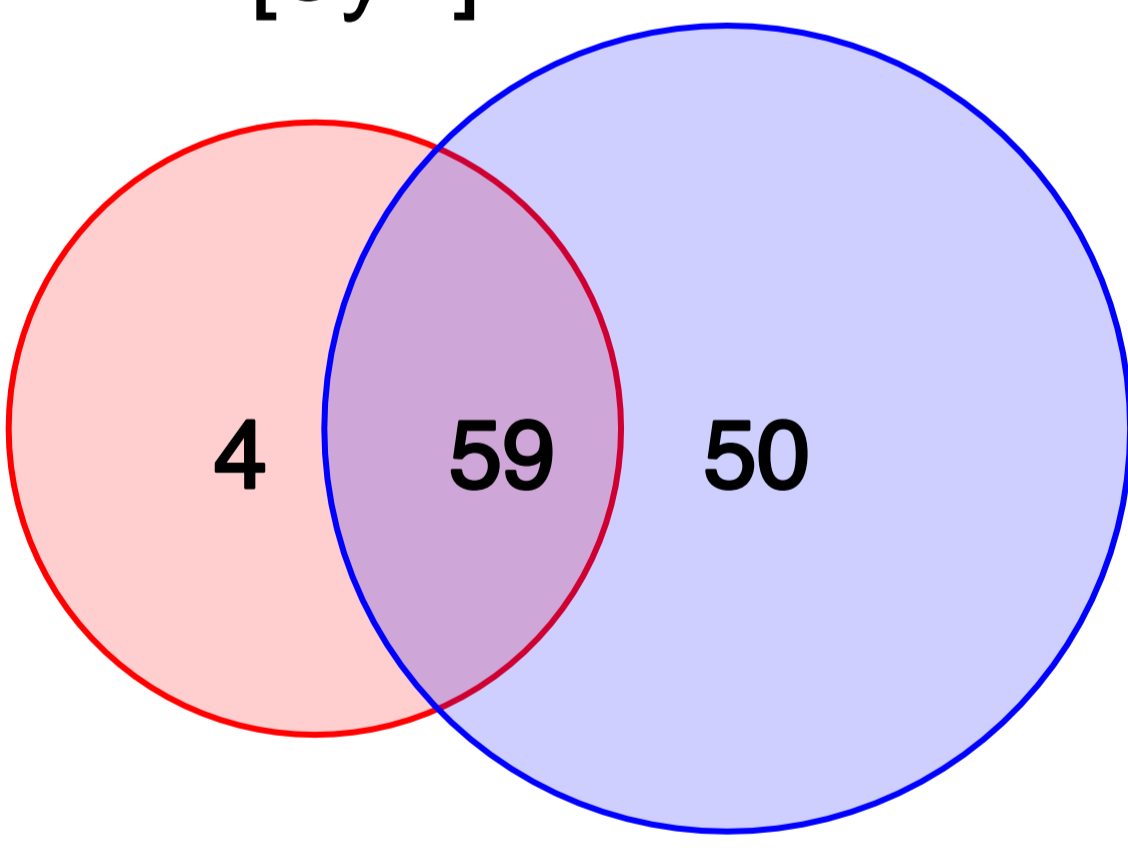

B [3y7]

A [3y8]

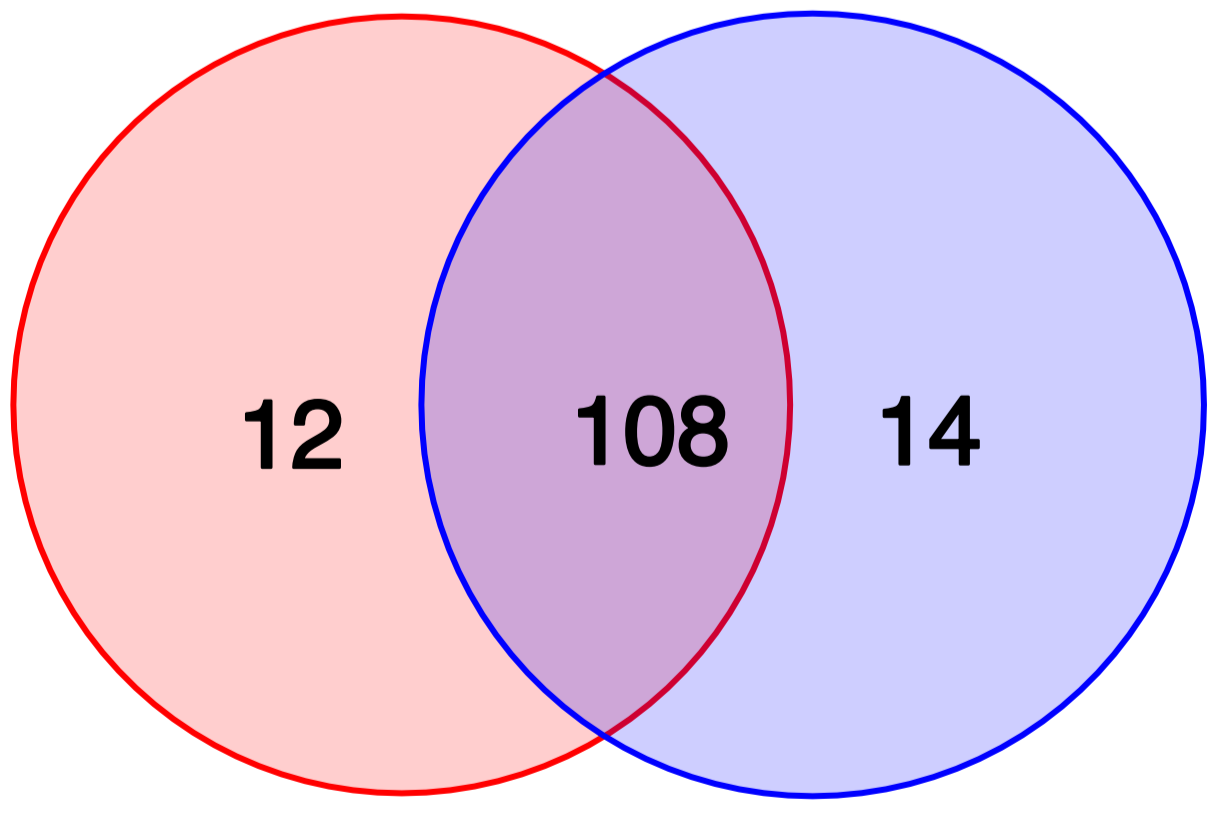

B [3y8]

A [4y1]

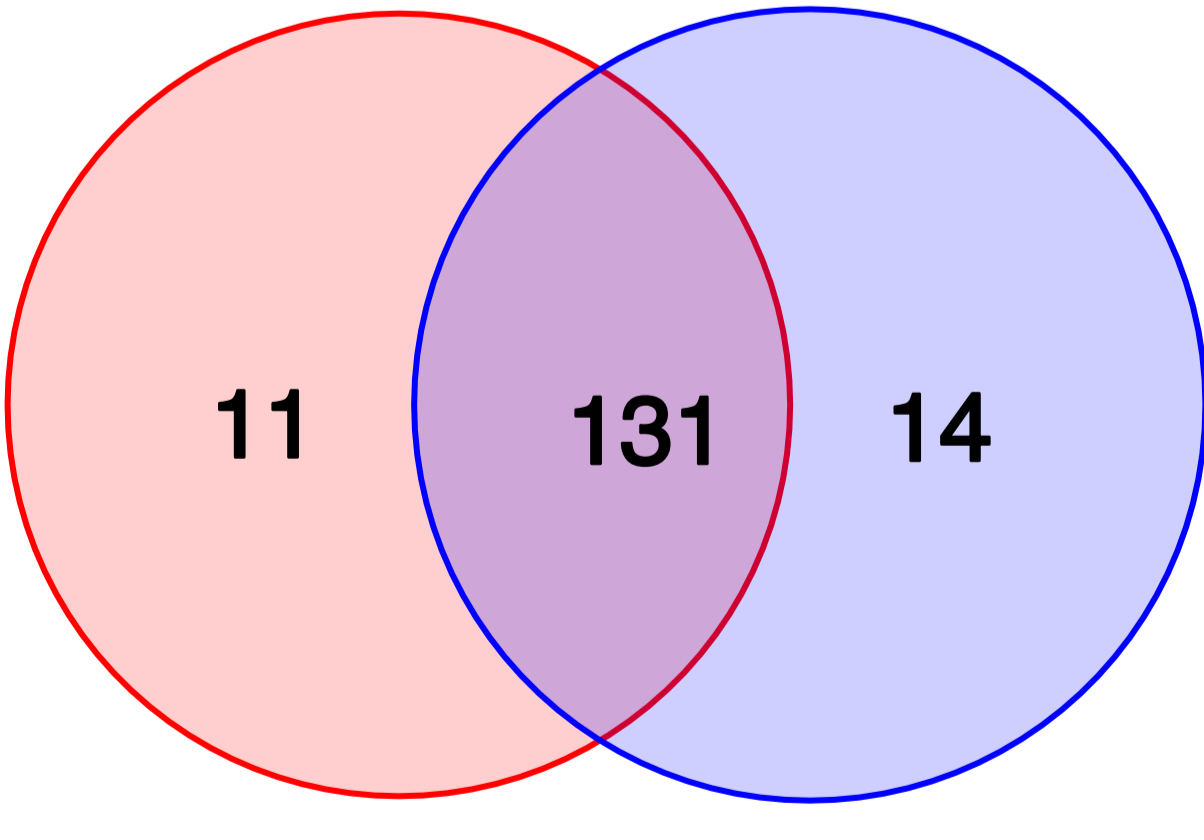

B [4y1]

A [4y5]

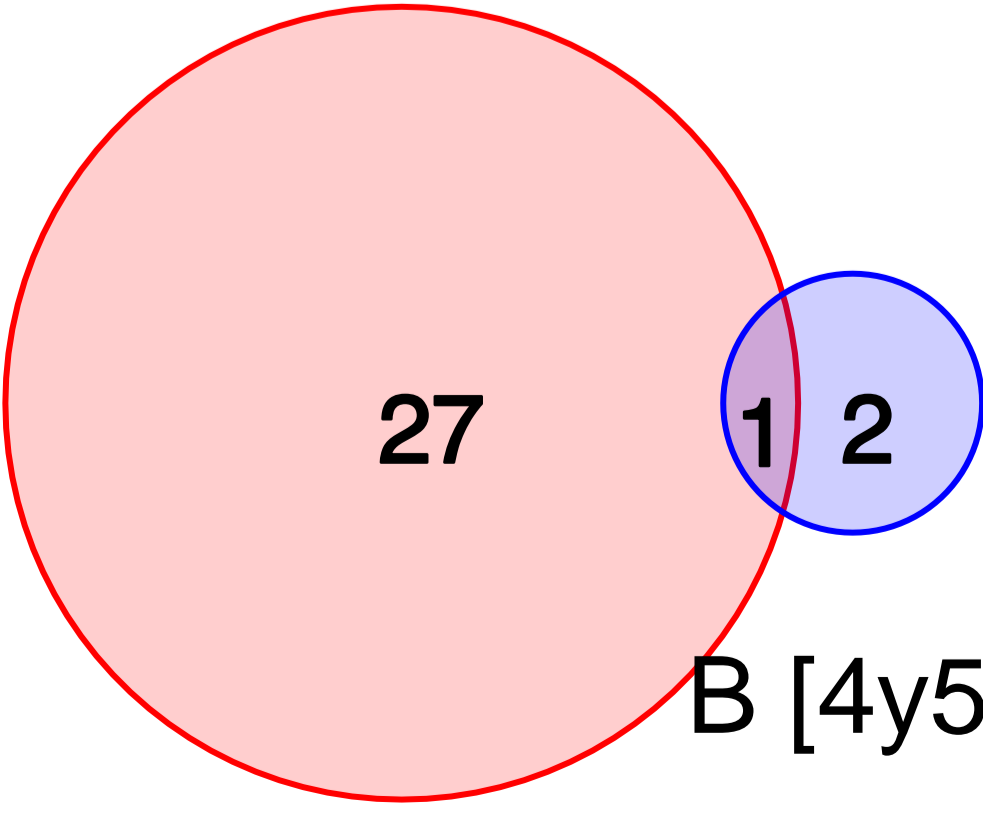

B [4y5]

A [5y6]

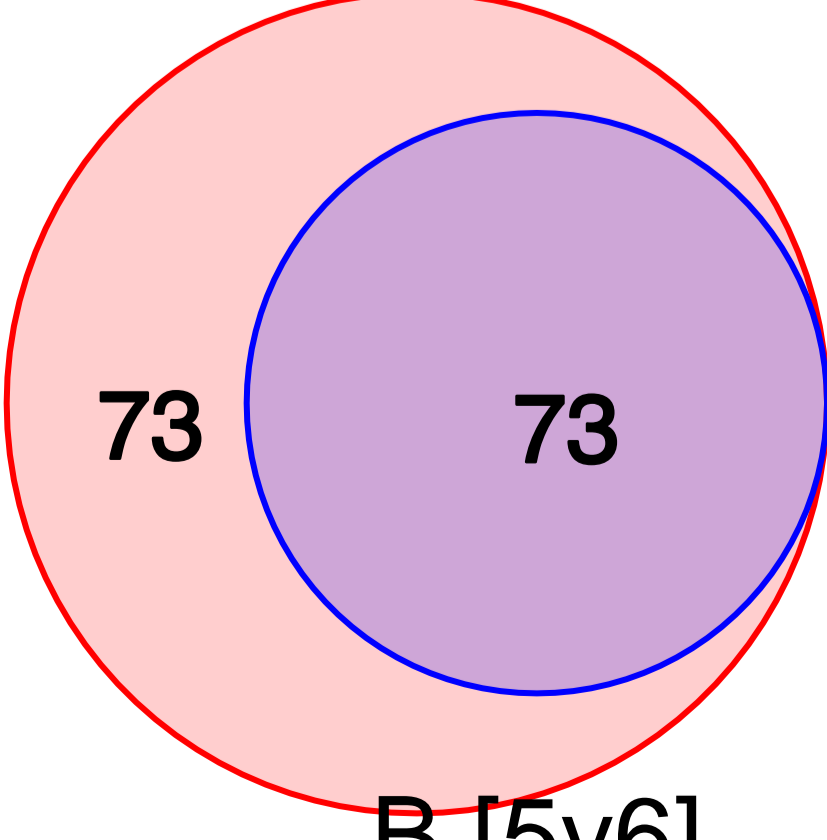

B [5y6]

Supplement: S17 Fig — (PDF) [file pbio.2003389.s019.pdf]
