# Supplementary material for: Aging alters the epigenetic asymmetry of HSC division
Source: PLoS Biol. 2018 Sep 20;16(9):e2003389. doi: 10.1371/journal.pbio.2003389 (PMC6168157; doi:10.1371/journal.pbio.2003389)
Supplement: S2 Fig — (PDF) [file pbio.2003389.s004.pdf]

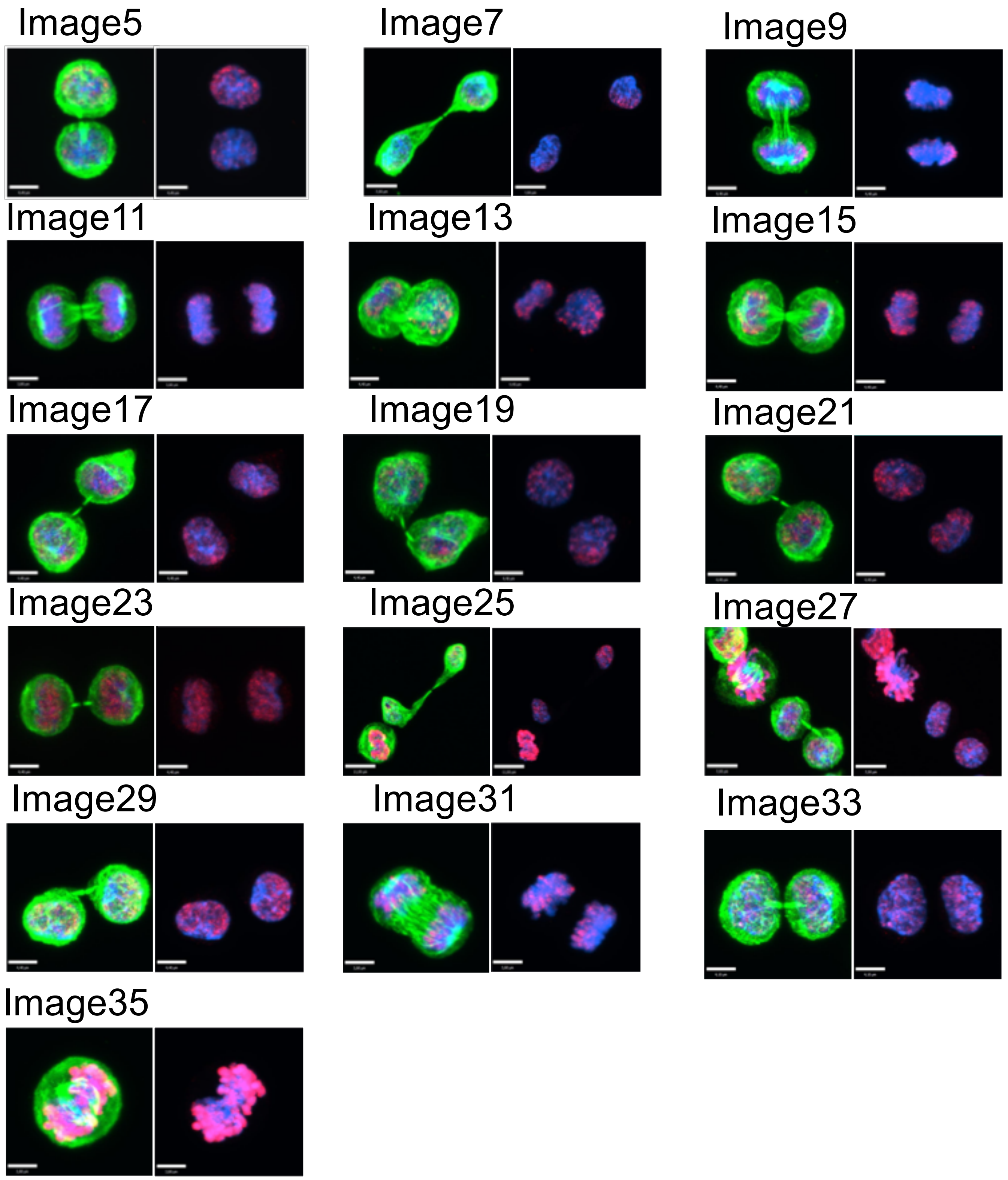

Young ctr; acH4k16 (red), tubulin (green), DAPI (blue); exp#1

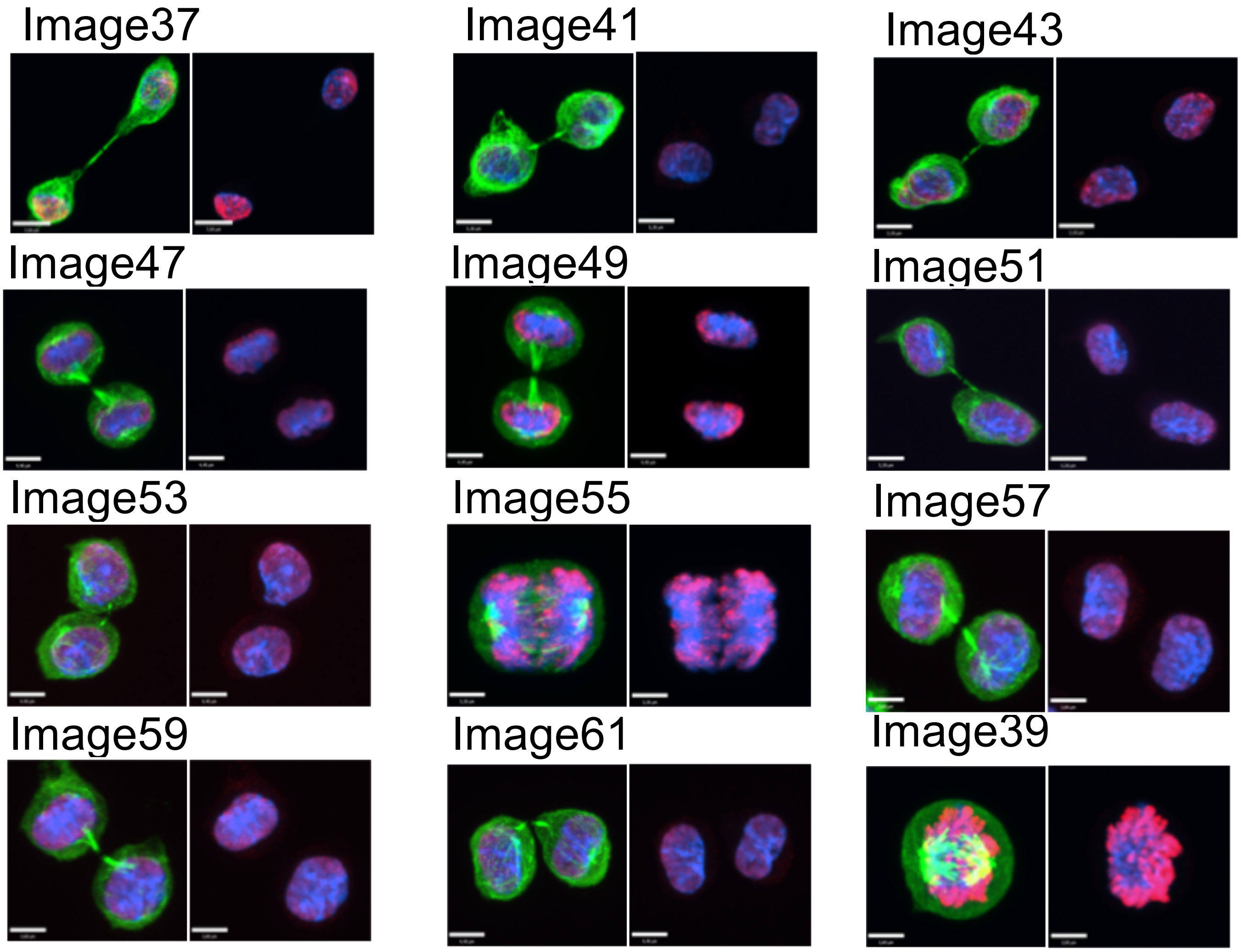

Aged ctr; acH4k16 (red), tubulin (green), DAPI (blue); exp#1

Image63

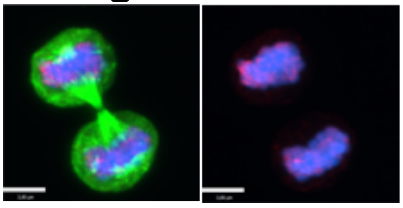

Image65

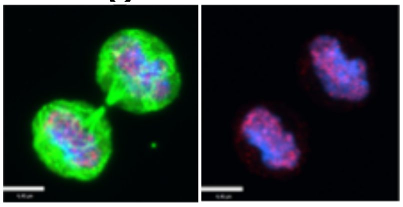

Image67

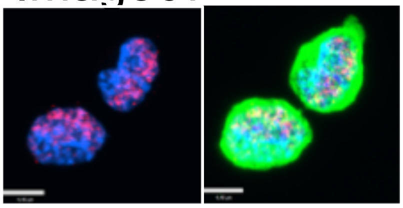

Image69

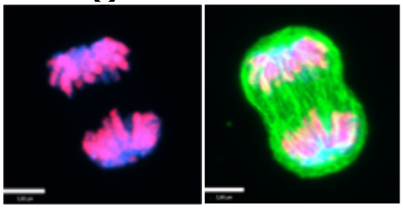

Image71

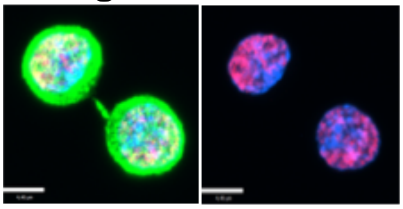

Image73

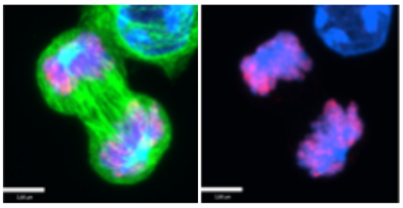

Image75

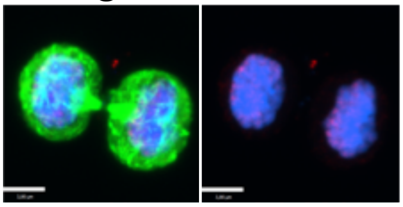

Image77

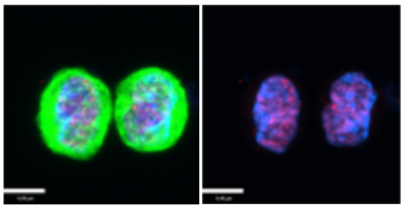

Image79

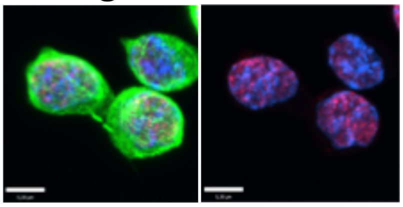

Young+Wnt5a 100ng/ml; acH4k16 (red), tubulin (green), DAPI (blue);
exp#1

Image83

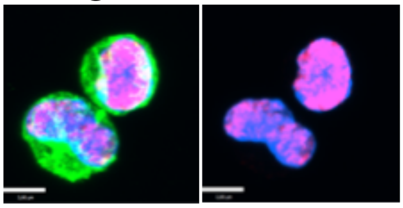

Image85

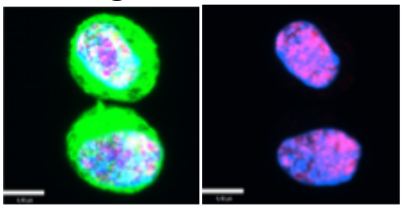

Image87

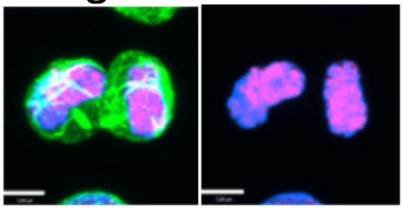

Image89

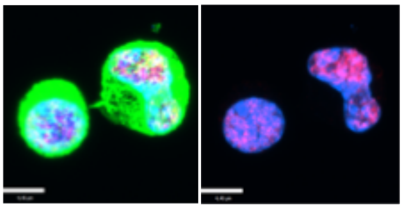

Image91

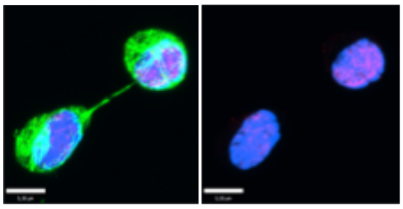

Aged+CASIN5μM; acH4k16 (red), tubulin (green), DAPI (blue);
exp#1

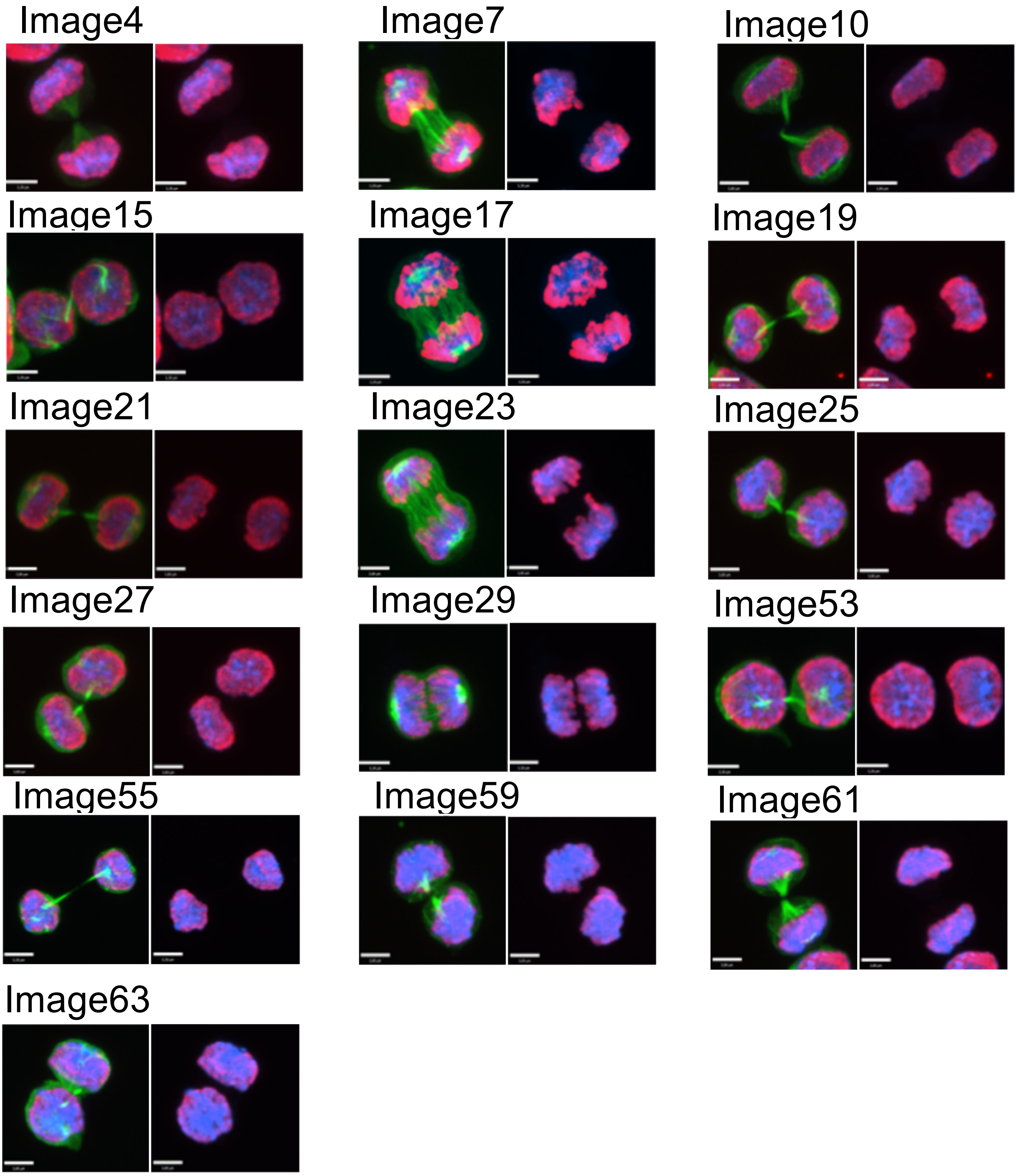

Young ctr; acH4k16 (red), tubulin (green), DAPI (blue); exp#2

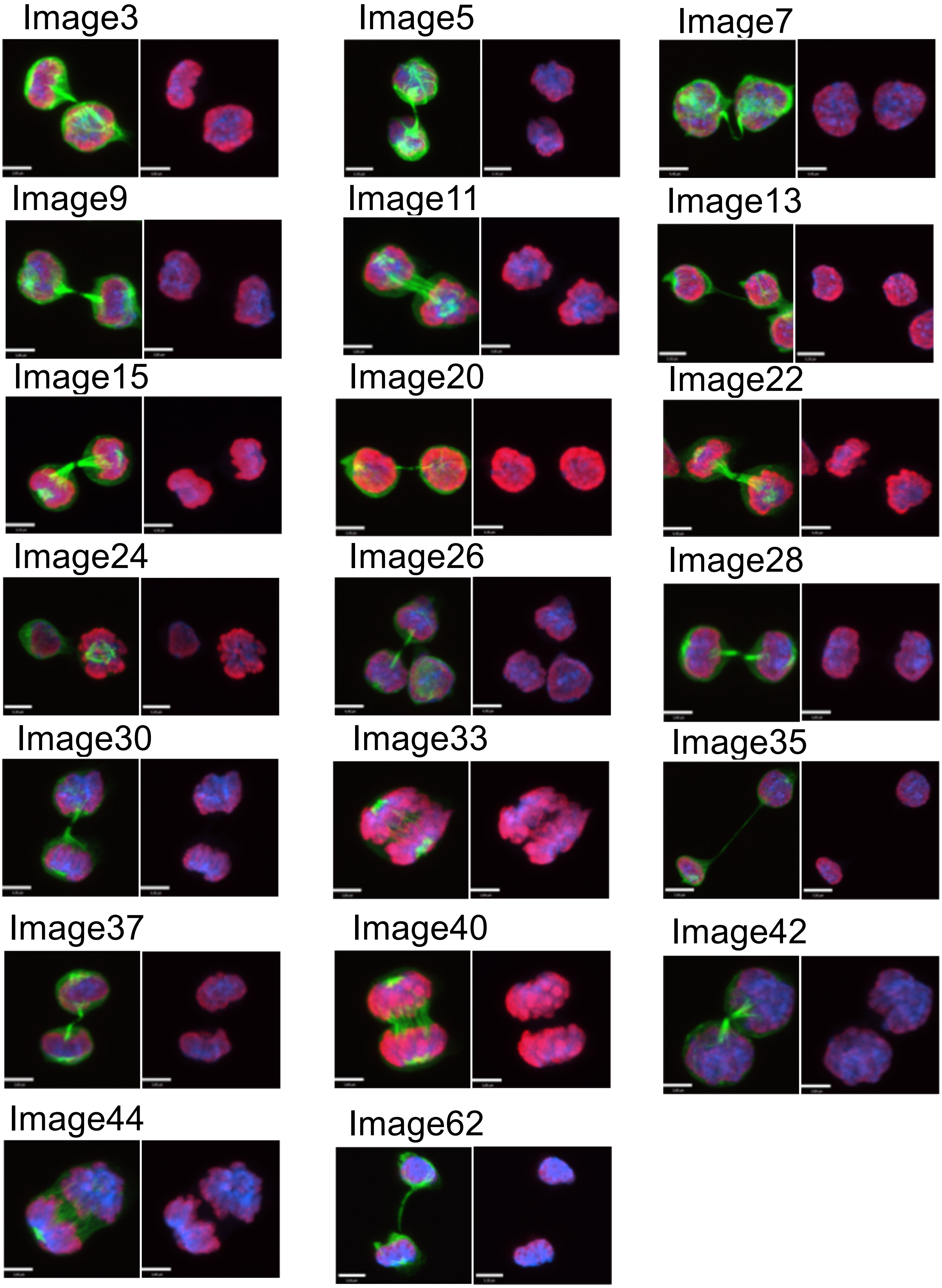

Aged ctr; acH4k16 (red), tubulin (green), DAPI (blue); exp#2

Image2

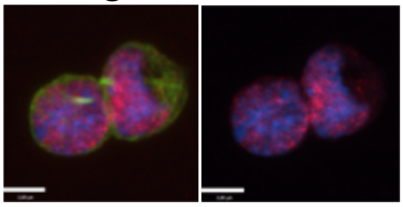

Image4

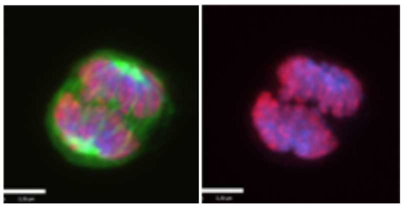

Image7

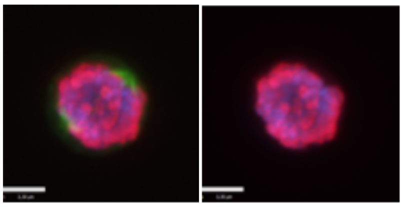

Image9

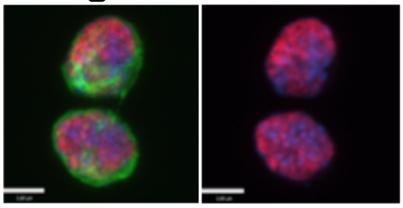

Image11

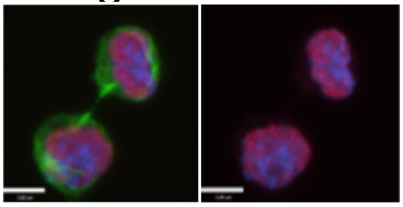

Image13

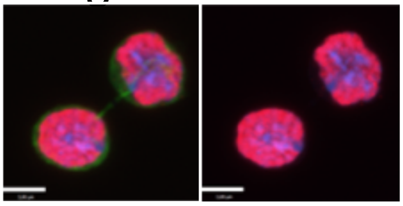

Image15

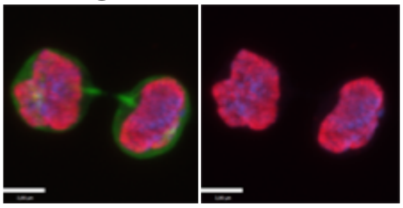

Image19

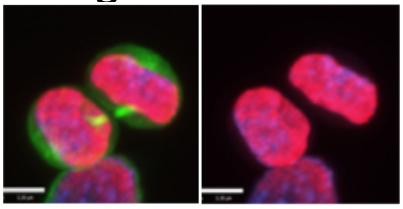

Image21

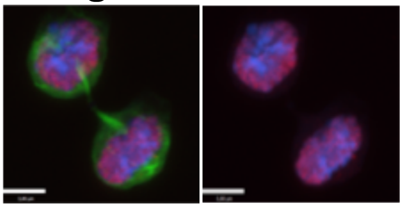

Image27

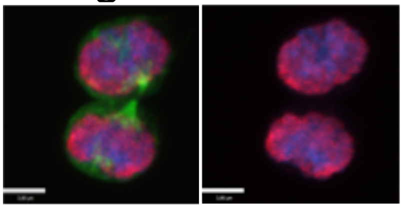

Image29

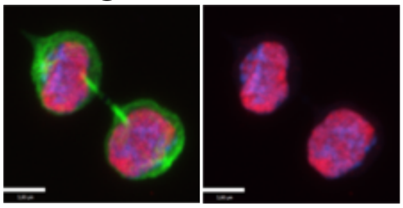

Image31

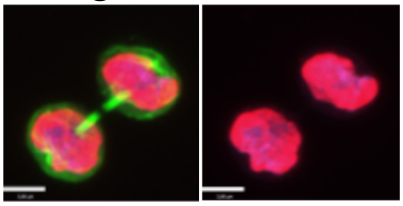

Image47

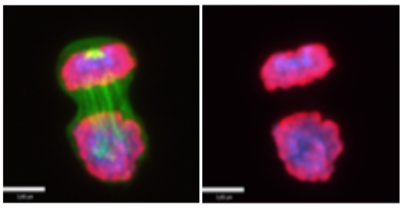

Image49

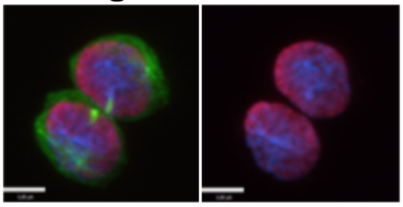

Image51

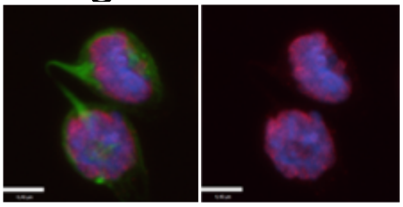

Image53

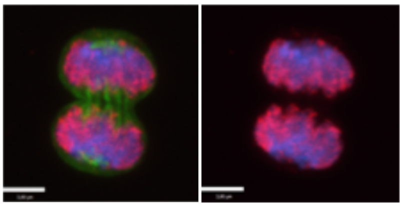

Image55

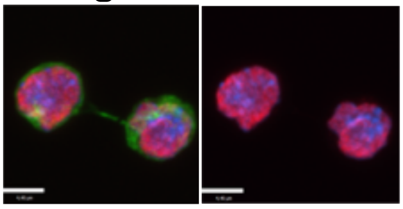

Image57

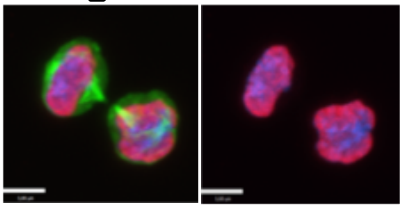

Image59

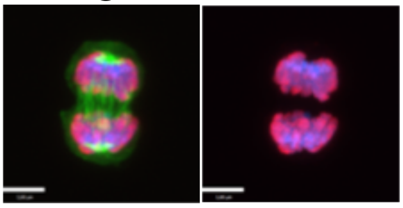

Image61

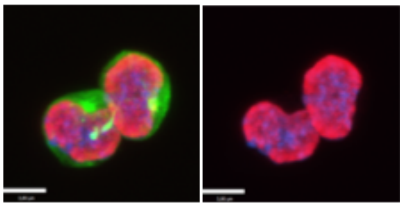

Aged+CASIN5µM; acH4k16 (red), tubulin (green), DAPI (blue);
exp#2

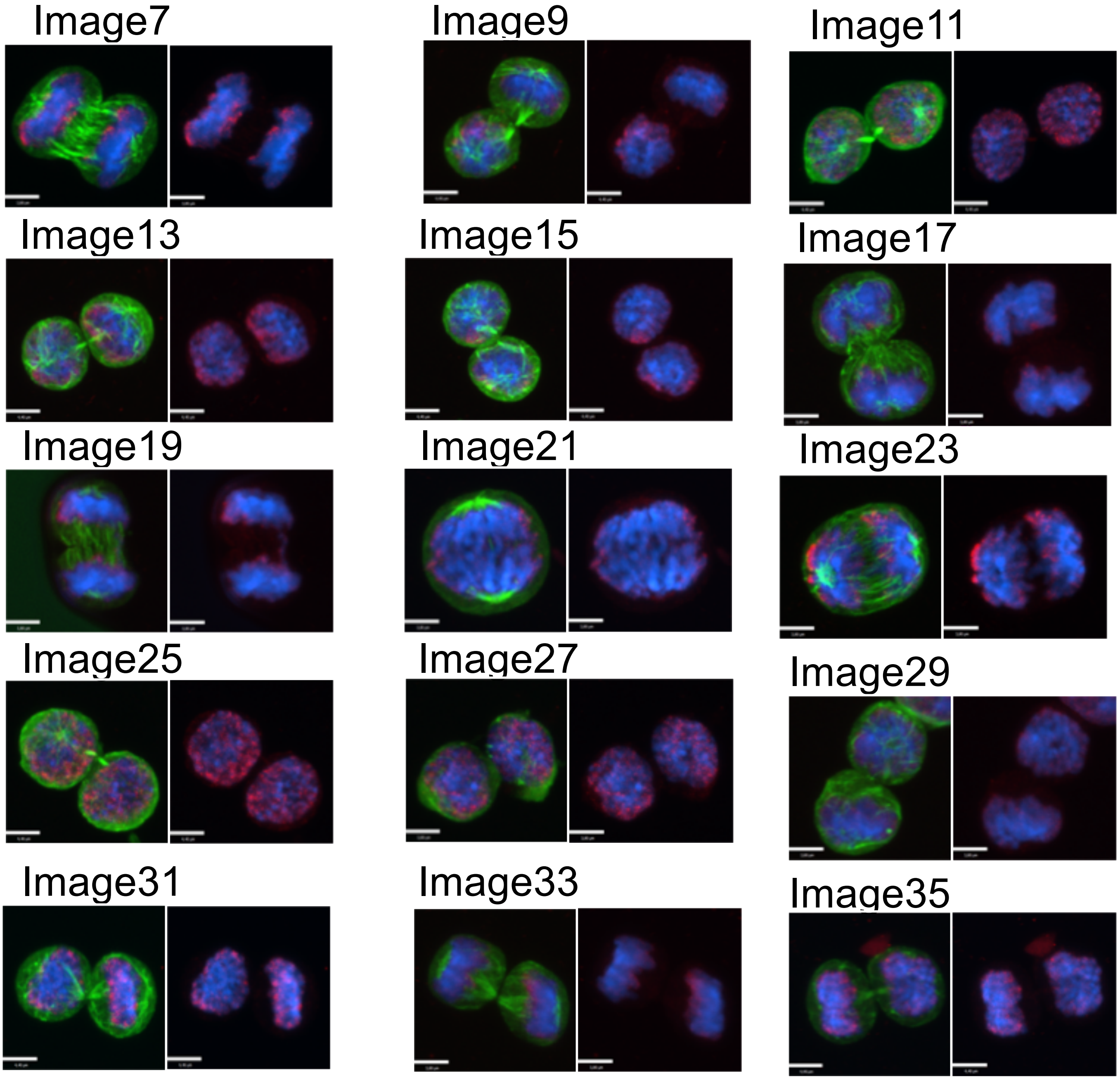

Young ctr; acH4k16 (red), tubulin (green), DAPI (blue); exp#3

Image2

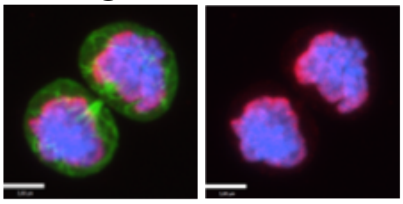

Image4

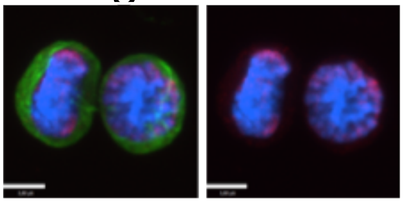

Image6

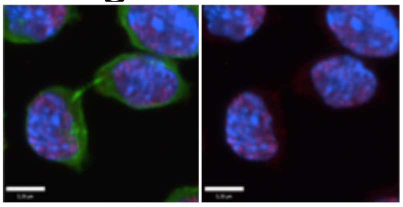

Image8

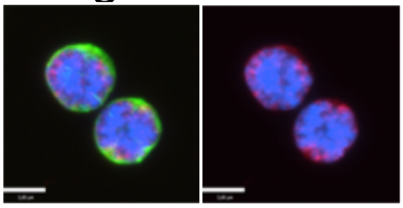

Image11

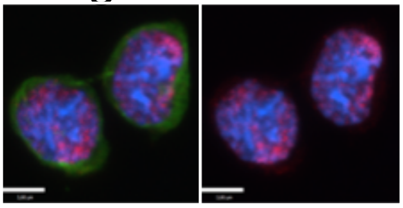

Image13

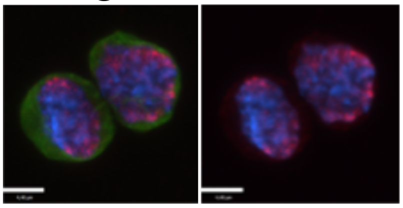

Image17

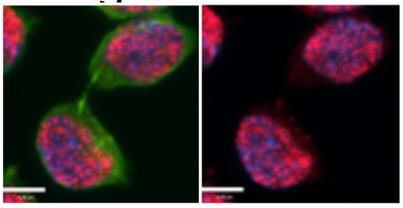

Image21

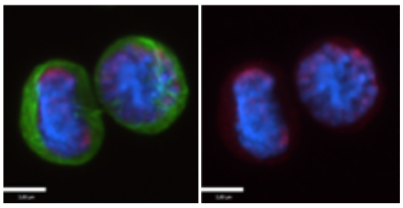

Image37

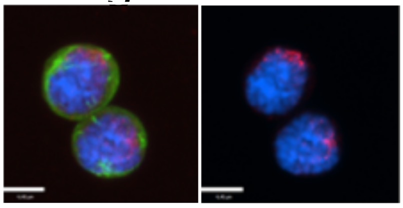

Image41

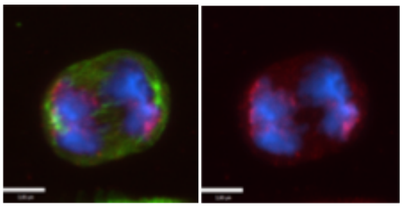

Image32

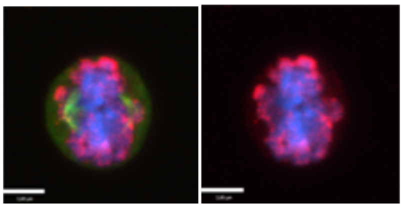

Image45

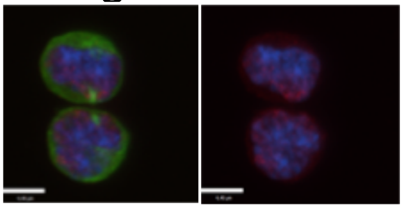

Image47

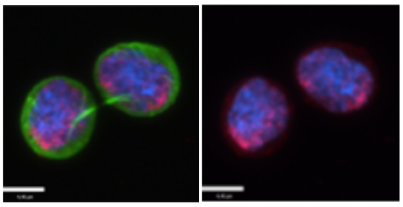

Image43

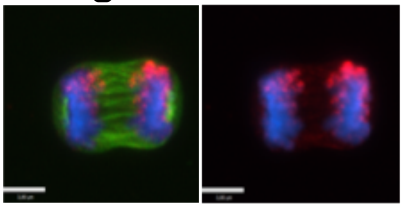

Image49

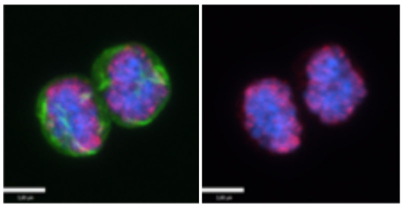

Young+Wnt5a 100ng/ml; acH4k16 (red), tubulin (green), DAPI (blue);
exp#3

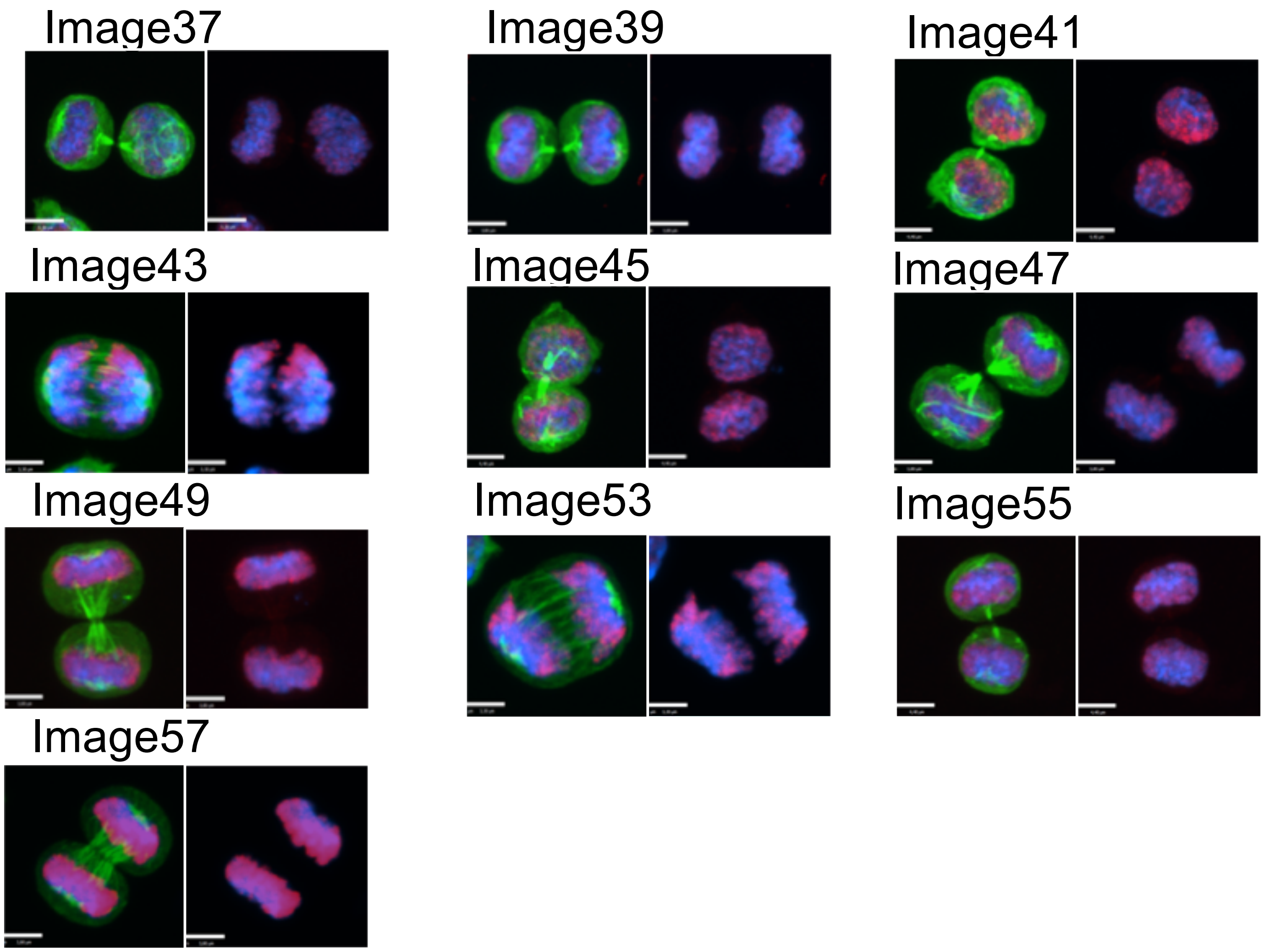

Aged ctr; acH4k16 (red), tubulin (green), DAPI (blue); exp#3

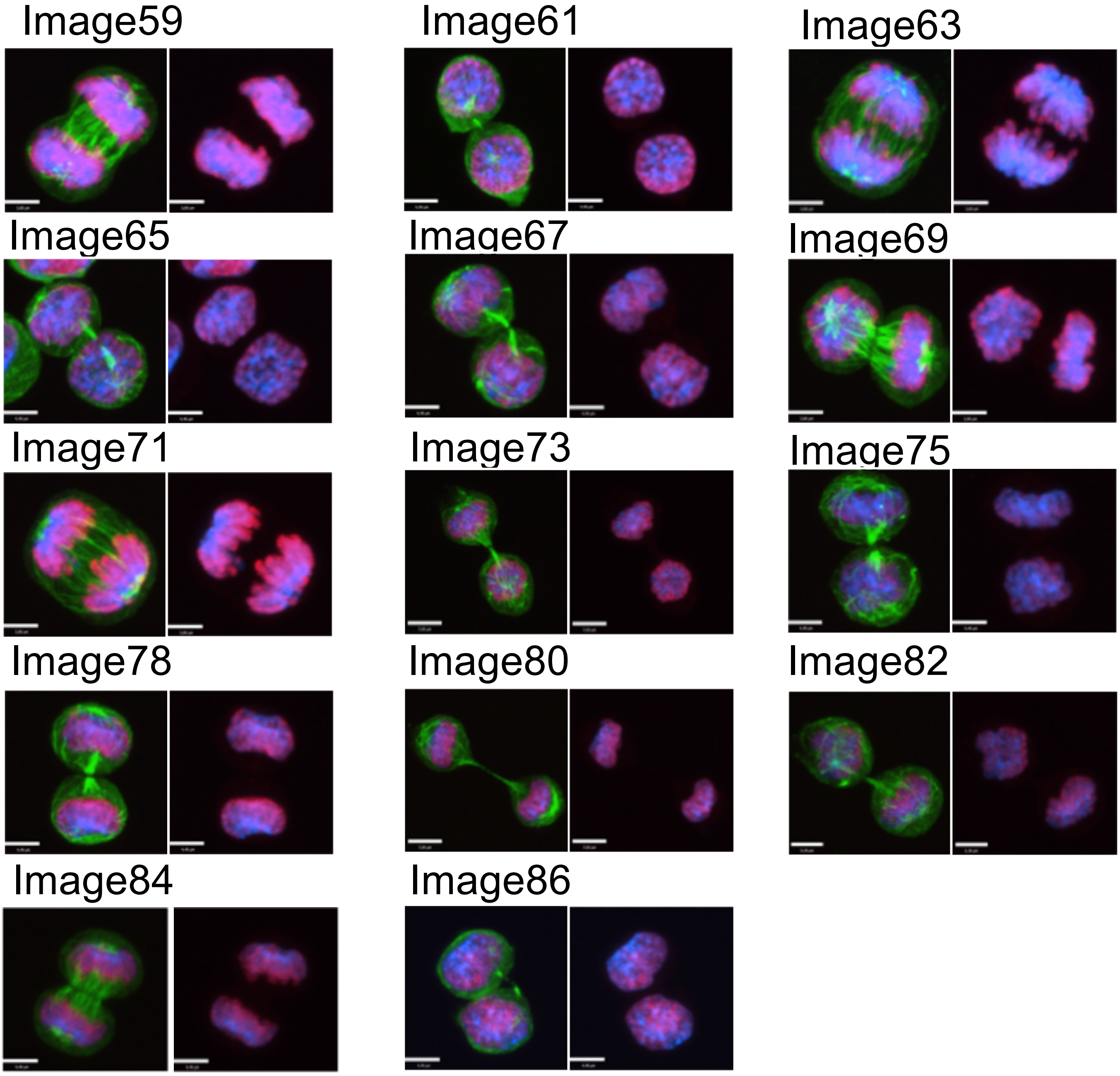

Aged+CASIN5μM; acH4k16 (red), tubulin (green), DAPI (blue);
exp#3

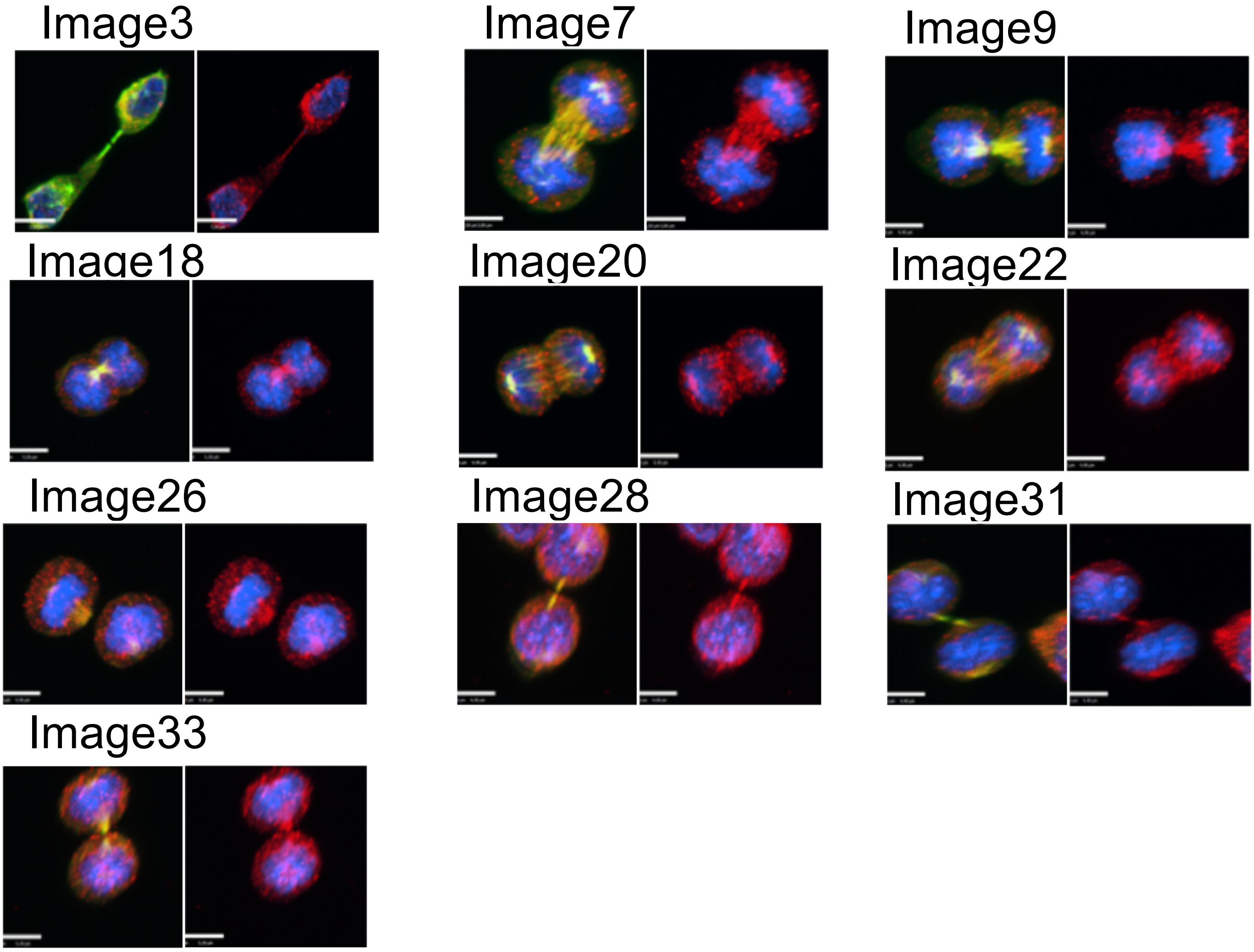

Young ctr; Cdc42 (red), tubulin (green), DAPI (blue);
exp#1

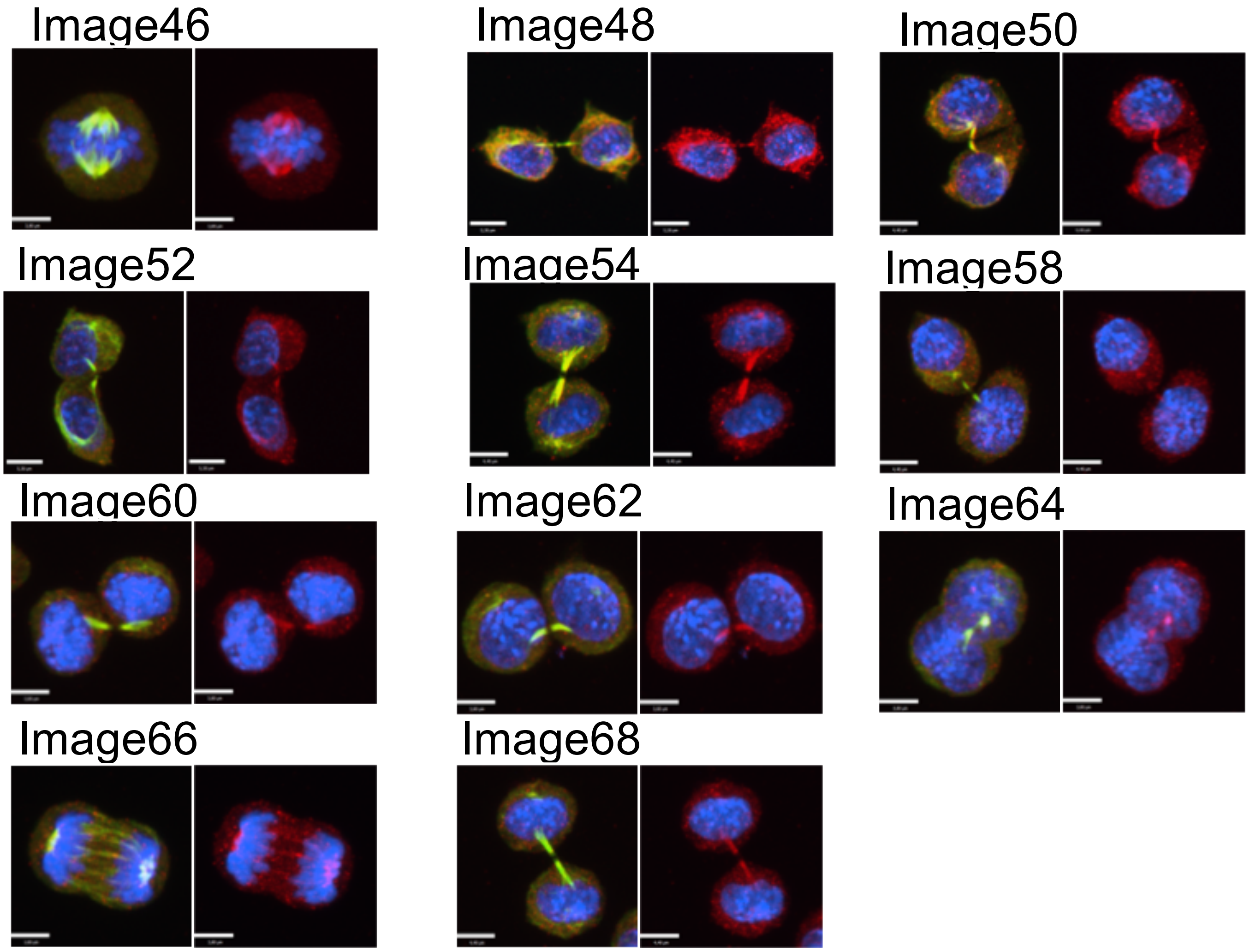

Aged ctr; Cdc42 (red), tubulin (green), DAPI (blue); exp#1

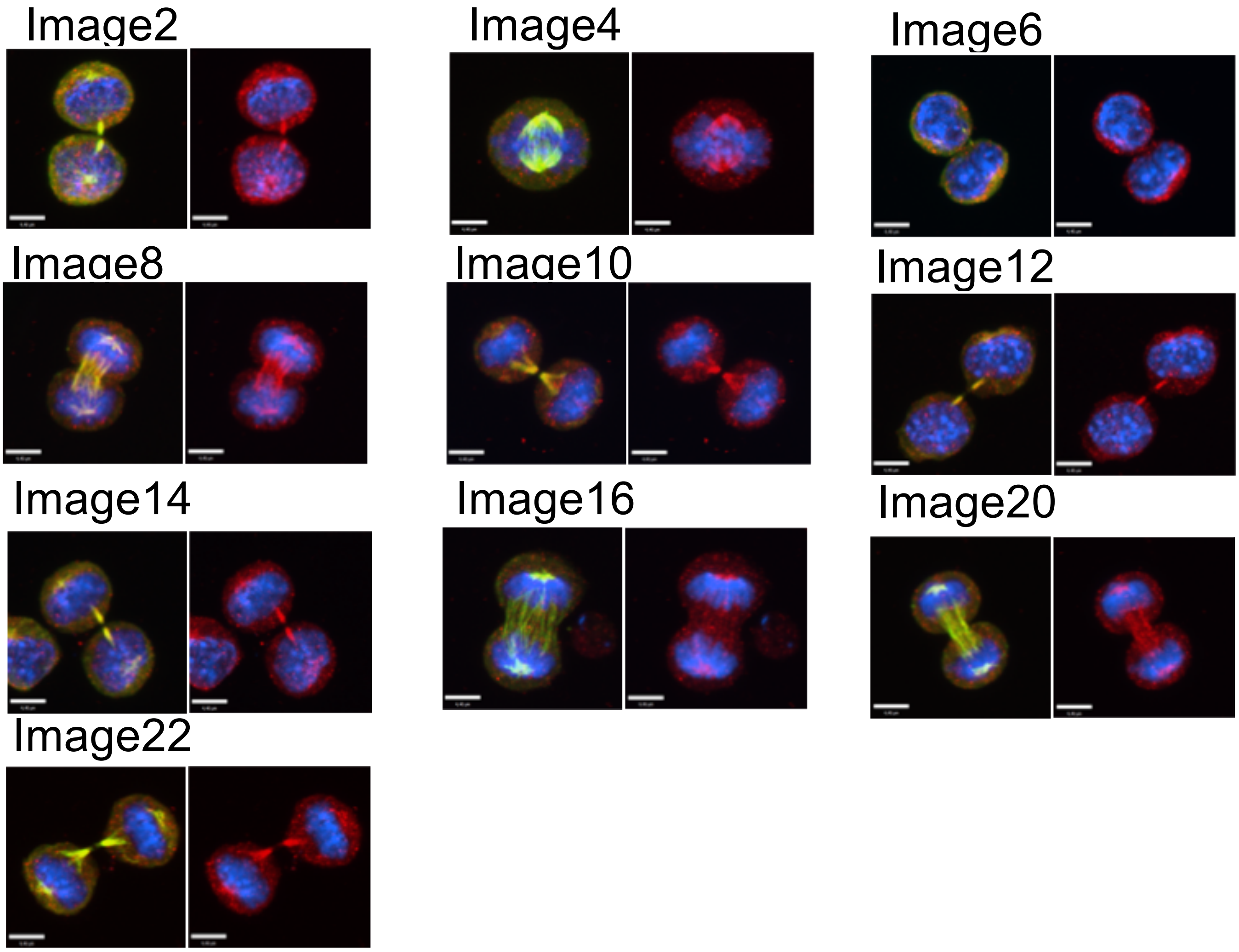

Young+Wnt5a 100ng/ml; Cdc42 (red), tubulin (green), DAPI (blue);
exp#1

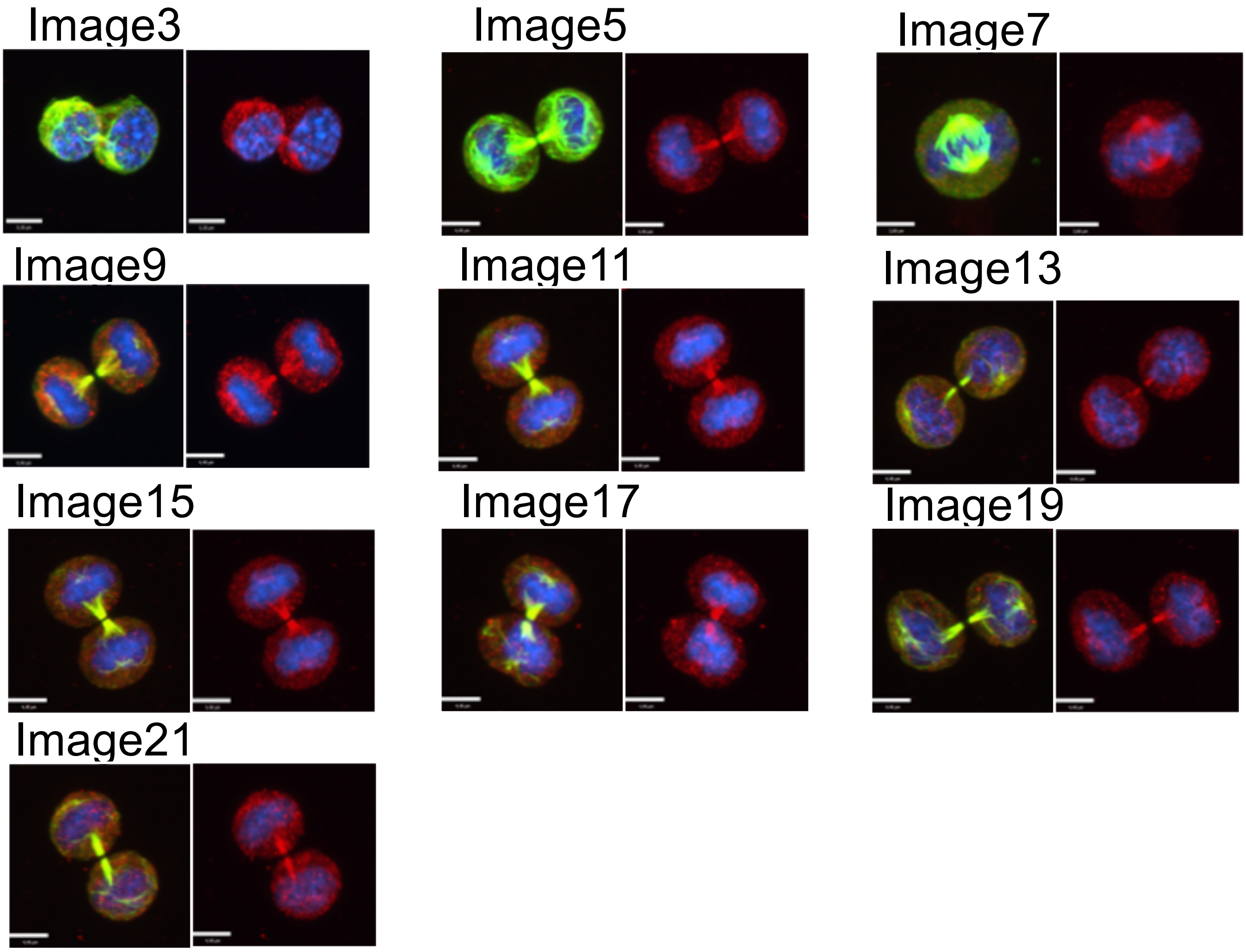

Aged+CASIN 5µM; Cdc42 (red), tubulin (green), DAPI (blue); exp#1

Image11

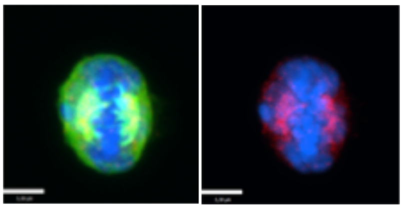

Image13

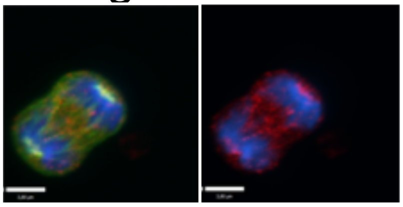

Image15

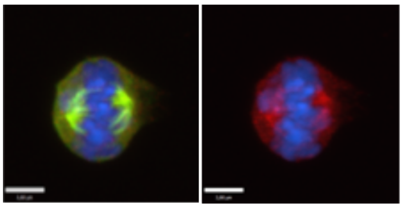

Image33

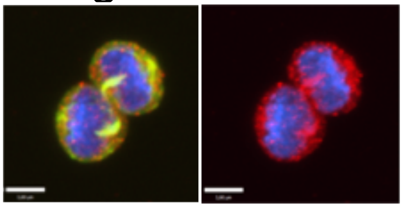

Image61

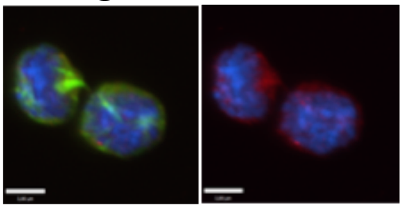

Image37

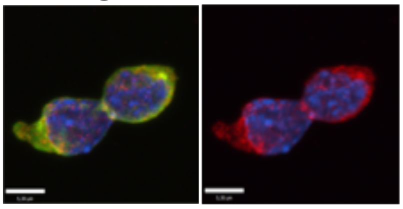

Image39

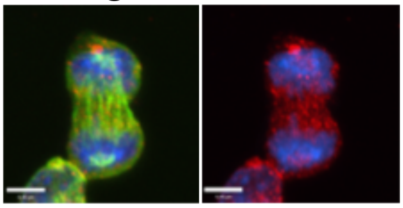

Image41

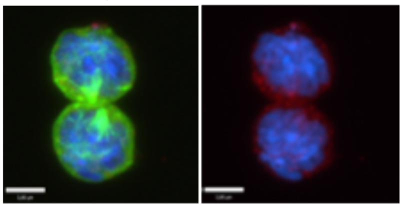

Image43

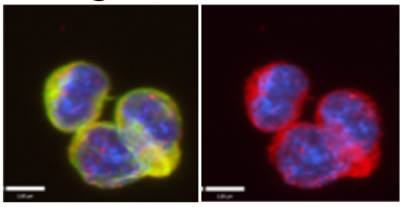

Image63

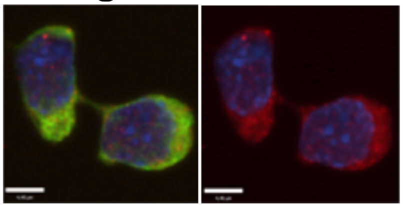

Image47

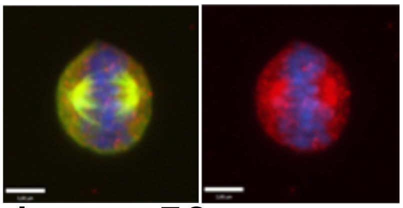

Image49

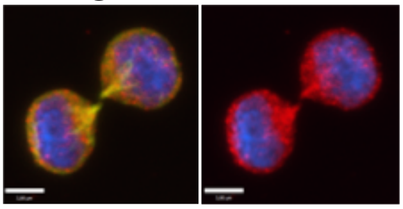

Image51

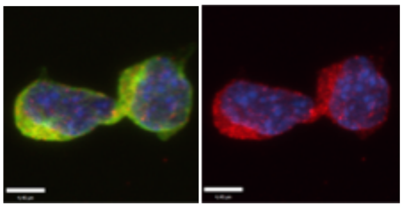

Image53

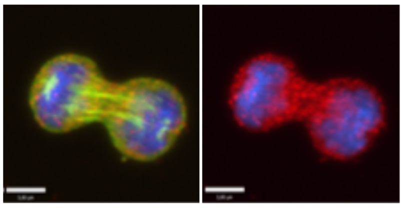

Image55

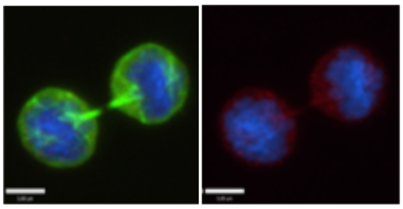

Image57

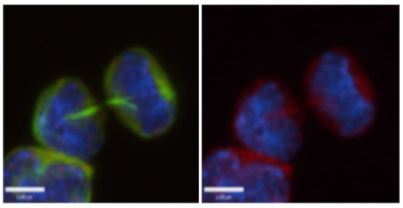

Image59

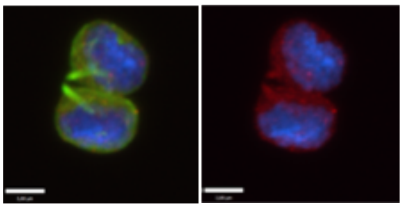

Young ctr; Cdc42 (red), tubulin (green), DAPI (blue); exp#2

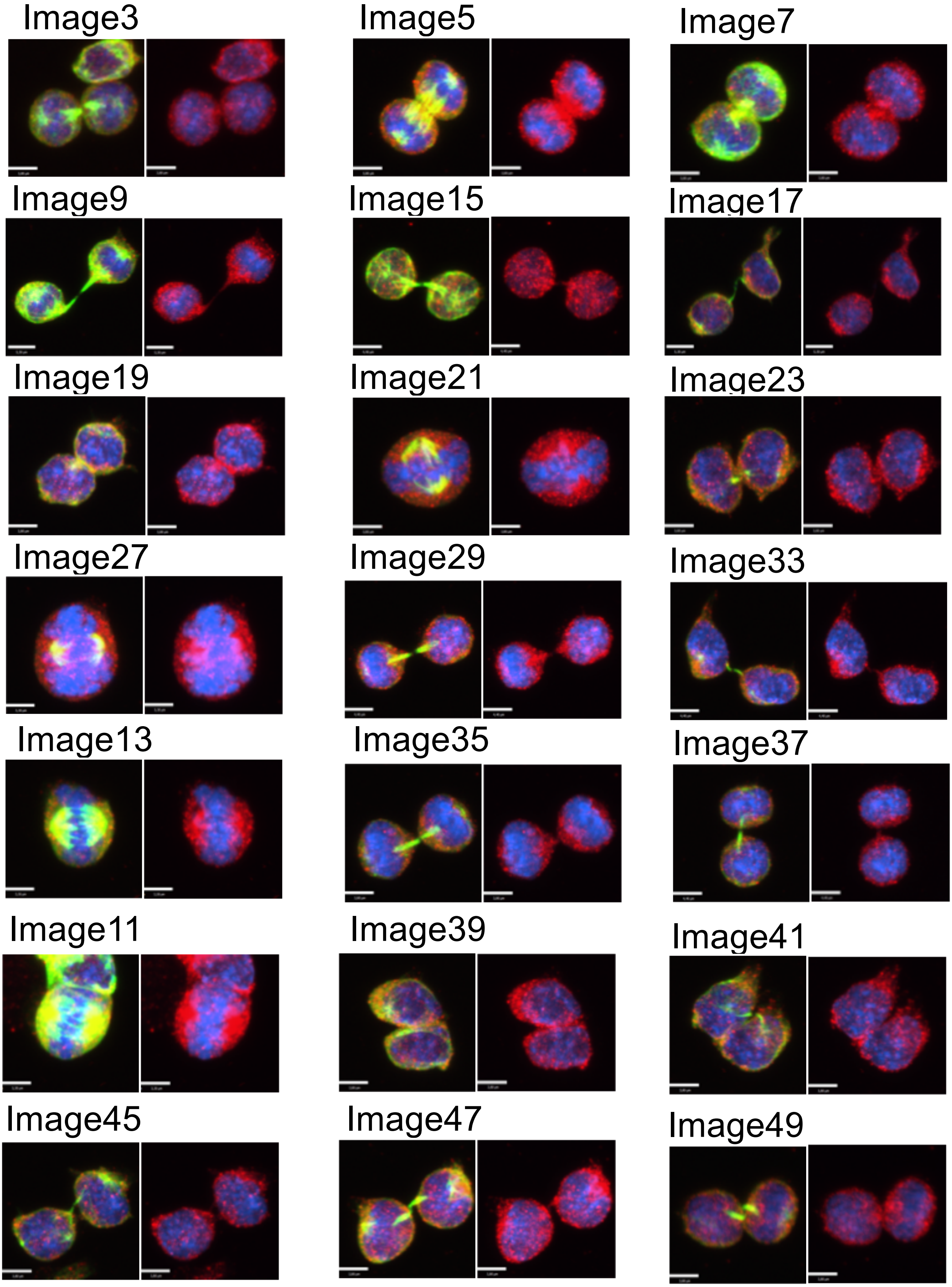

Aged ctr; Cdc42 (red), tubulin (green), DAPI (blue); exp#2

Image31

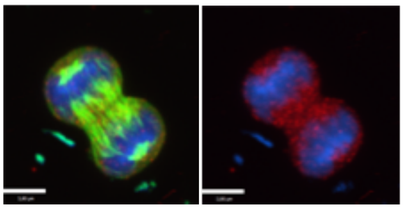

Image33

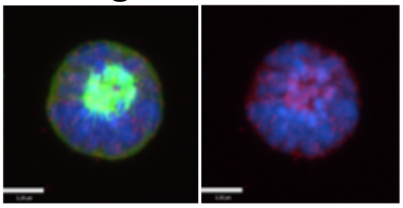

Image37

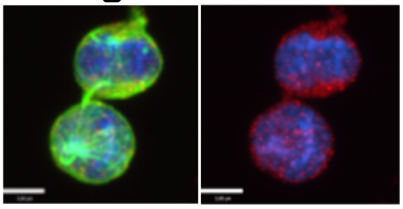

Image39

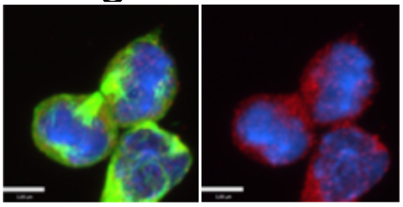

Image41

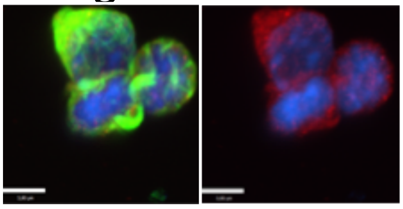

Image45

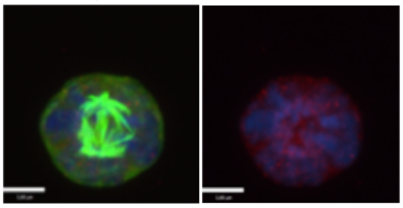

Image47

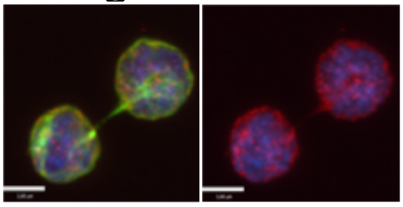

Image49

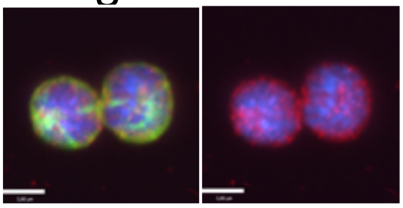

Image53

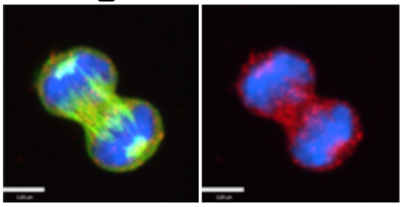

Image57

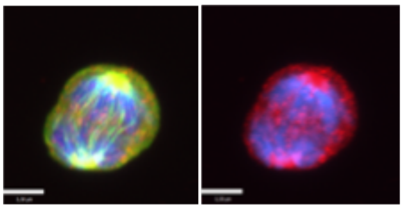

Young+Wnt5a 100ng/ml; Cdc42 (red), tubulin (green), DAPI (blue);
exp#2

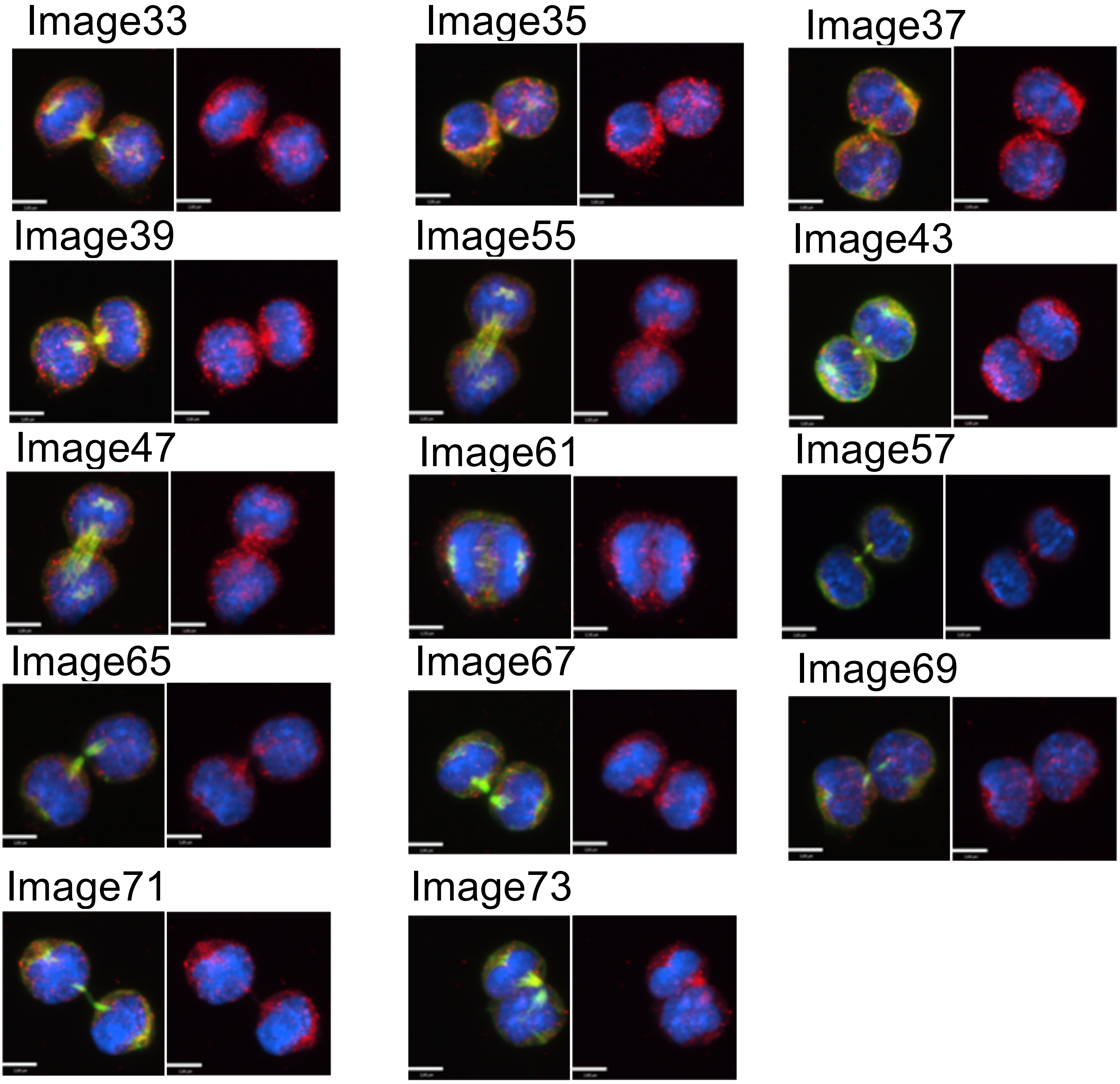

Aged+CASIN 5μM; Cdc42 (red), tubulin (green), DAPI (blue); exp#2
