# Supplementary material for: Aging alters the epigenetic asymmetry of HSC division
Source: PLoS Biol. 2018 Sep 20;16(9):e2003389. doi: 10.1371/journal.pbio.2003389 (PMC6168157; doi:10.1371/journal.pbio.2003389)

B1_CONTROL_O_1_O1_5

1_CONTROL_D_O_1_O11_CONTROL_D_O_1_O1_

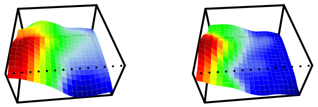

B1_CONTROL_Y_1_Y1_6

1_CONTROL_D_Y_1_Y11_CONTROL_D_Y_1_Y1_

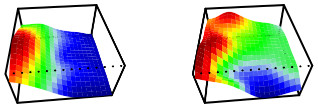

B1_CONTROL_Y_1_Y1_2

1_CONTROL_D_Y_1_Y11_CONTROL_D_Y_1_Y1_

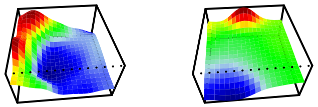

B1_CONTROL_Y_1_Y1_5

1_CONTROL_D_Y_1_Y11_CONTROL_D_Y_1_Y1_

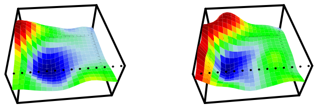

B1_CONTROL_Y_1_Y1_7

1_CONTROL_D_Y_1_Y131_CONTROL_D_Y_1_Y1

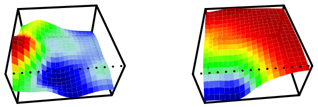

B1_CONTROL_O_1_O1_1

31_CONTROL_D_O_1_O31_CONTROL_D_O_1_O1

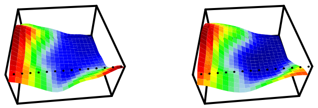

B1_CONTROL_O_1_O1_2

31_CONTROL_D_O_1_O31_CONTROL_D_O_1_O1

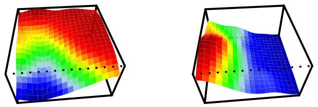

B1_CONTROL_O_1_O1_6

1_CONTROL_D_O_1_O11_CONTROL_D_O_1_O1

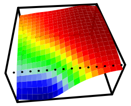
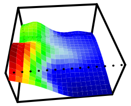

B1_CONTROL_O_1_O1_4

1_CONTROL_D_O_1_O11_CONTROL_D_O_1_O1

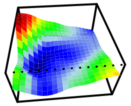
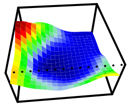

B2_CONTROL_Y_1_Y2_3

2_CONTROL_D_Y_1_Y22_CONTROL_D_Y_1_Y2

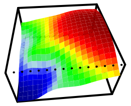
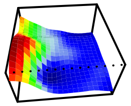

B2_CONTROL_O_1_O2_2

2_CONTROL_D_O_1_O22_CONTROL_D_O_1_O2

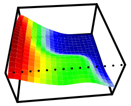
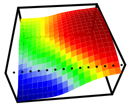

B2_CONTROL_Y_1_Y2_4

2_CONTROL_D_Y_1_Y22_CONTROL_D_Y_1_Y2

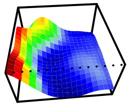
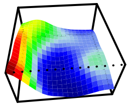

B2_CONTROL_O_1_O2_3

2_CONTROL_D_O_1_O22_CONTROL_D_O_1_O2

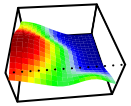
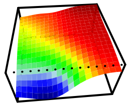

B2_CONTROL_O_1_O2_4

2_CONTROL_D_O_1_O22_CONTROL_D_O_1_O2

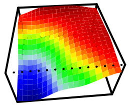
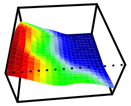

B2_CONTROL_O_1_O2_5

2_CONTROL_D_O_1_O22_CONTROL_D_O_1_O2

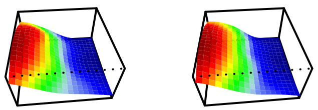

B2_CONTROL_Y_1_Y2_1

2_CONTROL_D_Y_1_Y22_CONTROL_D_Y_1_Y2

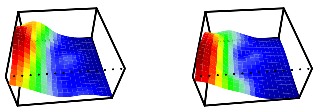

B2_CONTROL_O_1_O2_6

2_CONTROL_D_O_1_O22_CONTROL_D_O_1_O2

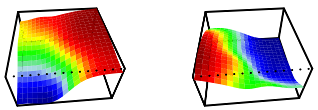

B2_CONTROL_Y_1_Y2_2

2_CONTROL_D_Y_1_Y22_CONTROL_D_Y_1_Y2

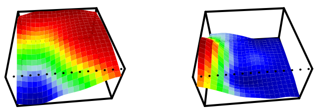

B2_CONTROL_O_1_O2_1

2_CONTROL_D_O_1_O22_CONTROL_D_O_1_O2

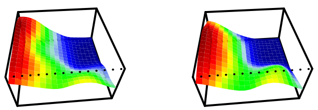

B2_CONTROL_O_1_O2_7

2_CONTROL_D_O_1_O22_CONTROL_D_O_1_O2

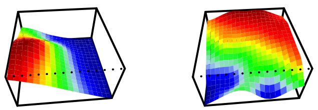

B3_CONTROL_Y_1_Y3_4

3_CONTROL_D_Y_1_Y33_CONTROL_D_Y_1_Y3

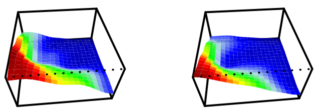

B3_CONTROL_O_1_O3_1

3_CONTROL_D_O_1_O33_CONTROL_D_O_1_O3

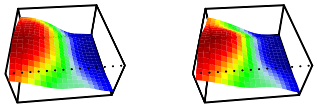

B3_CONTROL_Y_1_Y3_5

3_CONTROL_D_Y_1_Y33_CONTROL_D_Y_1_Y3

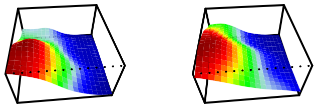

B3_CONTROL_O_1_O3_2

3_CONTROL_D_O_1_O33_CONTROL_D_O_1_O3

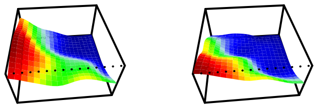

B3_CONTROL_Y_1_Y3_7

3_CONTROL_D_Y_1_Y33_CONTROL_D_Y_1_Y3

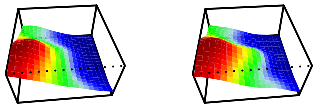

B3_CONTROL_O_1_O3_3

3_CONTROL_D_O_1_O33_CONTROL_D_O_1_O3

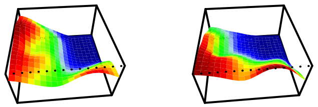

B3_CONTROL_O_1_O3_5

3_CONTROL_D_O_1_O33_CONTROL_D_O_1_O3

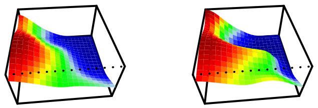

B3_CONTROL_Y_1_Y3_1

3_CONTROL_D_Y_1_Y33_CONTROL_D_Y_1_Y3

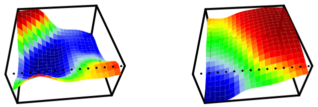

B3_CONTROL_O_1_O3_6

3_CONTROL_D_O_1_O33_CONTROL_D_O_1_O3

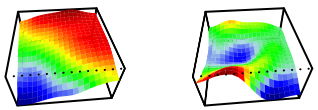

B3_CONTROL_Y_1_Y3_2

3_CONTROL_D_Y_1_Y33_CONTROL_D_Y_1_Y3

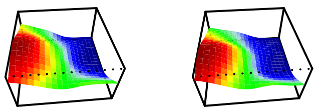

B3_CONTROL_O_1_O3_7

3_CONTROL_D_O_1_O33_CONTROL_D_O_1_O3

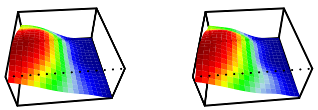

TY2O3P1_CASIN_O_1_O5_1

'2O3P1_CASIN_D_O_1_O2O3P1_CASIN_D_O_1_(

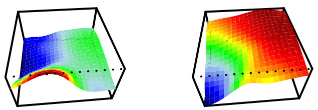

TY1_CONTROL_Y_1_Y4_4

Y1_CONTROL_D_Y_1_Y4Y1_CONTROL_D_Y_1_Y

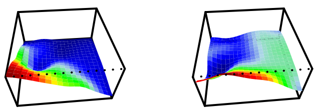

TY2O3P1_CASIN_O_1_O5_7

'2O3P1_CASIN_D_O_1_O2O3P1_CASIN_D_O_1_(

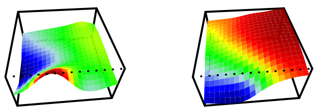

TO2_CONTROL_O_1_O4_5

O2_CONTROL_D_O_1_OO2_CONTROL_D_O_1_O

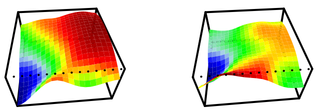

TY3_CONTROL_Y_1_Y6_2

Y3_CONTROL_D_Y_1_Y(Y3_CONTROL_D_Y_1_Y(

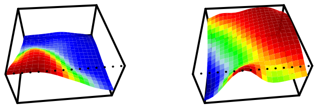

TO2_CASIN_O_1_O4_4

TO2_CASIN_D_O_1_O4_TO2_CASIN_D_O_1_O4_

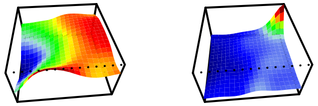

TY3_WNT5A_Y_1_Y6_2

TY3_WNT5A_D_Y_1_Y6_TY3_WNT5A_D_Y_1_Y6_

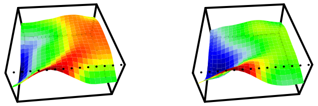

TY3_WNT5A_Y_1_Y6_8

TY3_WNT5A_D_Y_1_Y6_TY3_WNT5A_D_Y_1_Y6_

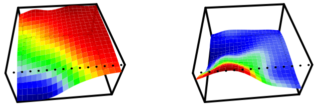

TY2O3P1_CONTROL_Y_1_Y5_3

O3P1_CONTROL_D_Y_1O3P1_CONTROL_D_Y_1_

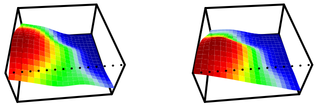

TO4_CONTROL_O_1_O6_6

O4_CONTROL_D_O_1_OO4_CONTROL_D_O_1_O(

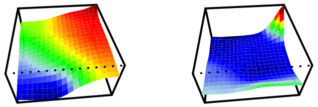

TY2O3P2_WNT5A_Y_1_Y5_5

2O3P2_WNT5A_D_Y_1_Y2O3P2_WNT5A_D_Y_1_Y

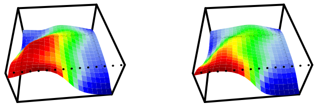

TO4_CASIN_O_1_O6_6

TO4_CASIN_D_O_1_O6_TO4_CASIN_D_O_1_O6_

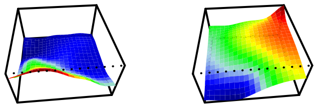

TY2O3P2_CONTROL_O_1_O5_3

O3P2_CONTROL_D_O_1 O3P2_CONTROL_D_O_1

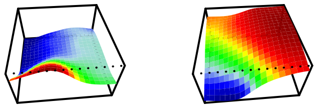

TY2O3P1_CASIN_O_1_O5_2

Y2O3P1_CASIN_D_O_1_C2O3P1_CASIN_D_O_1_C

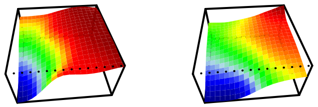

TY1_CONTROL_Y_1_Y4_5

Y1_CONTROL_D_Y_1_Y4Y1_CONTROL_D_Y_1_Y4

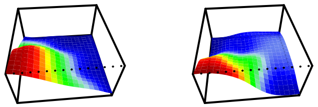

TY2O3P1_CASIN_O_1_O5_8

Y2O3P1_CASIN_D_O_1_C2O3P1_CASIN_D_O_1_C

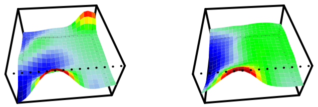

TY2O3P2_CONTROL_O_1_O5_4

O3P2_CONTROL_D_O_1 O3P2_CONTROL_D_O_1

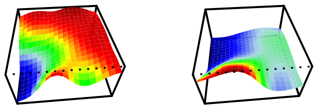

TO2_CONTROL_O_1_O4_6

O2_CONTROL_D_O_1_OO2_CONTROL_D_O_1_O

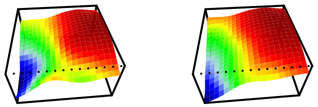

TY3_CONTROL_Y_1_Y6_3

Y3_CONTROL_D_Y_1_Y(Y3_CONTROL_D_Y_1_Y

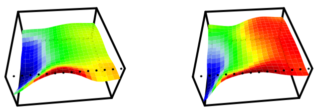

TO2_CASIN_O_1_O4_5

TO2_CASIN_D_O_1_O4_ TO2_CASIN_D_O_1_O4_

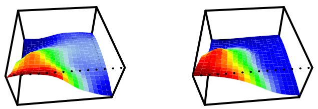

TY3_WNT5A_Y_1_Y6_3

TY3_WNT5A_D_Y_1_Y6_ TY3_WNT5A_D_Y_1_Y6_

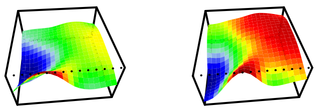

TY3_WNT5A_Y_1_Y6_11

TY3_WNT5A_D_Y_1_Y6_ TY3_WNT5A_D_Y_1_Y6_

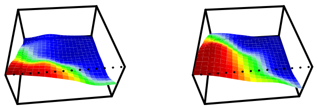

TO4_CONTROL_O_1_O6_1

O4_CONTROL_D_O_1_O O4_CONTROL_D_O_1_O

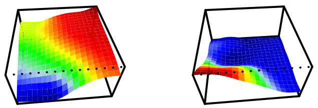

TY2O3P2_CONTROL_Y_1_Y5_4

O3P2_CONTROL_D_Y_1 O3P2_CONTROL_D_Y_1

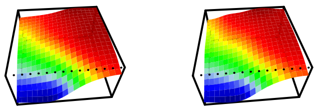

TO4_CONTROL_O_1_O6_7

O4_CONTROL_D_O_1_O O4_CONTROL_D_O_1_O

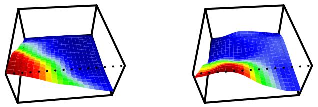

TY2O3P2_WNT5A_Y_1_Y5_6

Y2O3P2_WNT5A_D_Y_1_ Y2O3P2_WNT5A_D_Y_1_

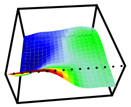
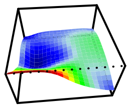

TO4_CASIN_O_1_O6_7

TO4_CASIN_D_O_1_O6_ TO4_CASIN_D_O_1_O6_

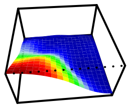
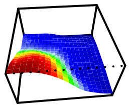

TY2O3P1_CASIN_O_1_O5_3

Y2O3P1_CASIN_D_O_1_(Y2O3P1_CASIN_D_O_1_(

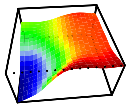
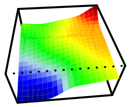

TY1_WNT5A_Y_1_Y4_1

TY1_WNT5A_D_Y_1_Y4_ TY1_WNT5A_D_Y_1_Y4_

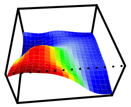
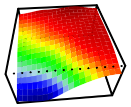

TY2O3P2_CONTROL_O_1_O5_5

O3P2_CONTROL_D_O_1 O3P2_CONTROL_D_O_1

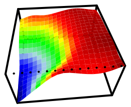
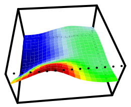

TO2_CONTROL_O_1_O4_1

O2_CONTROL_D_O_1_O O2_CONTROL_D_O_1_O

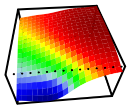
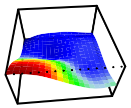

TO2_CONTROL_O_1_O4_7

O2_CONTROL_D_O_1_O O2_CONTROL_D_O_1_O

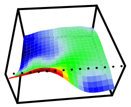
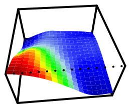

TY3_CONTROL_Y_1_Y6_4

Y3_CONTROL_D_Y_1_YY3_CONTROL_D_Y_1_Y

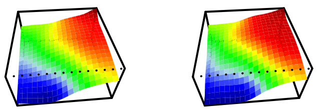

TO2_CASIN_O_1_O4_6

TO2_CASIN_D_O_1_O4_TO2_CASIN_D_O_1_O4_

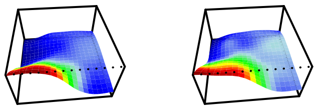

TY3_WNT5A_Y_1_Y6_4

TY3_WNT5A_D_Y_1_Y6_TY3_WNT5A_D_Y_1_Y6_

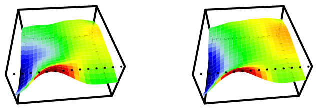

TY3_WNT5A_Y_1_Y6_12

TY3_WNT5A_D_Y_1_Y6_TY3_WNT5A_D_Y_1_Y6_

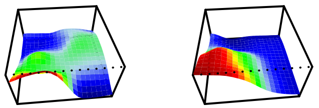

TO4_CONTROL_O_1_O6_2

O4_CONTROL_D_O_1_OO4_CONTROL_D_O_1_O

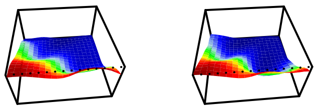

TY2O3P2_WNT5A_Y_1_Y5_1

Y2O3P2_WNT5A_D_Y_1_Y2O3P2_WNT5A_D_Y_1_

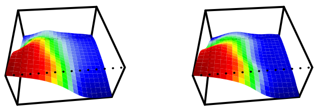

TO4_CASIN_O_1_O6_1

TO4_CASIN_D_O_1_O6_TO4_CASIN_D_O_1_O6_

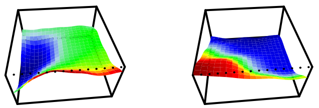

TY3_WNT5A_Y_1_Y6_13

TY3_WNT5A_D_Y_1_Y6 TY3_WNT5A_D_Y_1_Y6_

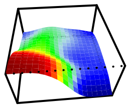

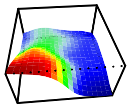

TY2O3P1_CASIN_O_1_O5_4

Y2O3P1_CASIN_D_O_1_ Y2O3P1_CASIN_D_O_1_

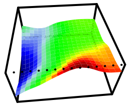

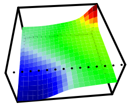

TY1_WNT5A_Y_1_Y4_2

TY1_WNT5A_D_Y_1_Y4 TY1_WNT5A_D_Y_1_Y4

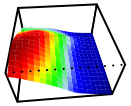

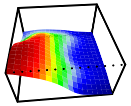

TY2O3P2_CONTROL_O_1_O5_6

O3P2_CONTROL_D_O_1 O3P2_CONTROL_D_O_1

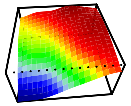

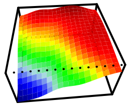

TY1_CONTROL_Y_1_Y4_1

Y1_CONTROL_D_Y_1_Y Y1_CONTROL_D_Y_1_Y

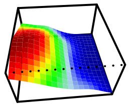

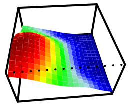

TO4_CONTROL_O_1_O6_3

O4_CONTROL_D_O_1_C O4_CONTROL_D_O_1_C

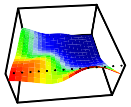

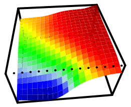

TO2_CONTROL_O_1_O4_2

O2_CONTROL_D_O_1_C O2_CONTROL_D_O_1_C

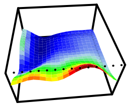

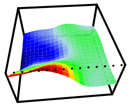

TO2_CASIN_O_1_O4_1

TO2_CASIN_D_O_1_O4_ TO2_CASIN_D_O_1_O4_

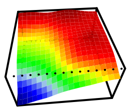 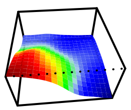

TY3_CONTROL_Y_1_Y6_5

Y3_CONTROL_D_Y_1_Y Y3_CONTROL_D_Y_1_Y

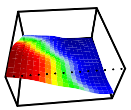 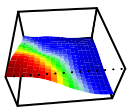

TO2_CASIN_O_1_O4_7

TO2_CASIN_D_O_1_O4_ TO2_CASIN_D_O_1_O4_

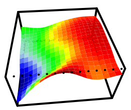 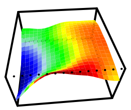

TY3_WNT5A_Y_1_Y6_5

TY3_WNT5A_D_Y_1_Y6_ TY3_WNT5A_D_Y_1_Y6_

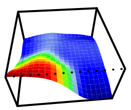 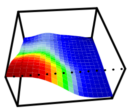

TY2O3P2_WNT5A_Y_1_Y5_2

Y2O3P2_WNT5A_D_Y_1_ Y2O3P2_WNT5A_D_Y_1_

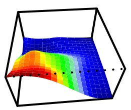 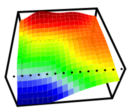

TO4_CASIN_O_1_O6_3

TO4_CASIN_D_O_1_O6_ TO4_CASIN_D_O_1_O6_

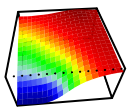 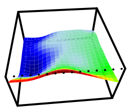

TY2O3P1_CASIN_O_1_O5_5

Y2O3P1_CASIN_D_O_1_ Y2O3P1_CASIN_D_O_1_

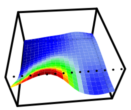 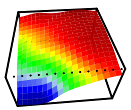

TY1_WNT5A_Y_1_Y4_3

TY1_WNT5A_D_Y_1_Y4 TY1_WNT5A_D_Y_1_Y4

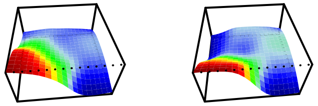

TY2O3P2_CONTROL_O_1_O5_7

O3P2_CONTROL_D_O_1O3P2_CONTROL_D_O_1

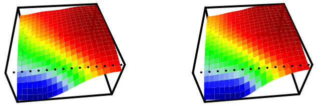

TY1_CONTROL_Y_1_Y4_2

Y1_CONTROL_D_Y_1_YY1_CONTROL_D_Y_1_Y

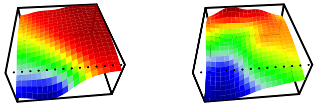

TO4_CONTROL_O_1_O6_4

O4_CONTROL_D_O_1_CO4_CONTROL_D_O_1_C

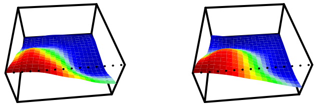

TO2_CONTROL_O_1_O4_3

O2_CONTROL_D_O_1_CO2_CONTROL_D_O_1_C

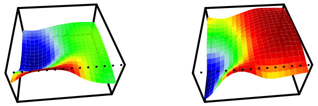

TO2_CASIN_O_1_O4_2

TO2_CASIN_D_O_1_O4 TO2_CASIN_D_O_1_O4

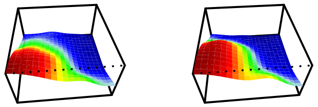

TY3_CONTROL_Y_1_Y6_6

Y3_CONTROL_D_Y_1_YY3_CONTROL_D_Y_1_Y

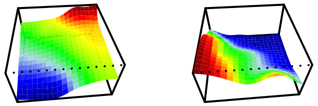

TO2_CASIN_O_1_O4_8

TO2_CASIN_D_O_1_O4_ TO2_CASIN_D_O_1_O4_

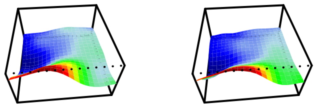

TY3_WNT5A_Y_1_Y6_6

TY3_WNT5A_D_Y_1_Y6_ TY3_WNT5A_D_Y_1_Y6_

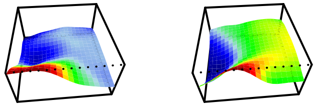

TY2O3P1_CONTROL_Y_1_Y5_1

2O3P1_CONTROL_D_Y_1 1O3P1_CONTROL_D_Y_1

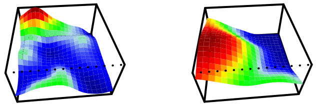

TY2O3P2_WNT5A_Y_1_Y5_3

Y2O3P2_WNT5A_D_Y_1_ Y2O3P2_WNT5A_D_Y_1_

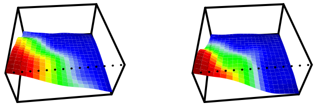

TO4_CASIN_O_1_O6_4

TO4_CASIN_D_O_1_O6_ TO4_CASIN_D_O_1_O6_

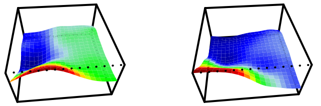

TY2O3P1_CASIN_O_1_O5_6

Y2O3P1_CASIN_D_O_1_(Y2O3P1_CASIN_D_O_1_(

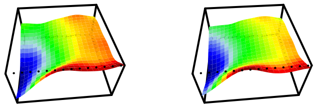

TY2O3P2_CONTROL_O_1_O5_2

O3P2_CONTROL_D_O_1 O3P2_CONTROL_D_O_1

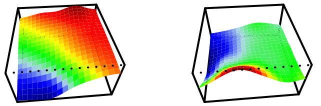

TY2O3P2_CONTROL_O_1_O5_8

O3P2_CONTROL_D_O_1 O3P2_CONTROL_D_O_1

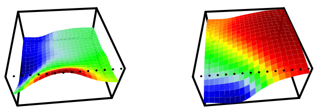

TY1_CONTROL_Y_1_Y4_3

Y1_CONTROL_D_Y_1_Y Y1_CONTROL_D_Y_1_Y

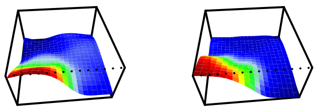

TO4_CONTROL_O_1_O6_5

O4_CONTROL_D_O_1_C O4_CONTROL_D_O_1_C

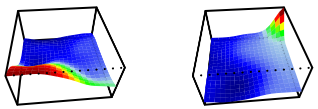

TO2_CONTROL_O_1_O4_4

O2_CONTROL_D_O_1_C O2_CONTROL_D_O_1_C

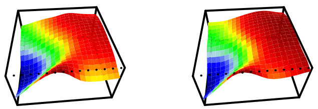

TO2_CASIN_O_1_O4_3

TO2_CASIN_D_O_1_O4 TO2_CASIN_D_O_1_O4

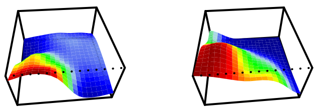

TY3_CONTROL_Y_1_Y6_9

Y3_CONTROL_D_Y_1_Y Y3_CONTROL_D_Y_1_Y

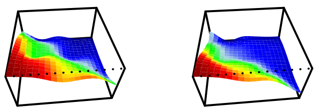

TO2_CASIN_O_1_O4_9

TO2_CASIN_D_O_1_O4 TO2_CASIN_D_O_1_O4

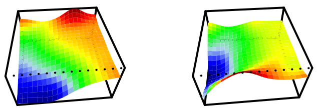

TY3_WNT5A_Y_1_Y6_7

TY3_WNT5A_D_Y_1_Y6 TY3_WNT5A_D_Y_1_Y6

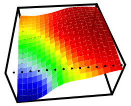

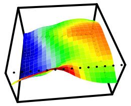

TY2O3P1_CONTROL_Y_1_Y5_2

2O3P1_CONTROL_D_Y_1 O3P1_CONTROL_D_Y_1

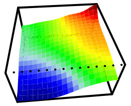

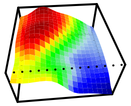

TY2O3P2_WNT5A_Y_1_Y5_4

Y2O3P2_WNT5A_D_Y_1_ 2O3P2_WNT5A_D_Y_1_

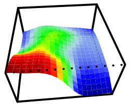

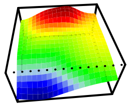

TO4_CASIN_O_1_O6_5

TO4_CASIN_D_O_1_O6 TO4_CASIN_D_O_1_O6

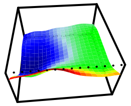

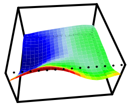

Supplement: S10 Fig — (PDF) [file pbio.2003389.s012.pdf]
