# Supplementary material for: Aging alters the epigenetic asymmetry of HSC division
Source: PLoS Biol. 2018 Sep 20;16(9):e2003389. doi: 10.1371/journal.pbio.2003389 (PMC6168157; doi:10.1371/journal.pbio.2003389)
Supplement: S16 Fig — We simultaneously interrogated the correlation to 13 previously published hematopoietic stem cell and polarity signatures [32–39], and each daughter cell pair was depicted in a radar plot where vertices in the plot correspond to each of the considered gene sets: Mm_HSC_Runx1_Wu [32], Mm_HSC_Tcf7_Wu [32], Mm_LT_HSC_Venezia [36], Mm_Proliferation_Venezia [36], Mm_Quiescence_Venezia [36], Polarity_factors_Ting [37], Novel_HSC_regul_polar_Ting [37], Grover et al, 2016 [38] and Kowalczyk et al, 2015 [39]. Gene signatures that showed a significant enrichment based on a hypergeometric test (adjusted p < 0.05) are shown by red and blue shades representing the 2 cells in a given pair. Signatures not extending beyond the shaded gray center were considered not significant. (PDF) [file pbio.2003389.s018.pdf]

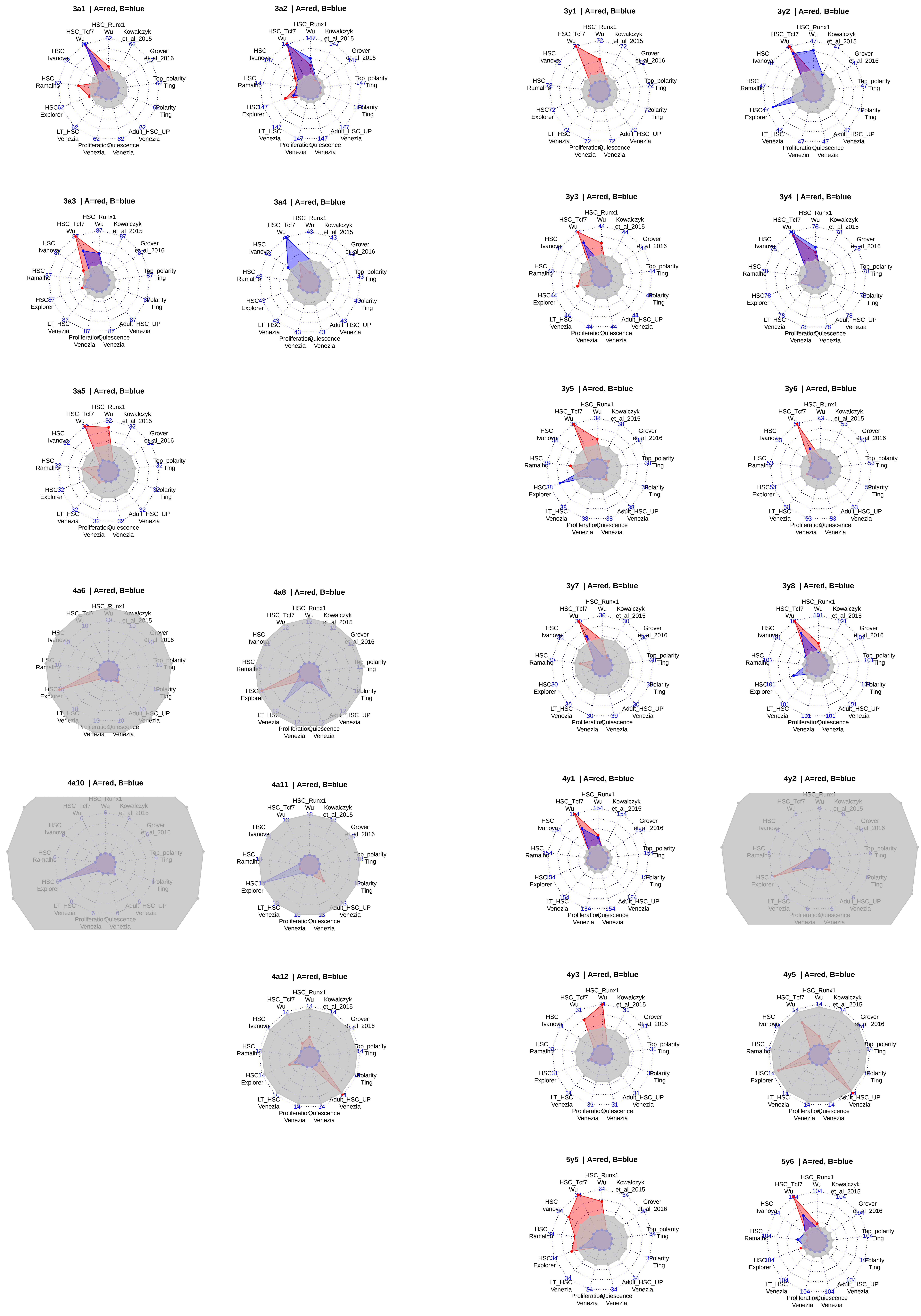

3a1 | A=red, B=blue
3a2 | A=red, B=blue
3y1 | A=red, B=blue
3y2 | A=red, B=blue
3a3 | A=red, B=blue
3a4 | A=red, B=blue
3y3 | A=red, B=blue
3y4 | A=red, B=blue
3a5 | A=red, B=blue
3y5 | A=red, B=blue
3y6 | A=red, B=blue
4a6 | A=red, B=blue
4a8 | A=red, B=blue
3y7 | A=red, B=blue
3y8 | A=red, B=blue
4a10 | A=red, B=blue
4a11 | A=red, B=blue
4y1 | A=red, B=blue
4y2 | A=red, B=blue
4a12 | A=red, B=blue
4y3 | A=red, B=blue
4y5 | A=red, B=blue
5y5 | A=red, B=blue
5y6 | A=red, B=blue
HSC_Runx1
HSC_Tcf7 Wu
Kowalczyk et_al_2015
Grover et_al_2016
Top_polarity Ting
Polarity Ting
Adult_HSC_UP Venezia
Quiescence Venezia
Proliferation Venezia
LT_HSC Venezia
HSC Explorer
HSC Ramalho
HSC Ivanova
