# Supplementary material for: Aging alters the epigenetic asymmetry of HSC division
Source: PLoS Biol. 2018 Sep 20;16(9):e2003389. doi: 10.1371/journal.pbio.2003389 (PMC6168157; doi:10.1371/journal.pbio.2003389)

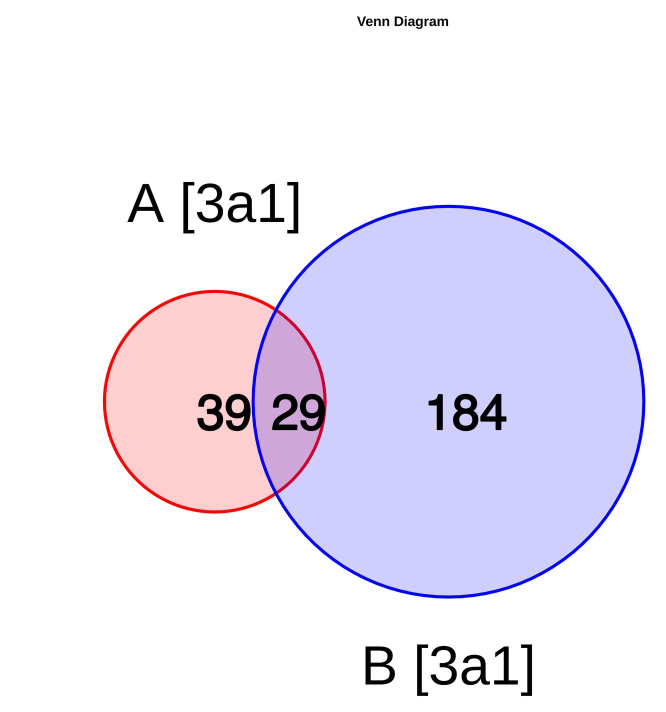

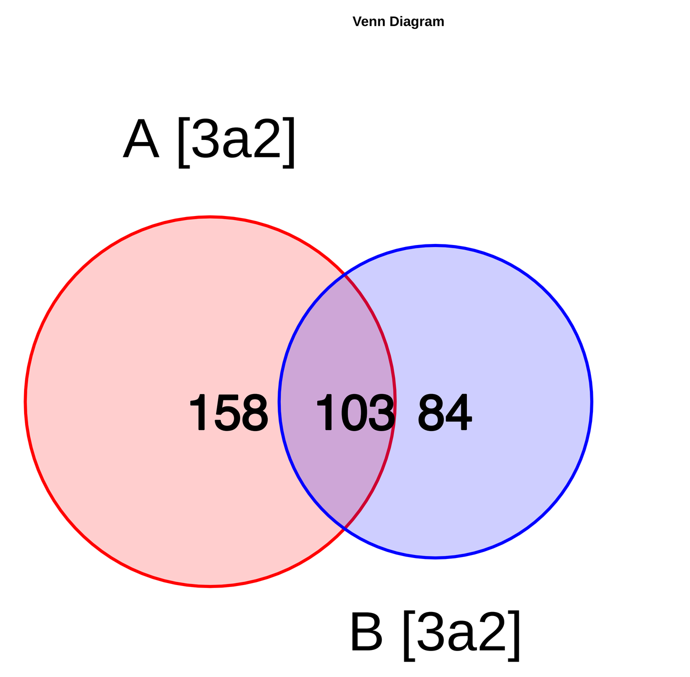

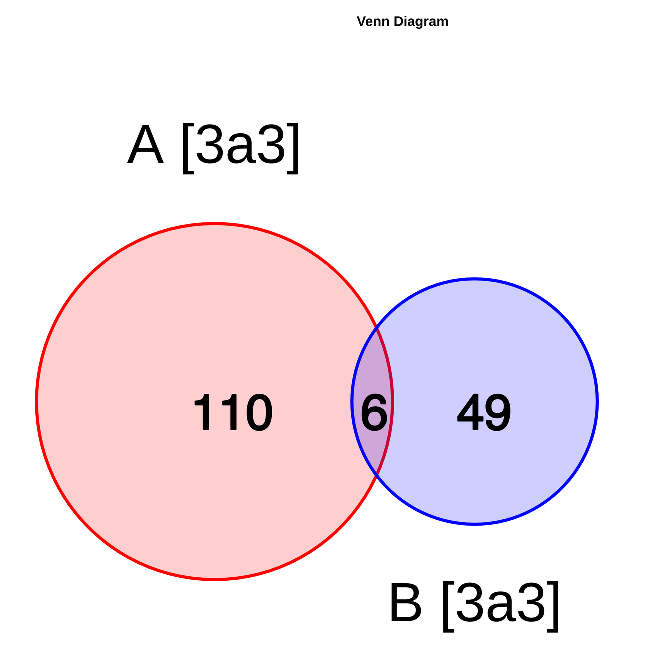

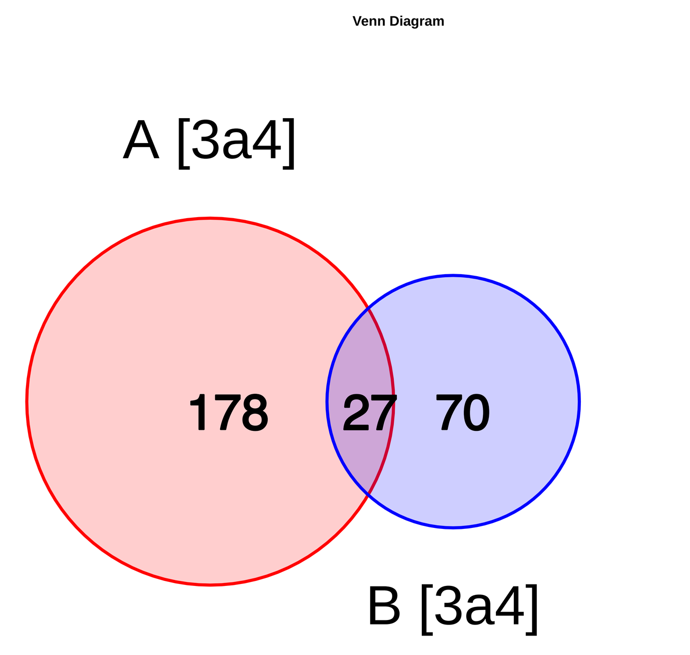

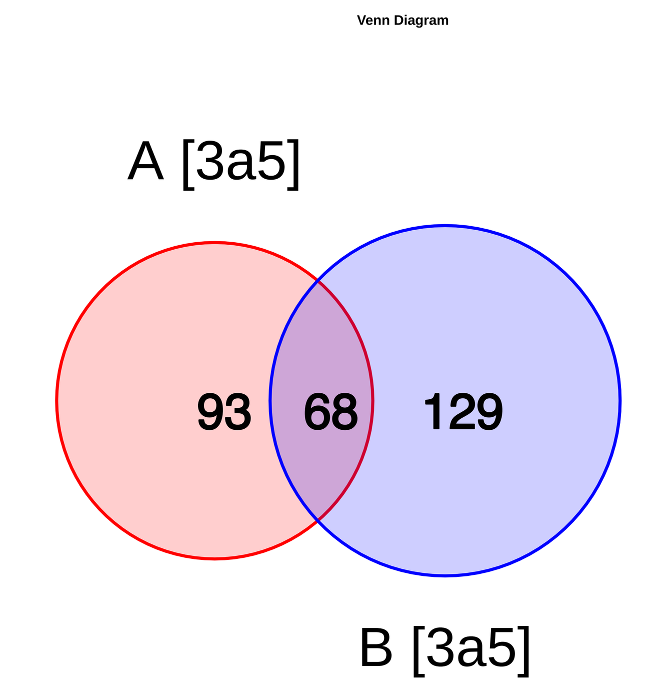

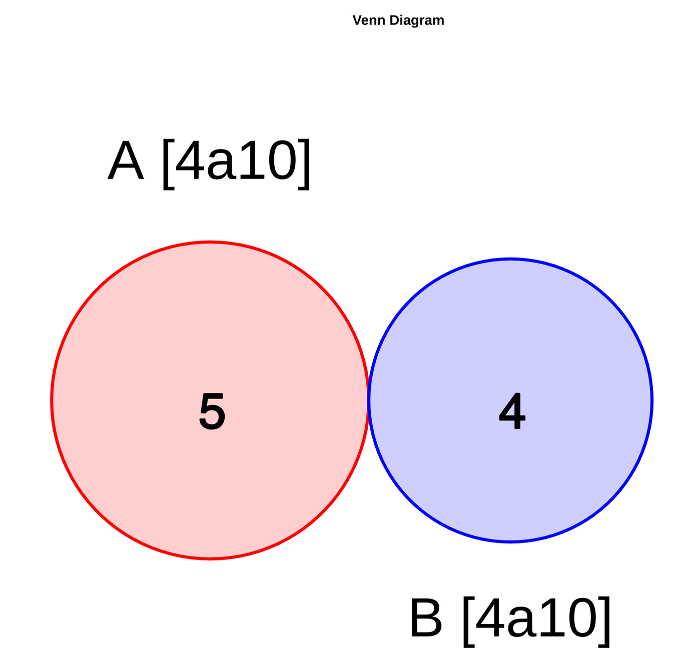

Venn Diagram

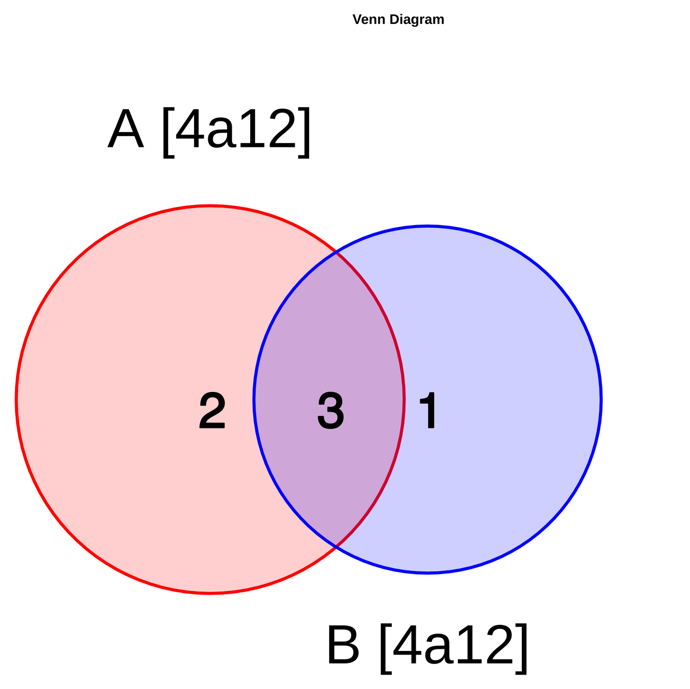

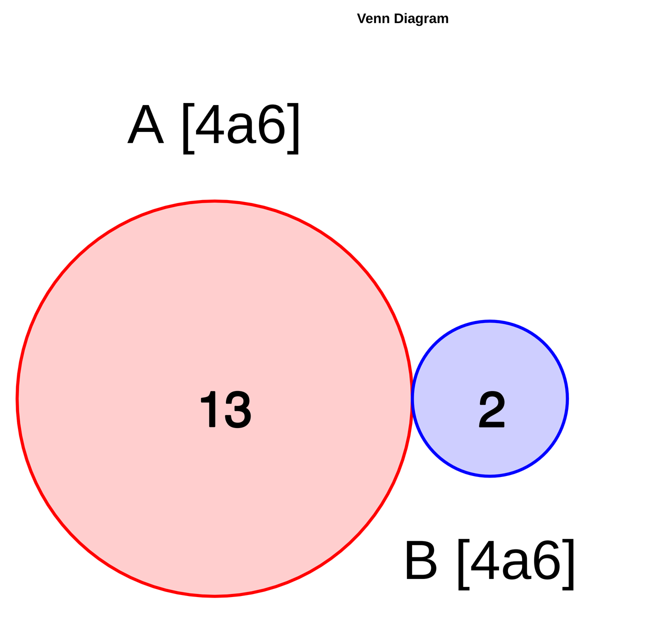

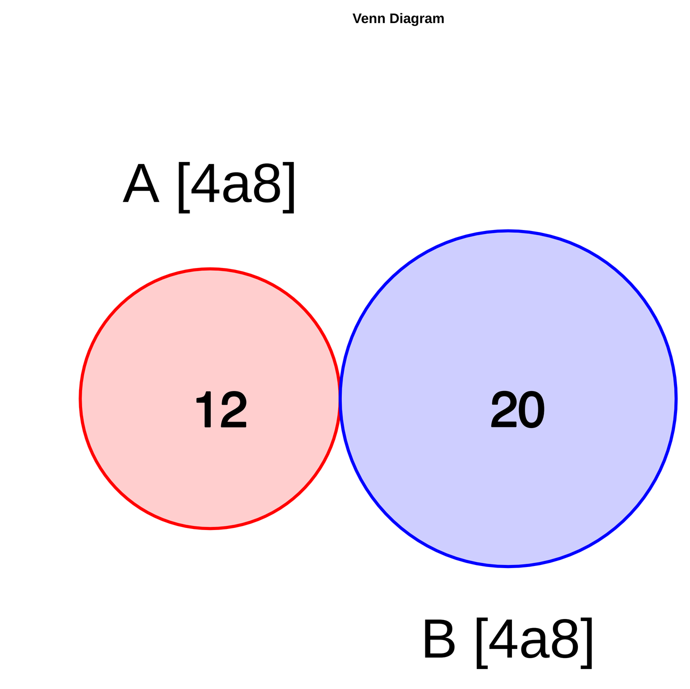

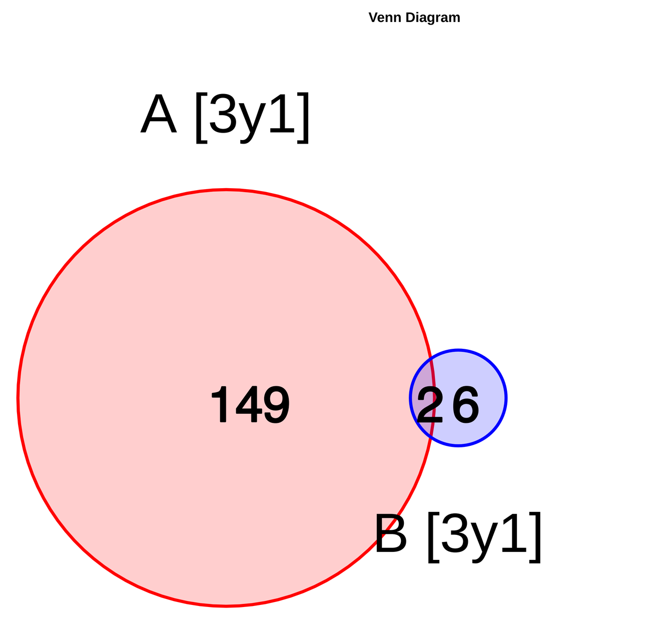

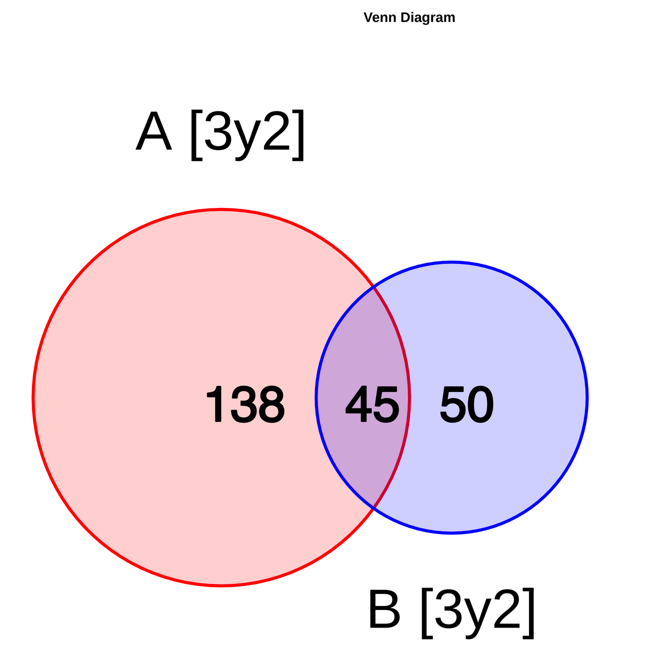

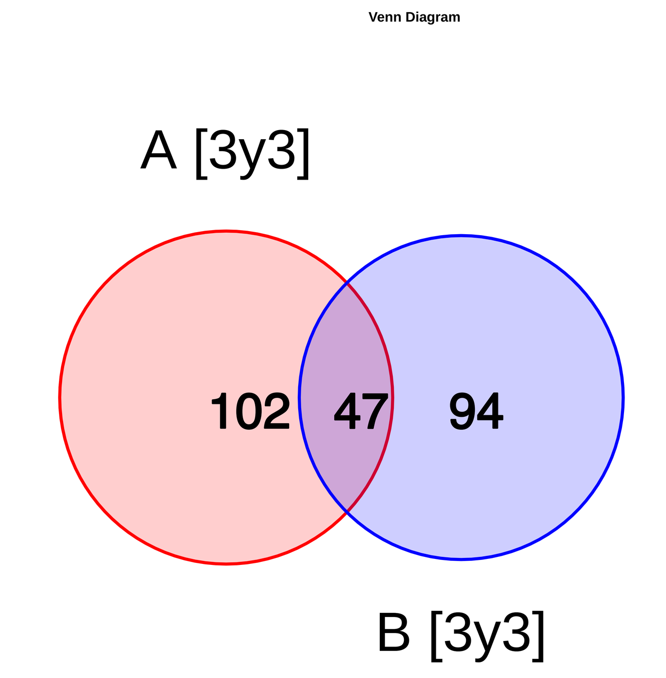

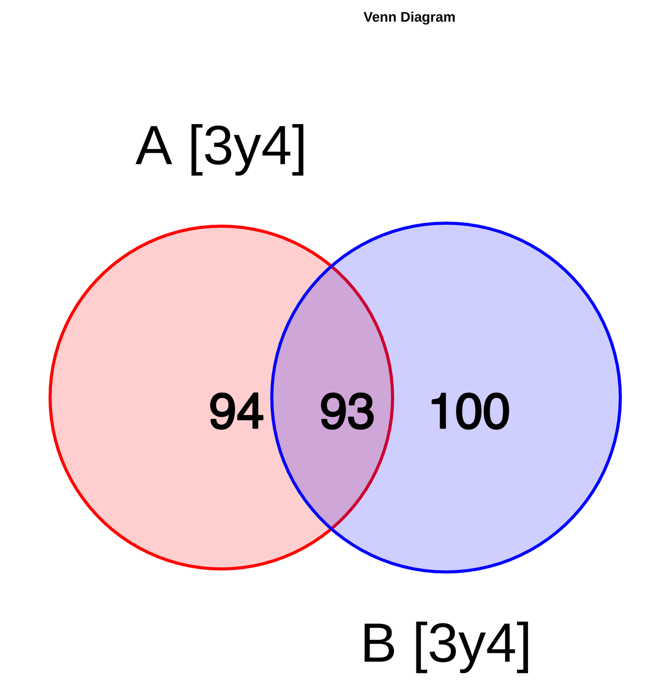

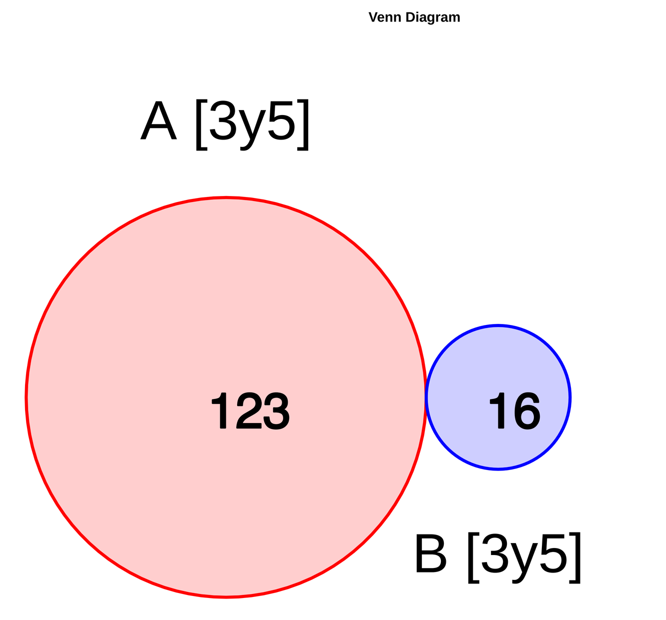

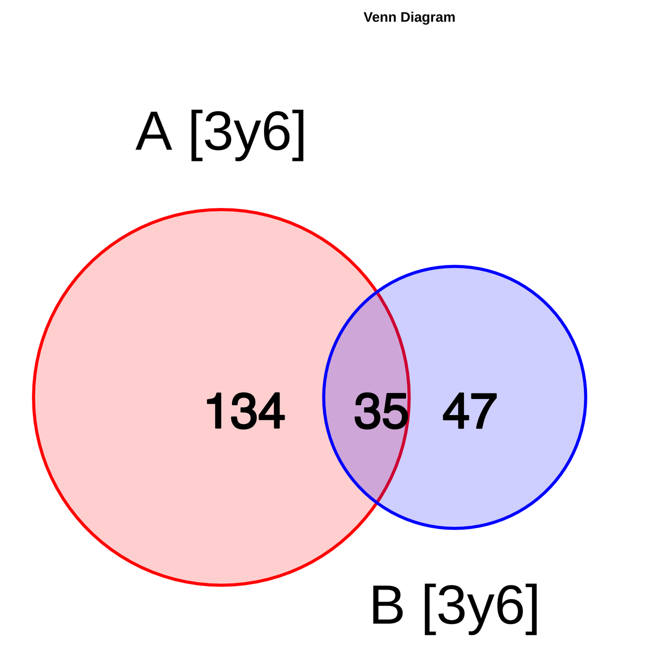

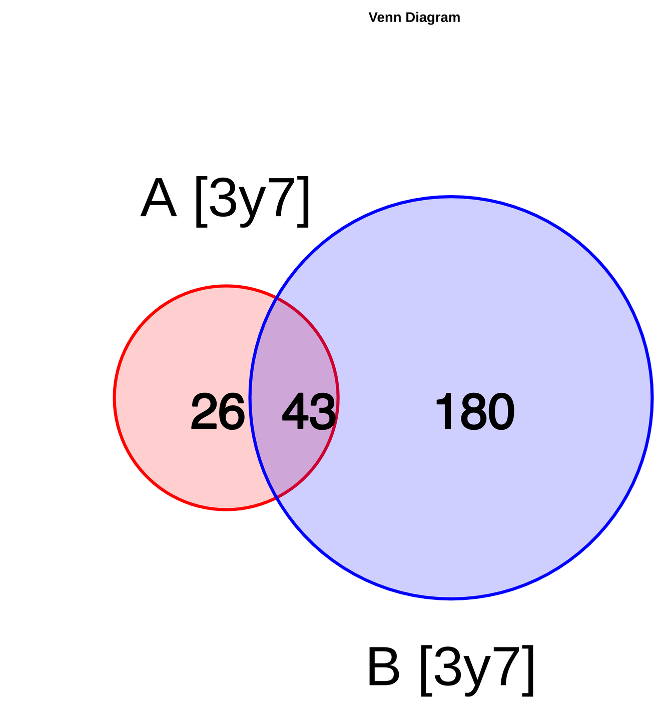

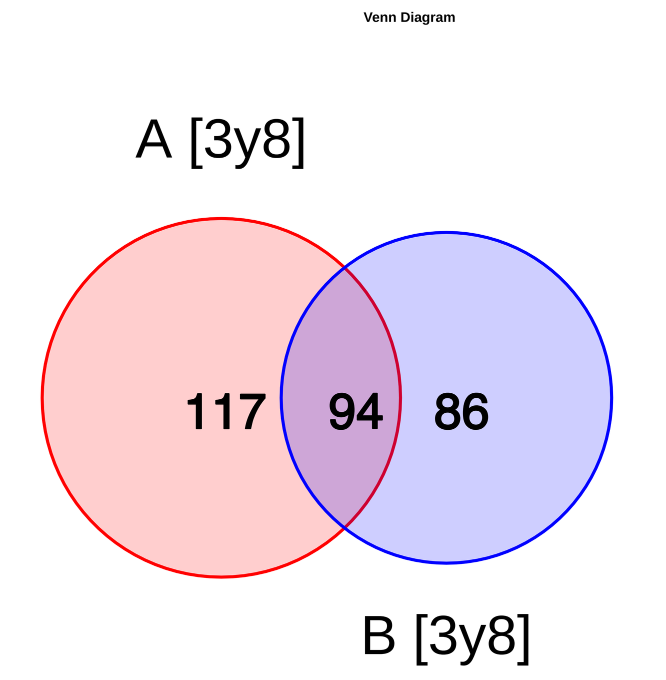

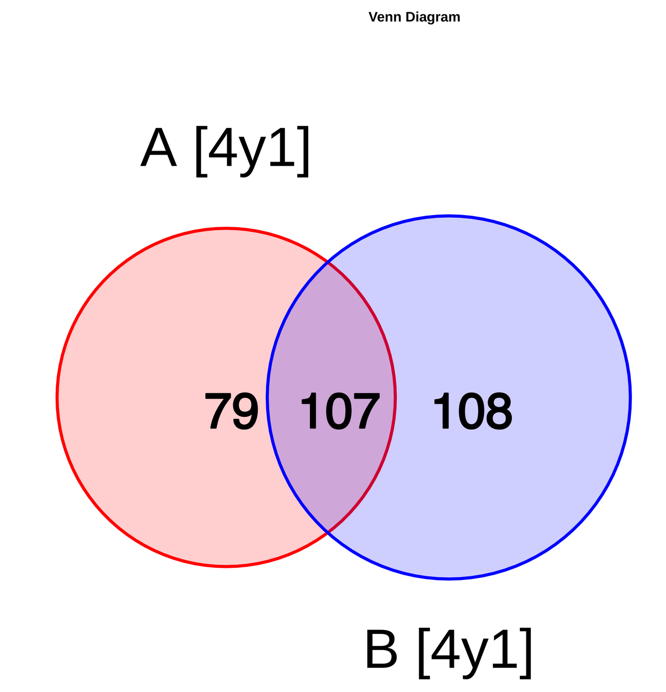

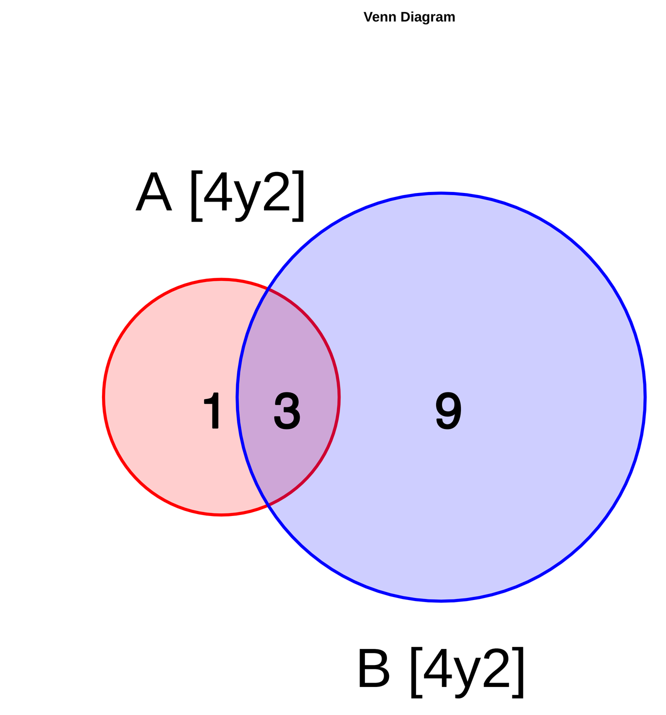

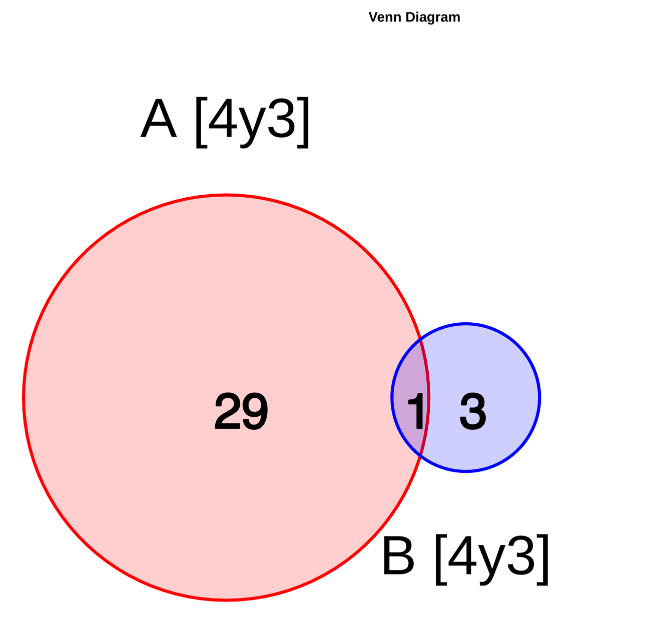

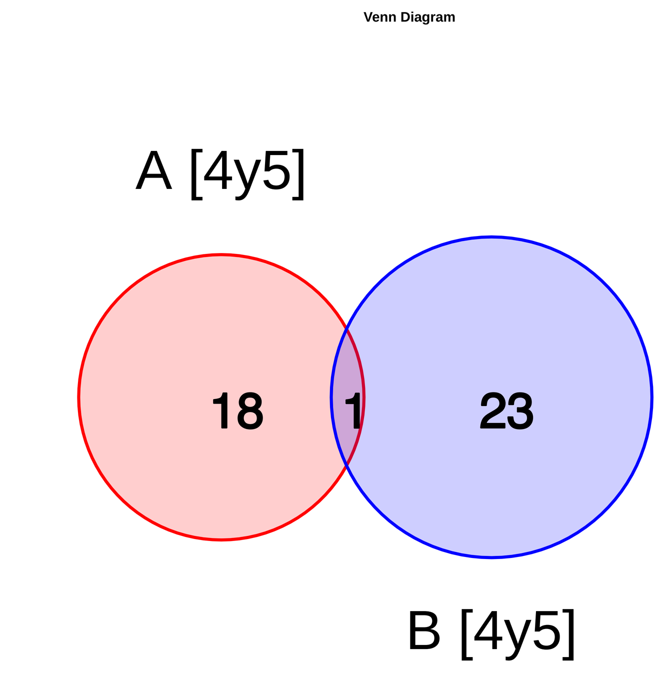

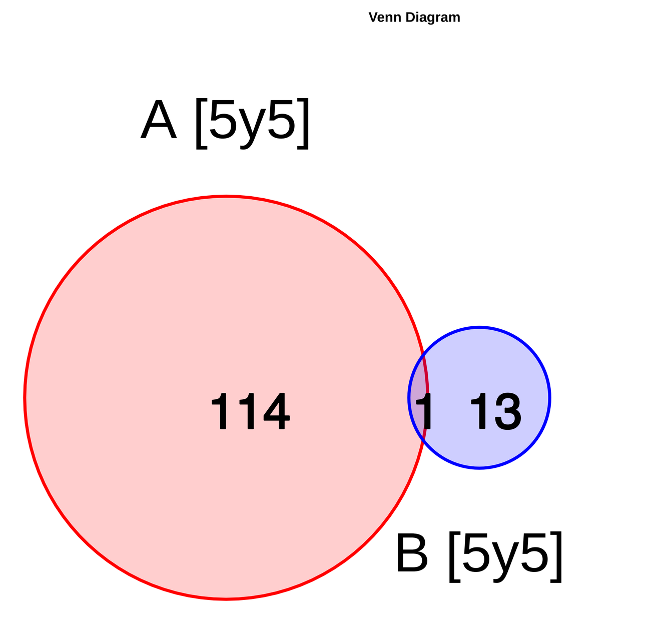

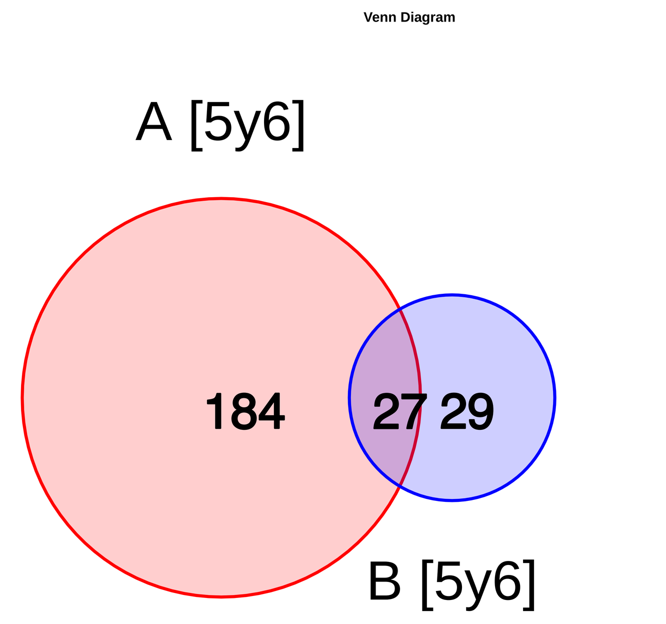

Supplement: S18 Fig — (PDF) [file pbio.2003389.s020.pdf]
