# Supplementary material for: Aging alters the epigenetic asymmetry of HSC division
Source: PLoS Biol. 2018 Sep 20;16(9):e2003389. doi: 10.1371/journal.pbio.2003389 (PMC6168157; doi:10.1371/journal.pbio.2003389)
Supplement: S1 Table — For each single sample set, the number and frequency of symmetric and asymmetric pairs is listed. The p-value refers to the chi-squared test. The decision on whether a cell divided symmetrically or asymmetrically was based on the ration of the Cdc42 volume in paired daughter cells (see graph Fig 1D). The threshold was set very conservatively to at least a one-fourth volume reduction (75% reduction in graph of Fig 1D): above the threshold, the division was scored as symmetric; below the threshold, it scored as asymmetric. (PDF) [file pbio.2003389.s021.pdf]

S1 Table. Analysis of frequency of symmetric/asymmetric divisions based on Cdc42 distribution to daughter cells – For each single sample set the number and frequency of symmetric and asymmetric pairs is listed. The p value refers to the Chi-square test. The decision on whether each single pair divided symmetrically or asymmetrically was taken based on the Cdc42 volume ratio between daughter cells (see graph Fig. 1d). The threshold was set to ¼ volume reduction (75% reduction in graph of Fig. 1d): above the threshold the division was scores as symmetric; below the threshold was defined asymmetric.

	**N of total daughter pairs (Cdc42)**	**N of asymmetric pairs**	**Frequency of asymmetric divisions**	**N of symmetric pairs**	**Frequency of symmetric divisions**	***p* value (Chi-square *vs* Young Ctr)**	***p* value (Chi-square *vs* Aged Ctr)**
**Young Ctr**	25	20	20/25 (80%)	5	5/25 (20%)	---	0.0011
**Aged Ctr**	26	9	9/26 (34.6%)	17	17/26 (65.4%)	0.0011	---
**Aged+CASIN**	26	15	15/26 (57.7%)	11	11/26 (42.3%)	0.0861	0.0951
**Young+Wnt5a**	14	6	6/14 (42.9%)	8	8/14 (57.1%)	0.0183	0.6076
