# Supplementary material for: Aging alters the epigenetic asymmetry of HSC division
Source: PLoS Biol. 2018 Sep 20;16(9):e2003389. doi: 10.1371/journal.pbio.2003389 (PMC6168157; doi:10.1371/journal.pbio.2003389)
Supplement: S2 Table — The outcome of each paired single daughter transplant is shown. Asym, asymmetric; DA, daughter A; DB, daughter B; n.e., not engrafted; P, cell retrospectively defined as progenitor; S, cell retrospectively defined as stem cell; SymS, symmetric self-renewal; SymD, symmetric differentiation. (PDF) [file pbio.2003389.s022.pdf]

S2 Table. Analysis of the divisional outcome - Single daughter pair transplants outcome for each sample set is shown. DA= daughter A; DB=daughter B. S = cell retrospectively defined as stem cell; P = cell retrospectively defined as progenitor. SymS= symmetric self-renewal; Asym=asymmetric; SymD=symmetric differentiation. n.e.=not engrafted.

	Young+Wnt5a			Young Ctr			Aged Ctr			Aged CASIN		
**Daughter pair n.**	DA	DB	Outcome	DA	DB	Outcome	DA	DB	Outcome	DA	DB	Outcome
**1**	S	S	SymS	P	S	Asym	S	S	SymS	P	S	Asym
**2**	S	S	SymS	S	S	SymS	S	S	SymS	S	P	Asym
**3**	S	S	SymS	S	P	Asym	S	S	SymS	S	S	SymS
**4**	P	S	Asym	P	S	Asym	S	S	SymS	n.e.	n.e.	--
**5**	S	S	SymS	S	P	Asym	S	S	SymS	n.e.	n.e.	--
**6**	n.e.	n.e.	--	S	S	SymS	P	S	Asym	P	S	Asym
**7**	n.e.	n.e.	--	P	S	Asym	S	n.e.	--	P	S	Asym
**8**	S	S	SymS	P	S	Asym	n.e.	P	--	S	P	Asym
**9**	S	S	SymS	n.e.	n.e.	--	P	S	Asym	n.e.	n.e.	--
**10**	S	S	SymS	P	S	Asym	S	S	SymS	n.e.	n.e.	--
**11**				S	S	SymS	S	S	SymS	P	S	Asym
**12**				S	P	Asym	n.e.	S	--	S	P	Asym
**13**				P	S	Asym	P	S	Asym	n.e.	P	--
**14**				n.e.	n.e.	--	S	S	SymS			
**15**				n.e.	S	--	P	P	SymD			
**16**				n.e.	n.e.	--	S	S	SymS			
**Total engraft**	8	8	8	12	14	12	14	15	13	8	9	8
**Efficiency pair**			80%			75%			81.25%			61.5%
