# Supplementary material for: Aging alters the epigenetic asymmetry of HSC division
Source: PLoS Biol. 2018 Sep 20;16(9):e2003389. doi: 10.1371/journal.pbio.2003389 (PMC6168157; doi:10.1371/journal.pbio.2003389)
Supplement: S3 Table — For each single sample set, the number and frequency of single and pair-wise mice are listed. The p-value refers to the chi-squared test based on the symmetric and asymmetric divisional outcomes. (PDF) [file pbio.2003389.s023.pdf]

S3 Table. Analysis of frequency of reconstitution – For each single sample set the number and frequency of single and pair wise mice is listed. The p value refers to the Chi-square test based on the symmetric and asymmetric divisional outcomes.

	**Mice reconstituted**	**Frequency**	**Mice reconstituted Pairs**	**Frequency pairs**	**Symmetric outcome**	**Asymmetric outcome**	***p* value (Chi-square *vs* Young Ctr)**	***p* value (Chi-square *vs* Aged Ctr)**
**Young Ctr**	26/32	81.25%	12/16	75%	3/12 (25%)	9/12 (75%)	---	0.0094
**Aged Ctr**	29/32	90.6%	13/16	81.25%	10/13 (76.9%)	3/13 (23.1%)	0.0094	---
**Aged+CASIN**	17/26	65.4%	8/13	61.5%	1/8 (12.5%)	7/8 (87.5%)	0.4936	0.0041
**Young+Wnt5a**	16/20	80.0%	8/10	80%	7/8 (87.5%)	1/8 (12.5%)	0.0062	0.5489
**Total**	88/110	80.0%	40/55	72.7%				
